# New beaked whales from the late Miocene of Peru and evidence for convergent evolution in stem and crown Ziphiidae (Cetacea, Odontoceti)

**DOI:** 10.7717/peerj.2479

**Published:** 2016-09-20

**Authors:** Giovanni Bianucci, Claudio Di Celma, Mario Urbina, Olivier Lambert

**Affiliations:** 1 Dipartimento di Scienze della Terra, Università di Pisa, Pisa, Italy; 2 School of Science and Technology, University of Camerino, Camerino, Italy; 3 Departamento de Paleontología de Vertebrados, Museo de Historia Natural–Universidad Nacional Mayor de San Marcos, Lima, Peru; 4 Direction Opérationnelle Terre et Histoire de la Vie, Institut royal des sciences naturelles de Belgique, Brussels, Belgium

**Keywords:** Cetacea, Peru, Neogene, Paleobiogeography, Fossil record, Ziphiidae, Evolution

## Abstract

The Ziphiidae (beaked whales) represent a large group of open-ocean odontocetes (toothed cetaceans), whose elusive and deep diving behavior prevents direct observation in their natural habitat. Despite their generally large body size, broad geographical distribution, and high species number, ziphiids thus remain poorly known. Furthermore, the evolutionary processes that have led to their extreme adaptations and impressive extant diversity are still poorly understood. Here we report new fossil beaked whales from the late Miocene of the Pisco Formation (southern Peru). The best preserved remains here described are referred to two new genera and species, the Messinian *Chavinziphius maxillocristatus* and the Tortonian *Chimuziphius coloradensis*, based on skull remains from two marine vertebrate-rich localities: Cerro Los Quesos and Cerro Colorado, respectively. *C. maxillocristatus* is medium sized retains a complete set of functional lower teeth, and bears robust rostral maxillary crests similar to those of the extant *Berardius*. By contrast, *C. coloradensis* is small and characterized by large triangular nasals and moderately thickened premaxillae that dorsally close the mesorostral groove. Both species confirm the high past diversity of Ziphiidae, the richest cetacean family in terms of the number of genera and species. Our new phylogenetic and biogeographical analyses depart markedly from earlier studies in dividing beaked whales into two major clades: the *Messapicetus* clade, which, along with other stem ziphiids, once dominated the southeastern Pacific and North Atlantic; and crown Ziphiidae, the majority of which are found in deep-water regions of the Southern Ocean, with possible subsequent dispersal both globally (*Mesoplodon* and *Ziphius*) and to the cooler waters of the northern oceans (*Berardius* and *Hyperoodon*). Despite this relatively clear separation, both lineages seem to follow similar evolutionary trends, including (1) a progressive reduction of dentition; (2) an increase in the compactness and thickness of the rostral bones; (3) similar changes in facial morphology (e.g., elevation of the vertex); and (4) an increase of body size. We suggest that these trends may be linked to a convergent ecological shift to deep diving and suction feeding.

## Introduction

Beaked whales (family Ziphiidae) are open ocean marine mammals capable of diving to depths up to nearly 3,000 m, where they find their prey (prevalently squid) using their sonar system and capture them via suction (Johnson et al., 2004; Mead, 2008; Schorr et al., 2014). With 22 extant species currently known, they are the second most diverse group of cetaceans after the delphinids (true dolphins). Because of their elusive and deep diving behavior, beaked whales are difficult to observe directly in their natural habitat. As a result, they are one of the most mysterious groups of mammals and unusually include several species only recently named or still to be described (Dalebout et al., 2002; Dalebout et al., 2014; Van Helden et al., 2002). Due to the scarce knowledge of their ecology, the evolutionary processes leading to their extreme adaptations and their impressive extant diversity are still poorly understood; only a few studies have been published on this topic until now. In one of these studies, sympatric sexual selection has been proposed to explain the diversification of extant species in the genus *Mesoplodon* (by far the most species-rich ziphiid genus), by mapping the patterns of tusk morphology of adult males on a molecular phylogenetic tree (Dalebout, Steel & Baker, 2008). The fossil record is another important resource for studying the evolutionary patterns of beaked whales. Up to a few years ago, fossil ziphiid taxa remained scarce and generally fragmentarily known. As a result of new discoveries, including some well-preserved specimens (e.g., fossils referred to the basal ziphiids *Messapicetus longirostris* Bianucci, Landini & Varola, 1992 from the late Miocene of Italy and *Notoziphius bruneti* Buono & Cozzuol, 2013 from the late Miocene of Argentina; see also Bianucci, Landini & Varola, 1994; Bianucci et al., 2016a) and a few, highly concentrated fossil assemblages, the ziphiid fossil record is now considerably improved. The most significant fossil assemblages have been unexpectedly discovered in phosphorite deposits from the seafloor off South Africa (Bianucci, Lambert & Post, 2007; Bianucci, Post & Lambert, 2008) and off the Atlantic coast of the Iberian Peninsula (Bianucci et al., 2013). These assemblages stand out for the high diversity of fossil genera recorded, several of which with bizarre skull features, as for example the huge spherical prominence formed by the premaxillae on the rostrum of *Globicetus* (Bianucci et al., 2013) or the combined high maxillary crests and voluminous and thick rostrum in *Africanacetus* (Bianucci, Lambert & Post, 2007; Gol’din & Vishnyakova, 2013; Gol’din, 2014). From inland outcrops, two significant ziphiid assemblages are from the Neogene marine deposits of the southern of the North Sea Basin (Antwerp, Belgium) and the Pisco-Sacaco basins (Peru). Most of the fossils from Antwerp were collected more than a century ago but have been recently reviewed, most being referred to the fossil genera *Aporotus*, *Beneziphius, Choneziphius*, and *Ziphirostrum* (Lambert, 2005). The best-preserved fossil ziphiids from the Neogene Pisco-Sacaco basins belong to three species: *Messapicetus gregarius* Bianucci, Lambert & Post, 2010, *Nazcacetus urbinai* Lambert, Bianucci & Post, 2009, and *Ninoziphius planirostris* Muizon, 1983 (see also Muizon, 1984; Lambert, Bianucci & Post, 2010; Lambert et al., 2015). Of these, *M. gregarius* and *N. urbinai* come from two fossil-rich, well-dated marine vertebrate localities—Cerro Colorado and Cerro Los Quesos—that have been investigated in great detail for the last few years, from both a paleontological (Bianucci et al., 2016b) and stratigraphic (Di Celma et al., 2016) point of view. As a result, all the fossil vertebrate specimens currently exposed at these two localities have been preliminarily identified in the field and reported in geological maps and related stratigraphic columns. Moreover, bio- and chrono-stratigraphic analyses allowed a precise dating for the two fossil assemblages. Detailed prospection lead to the discovery of several new ziphiid specimens. In Cerro Colorado, some of these consist of new remains of *M. gregarius*, including a partial skeleton associated to fish remains, the latter being interpreted as the last meal of the whale (Lambert et al., 2015); other skeletons of *M. gregarius*, including vertebrae and forelimb elements, are currently under study. From both Cerro Colorado and Cerro Los Quesos, significant ziphiid remains were found that belong neither to *M. gregarius* nor to *N. urbinai*. The first aim of this work is to describe in detail this new ziphiid material, including two skulls here referred to two new genera and species. Moreover a new phylogeny based on morphological characters is proposed here for beaked whales; the new topology has interesting implications for the discussion of (1) the evolutionary processes resulting in the high past and present diversity of the family, as well as (2) the peculiar paleobiogeographical patterns observed at a worldwide scale.

## Materials and Methods

The ziphiid specimens described here were discovered in beds of the Pisco Formation exposed at Cerro Colorado and Cerro Los Quesos localities during several field prospections from 2014 to 2016 and involving all the authors of this paper. The fossils were excavated by one of the authors (MU) and subsequently transported to the Museo de Historia Natural, Universidad Nacional Mayor de San Marcos, Lima. Their preparation and consolidation was made using mechanical tools and standard fossil vertebrate preparation techniques by W. Aguirre, under the scientific supervision of R. Varas-Malca in the Departamento de Paleontología de Vertebrados at MUSM.

The list of the specimens examined for comparisons and for the phylogeny follows Bianucci, Lambert & Post (2010), with the addition of material subsequently described by Bianucci et al. (2013), Bianucci et al. (2016a), Lambert, de Muizon & Bianucci (2013) and Lambert & Louwye (2016). Data for *Notoziphius* were taken from Buono & Cozzuol (2013). The cladistic analysis was modified from Lambert, de Muizon & Bianucci (2013), as detailed below. The new list of characters and resulting matrix are available as Supplemental Information (see File S1).

Anatomical terminology follows Mead & Fordyce (2009). Measurements mainly follow Ross (1984) and Lambert (2005).

### Nomenclatural acts

The electronic version of this article in Portable Document Format (PDF) will represent a published work according to the International Commission on Zoological Nomenclature (ICZN), and hence the new names contained in the electronic version are effectively published under that Code from the electronic edition alone. This published work and the nomenclatural acts it contains have been registered in ZooBank, the online registration system for the ICZN. The ZooBank LSIDs (Life Science Identifiers) can be resolved and the associated information viewed through any standard web browser by appending the LSID to the prefix http://zoobank.org/. The LSID for this publication is: urn:lsid:zoobank.org:pub:4CB30374-BA31-41CB-A2AB-D61BB5F084FB. The online version of this work is archived and available from the following digital repositories: PeerJ, PubMed Central and CLOCKSS.

## Results

### Systematic paleontology

Cetacea Brisson, 1762
Odontoceti Flower, 1867
Ziphiidae Gray, 1850

*Chavinziphius*, gen. nov.

**Type and only known species:** *Chavinziphius maxillocristatus*, sp. nov.

**Diagnosis:** As for the type species.

**Etymology:** From ‘Chavín,’ ancient culture of the north-central Andes and coastal area of Peru (900–200 BC), and from ‘*Ziphius*’ the type genus of the family. Gender masculine.

*Chavinziphius maxillocristatus*, sp. nov. (Figs. 2–6; Table 1)

**Table 1:** Measurements of the holotype cranium and mandible (MUSM 2538) of *Chavinziphius maxillocristatus*.

	Measurement (mm)
**Cranium**	
Condylobasal length	468^+^
Length of neurocranium	290
Width of rostrum base at level prominental notch	215
Width of rostrum base at level antorbital notch	225
Height of rostrum base at level antorbital notch	140
Width of premaxillae at level antorbital notch	88
Preorbital width of skull	340*
Postorbital width of skull	350*
Bizygomatic width of skull	368
Height of cranium	275
Length of antorbital process of lacrimal	48
Length of orbit	96
Length of temporal fossa	119
Height of temporal fossa	69
Total width of premaxillary sac fossae	110
Maximum width of right premaxillary sac fossa	53
Maximum width of left premaxillary sac fossa	44
Longitudinal distance left pmx foramen antorbital notch	36
Width of bony nares	55
Width left premaxillary crest	32
Minimum distance between premaxillary crests	62
Maximum width of nasals	78
Width of right nasal	38
Width of left nasal	36
Length of medial suture between nasals	37
Minimum posterior distance between maxillae on the vertex	69
Length of medial suture between frontals on the vertex	24
Minimum distance between temporal fossae in posterior view	235
Width of occipital condyles	114
Width of foramen magnum	35
Maximum width of right occipital condyle	42
Maximum height of right occipital condyle	63
**Mandible**	
Length of mandible	670^+^
Length of symphyseal portion of mandible	110^+^

**Notes:**

* indicates doubling of measurement from one side.

+ indicates preserved distance.

**Holotype and only referred specimen:** MUSM 2538, incomplete skull lacking most of the rostrum, the ear bones, the teeth, the left mandible, and the anterior portion of the symphyseal region of the right mandible.

**Figure 1:**
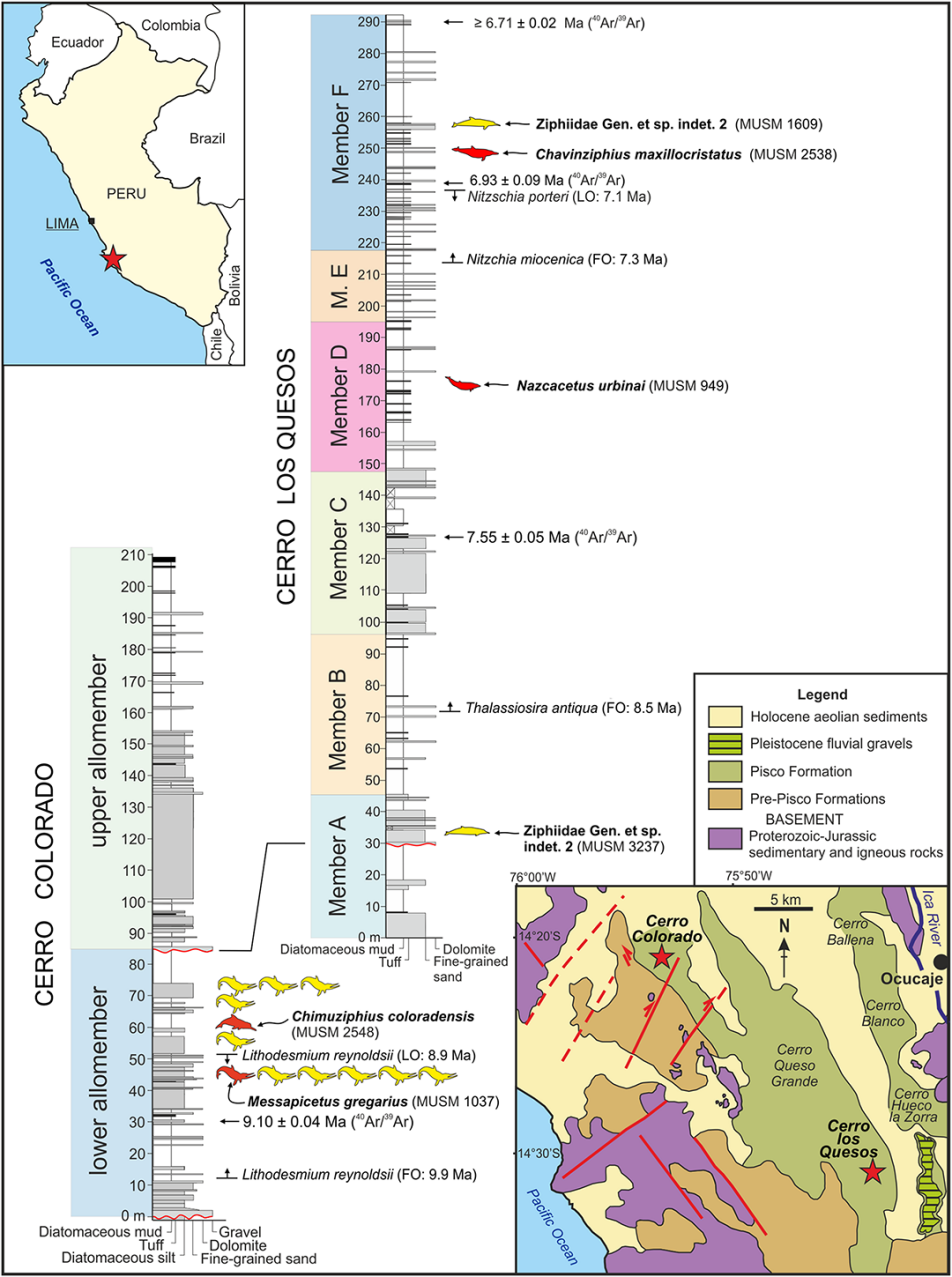
Locality and stratigraphy. Geographical position (stars) of Cerro Colorado and Cerro Los Quesos (Pisco Basin, southern coast of Peru) and related composite stratigraphic sections showing the distribution of fossil ziphiids, including the specimens described in this paper. Red silhouettes indicate holotypes; unnamed yellow silhouettes in the Cerro Colorado section indicate *Messapicetus gregarius* referred specimens. Biostratigraphical events and absolute dating constraining the age of the fossil ziphiids are also reportedalong the sections. Stratigraphical sections from Di Celma et al. (2016), bio- and chrono-stratigraphy from K. Gariboldi et al., 2015, unpublished data.

**Type locality:** Cerro Los Quesos, Pisco-Ica desert, 50 km south of the city of Ica, southern coast of Peru (Fig. 1). Geographic coordinates: 14°31′28.3″S–75°42′53.7″; 710 m above sea level. This specimen was reported in the Cerro Los Quesos fossil map (Bianucci et al., in press) with the field number O17 and provisionally referred to “Ziphiidae n.gen.2 n.sp.”

**Type horizon:** The holotype was found in Member F of the Pisco Formation as defined by Di Celma et al. (in press), about 10 m above a volcanic ash layer (the Mono key bed of Di Celma et al., in press) dated to 6.93 ± 0.09 Ma based on isotopic ^40^Ar/^39^Ar analyses. The stratigraphically higher tuff, exposed in the uppermost part of the Cerro Los Quesos section (42 m above the holotype) provides a minimum age of 6.71 ± 0.02 Ma (Di Celma et al., in press; K. Gariboldi et al., 2015, unpublished data), thus constraining the type horizon to a short interval of 0.22 Ma during the Messinian (latest Miocene). This age is further supported by biostratigraphic data, with the diatom assemblage below the Mono key bed correlating with the base of the *Nitzschia miocenica* zone (low-latitude diatom zonation of Barron (1985)), at an estimated age of 7.35–7.10 Ma (Di Celma et al., in press; K. Gariboldi et al., 2015, unpublished data). Most of the fossil vertebrate remains of Cerro Los Quesos have been recovered from the same horizon as MUSM 2538, and include odontocetes (e.g., the stem physeteroids *Acrophyster*, a kogiid similar to *Scaphokogia* and the phocoenid *Lomacetus*), mysticetes (balaenopteroids and cetotheriids), seals, crocodiles, seabirds, and sharks (Bianucci et al., in press; Lambert, Bianucci & de Muizon, 2016).

**Diagnosis:** *Chavinziphius* differs from all other ziphiids except *Berardius* and *Hyperoodon* in the presence of a robust and elevated longitudinal rostral maxillary crest extending from the posterior portion of the rostrum to the anterior portion of the neurocranium, posteromedial to the antorbital notch. Further differs from all other ziphiids in having the following combination of characters: anteroposteriorly elongated premaxillary sac fossa with premaxillary foramen distinctly anterior to the antorbital notch; ascending process of premaxilla gradually rising toward the vertex without ever becoming vertical; premaxillary crest inflated, oriented transversely, and partially in contact with the lateral margin of the nasal; moderate elevation of the vertex of the skull; moderate length of the temporal fossa; presence of three similarly-sized dorsal infraorbital foramina anterior to the base of the rostrum; presence of at least 50 small distinct alveoli for each mandible; unfused mandibles with a triangular cross-section of the symphyseal portion. Differs from Berardiinae in having a supraoccipital that reaches the top of the skull, and having a vertex with a less transversely constricted frontal exposure and no nodular protuberance formed by the interparietal or the frontals; from *Nenga*, *Pterocetus*, *Xhosacetus*, and Hyperoodontinae in lacking the inclusion of the nasal in the premaxillary crest; from Hyperoodontinae in lacking a deep anteromedial excavation of the nasals; from Hyperoodontinae except *Khoikhoicetus* in having premaxillary crest that is oriented transversely; from Ziphiinae (here restricted to *Ziphius* and *Izikoziphius*) in having shorter nasals, a less elevated vertex, less concave dorsal margin of the ascending process of premaxilla in lateral view, and a premaxillary crest that is transversely directed and forms a longer suture with the lateral margin of the nasal; from the *Messapicetus* clade (MC) as redefined here (i.e., including *Aporotus*, *Beneziphius*, *Chimuziphius, Choneziphius*, *Globicetus*, *Imocetus*, *Messapicetus*, *Notoziphius*, *Tusciziphius*, and *Ziphirostrum*) in lacking medial contacting and thickening of the premaxillae on the rostrum, and in having a premaxillary crest that is oriented transversely and forms a longer suture with the nasal; from *Nazcacetus* and *Tasmacetus* in having a premaxillary foramen that is located far anterior to the antorbital notch, a less concave dorsal margin of the ascending process of premaxilla in lateral view, a lesser transversely constricted exposure of the frontal on the vertex, a nasal that is not dorsally excavated,, and a dorsal margin of the mandible that gradually rises toward the coronoid process; and from *Ninoziphius* in having a premaxillary crest that is oriented transversely and forms a longer suture with the nasal, a less elevated vertex, and smaller and more numerous alveoli on the mandible.

**Etymology:** From Latin *maxilla* and *cristatus*, for having strong rostral maxillary crests at the base of the rostrum.

## Description and Comparisons

### Cranium

**Figure 2:**
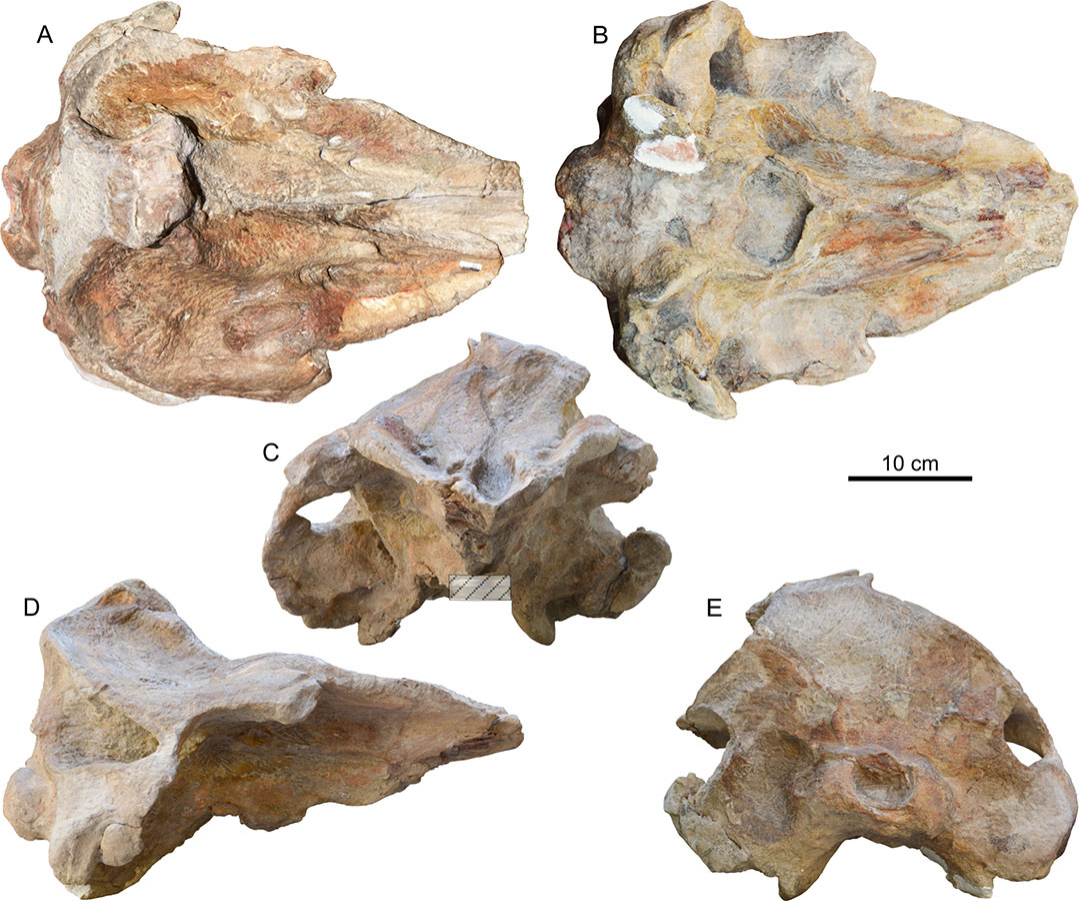
Cranium of *Chavinziphius maxillocristatus*. Cranium of the holotype (MUSM 2538) of *C. maxillocristatus*, from the Messinian of Cerro Los Quesos (Pisco Basin, Peru) in (A) dorsal, (B) ventral, (C) anterior, (D) right lateral, and (E) posterior view.

**Figure 3:**
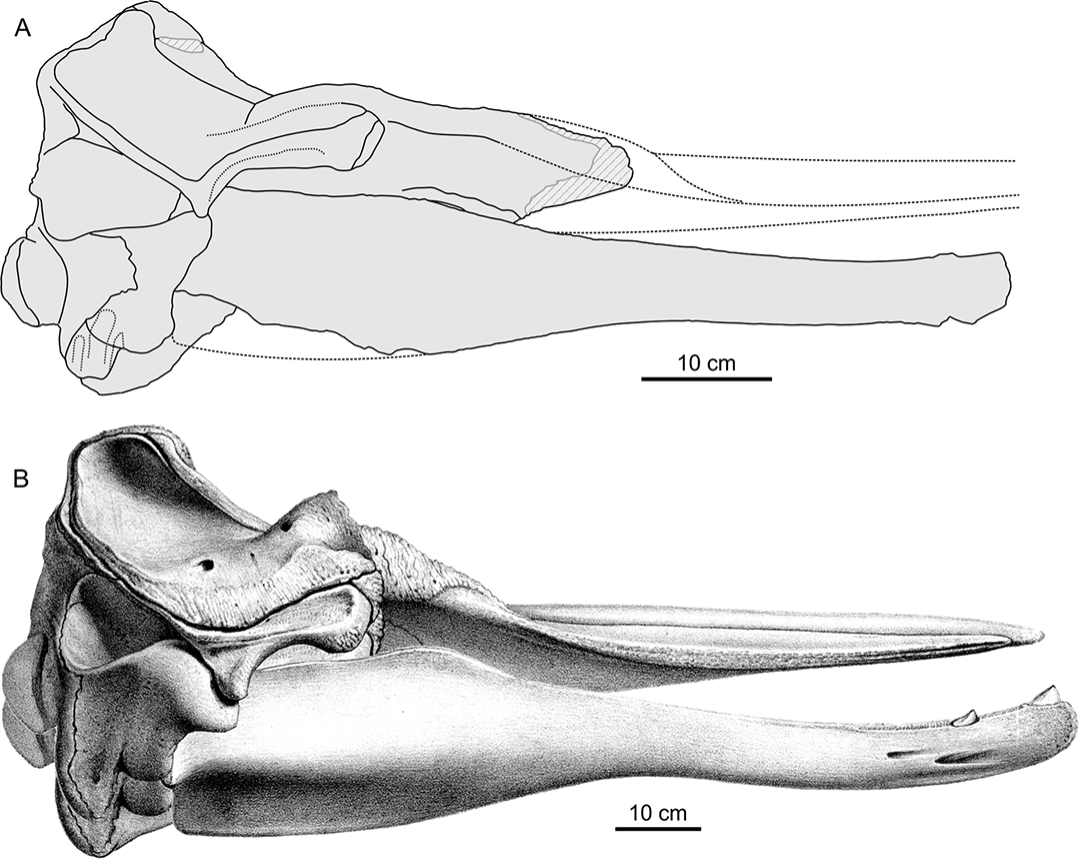
Comparison between *Chavinziphius maxillocristatus* and *Beradius arnouxii* skulls. Crania and articulated mandibles in right lateral view of *C. maxillocristatus* holotype (MUSM 2538) and *Berardius arnouxii* Duvernoy, 1851 (modified from Van Beneden & Gervais, 1868–1879: pl. 23), scaled with the same neurocranium length. Stippled lines for the reconstructed anterior part of the rostrum of *C. maxillocristatus*.

**Figure 4:**
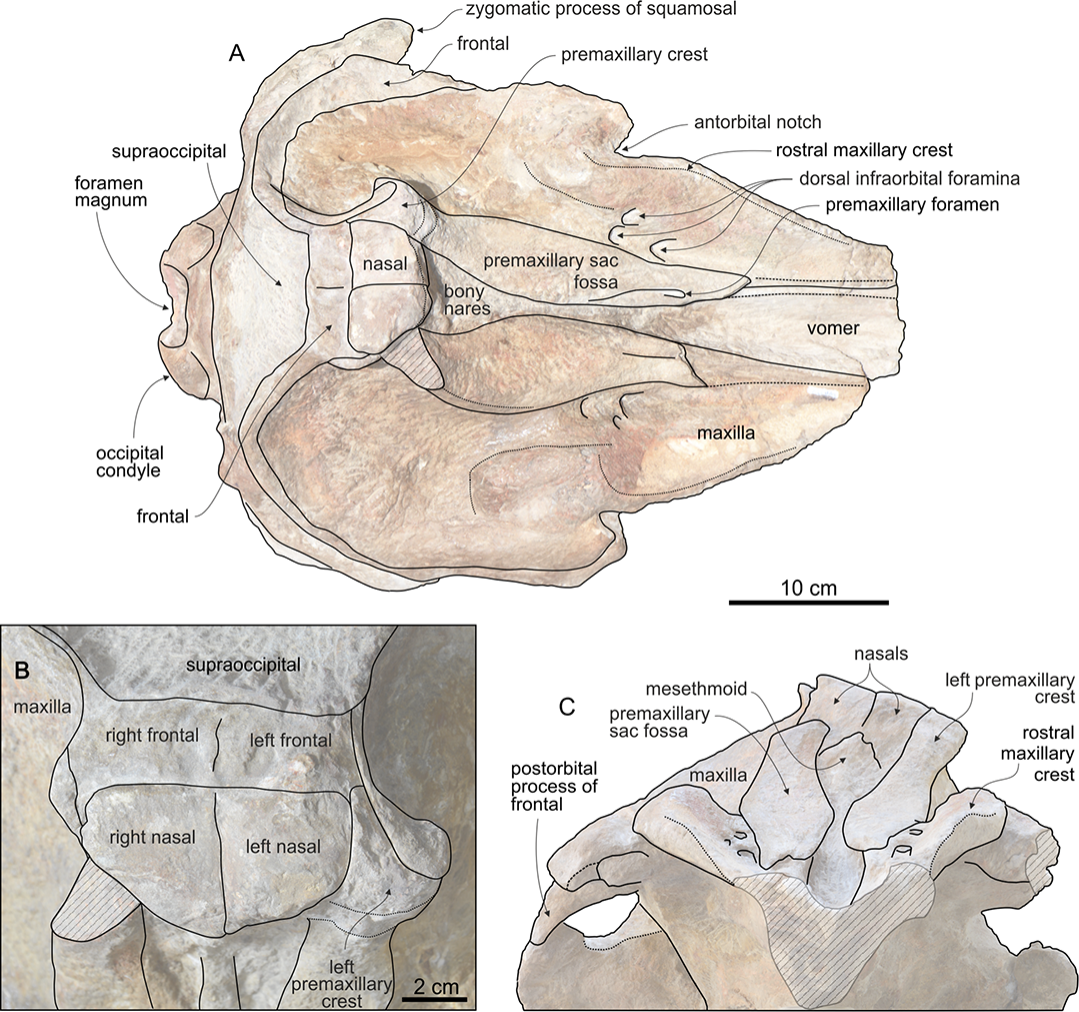
Cranium of *Chavinziphius maxillocristatus* in dorsal and anterior view. Cranium of the holotype (MUSM 2538) of *C. maxillocristatus*, from the Messinian of Cerro Los Quesos (Pisco Basin, Peru): (A) in dorsal view; (B) detail of the vertex area in dorsal view; and (C) in anterior view. Linear hatching indicates major breaks.

The cranium (Fig. 2) is medium sized compared with other ziphiids, with a postorbital width resembling that of extant *Mesoplodon bowdoini* Andrews, 1908 (see Bianucci, Post & Lambert, 2008: Table S2). Based on the nearly complete right mandible (Fig. 3), the rostrum appears to contribute at least 63% of the condylobasal length, which is longer than in *Beneziphius*, *Choneziphius planirostris* Cuvier, 1823, *Imocetus*, and *Ziphius*. The rostrum is broad at its base, transversely concave in anterior view owing to the robust rostral maxillary crests. The vertex is less elevated than in extant ziphiids (except *Berardius* and *Tasmacetus*), with the ratio of vertical distance from dorsal margin of the rostrum to top of the vertex to width of the premaxillary sac fossae = 0.88. The vertex is clearly shifted towards the left (Fig. 4C), even when taking into account potential postmortem deformation (a fracture is visible on the left side of the posterior surface: Fig. 2E). The temporal fossa is moderately elongated (ratio of horizontal length of temporal fossa to length of neurocranium = 0.41), intermediate between the more elongated fossae of *Messapicetus* (0.52–0.54) and *Tasmacetus* (0.48), both of which bearing functional teeth, and the shorter fossae of the nearly edentulous *Berardius*, *Hyperoodon*, *Mesoplodon*, and *Ziphius* (close to 0.30).

#### Premaxilla

**Figure 5:**
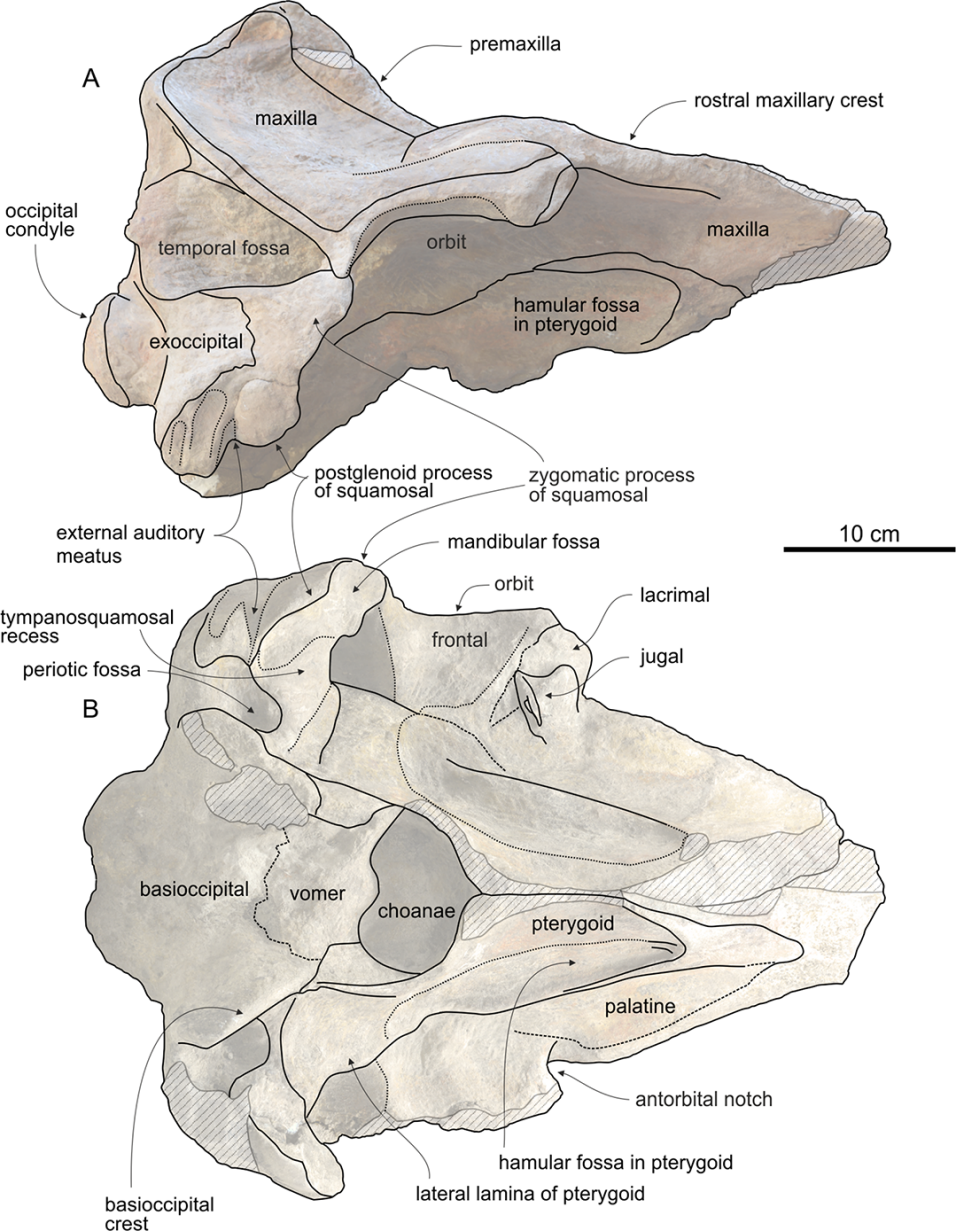
Cranium of *Chavinziphius maxillocristatus* in lateral and ventral view. Cranium of the holotype (MUSM 2538) of *C. maxillocristatus*, from the Messinian of Cerro Los Quesos (Pisco Basin, Peru) in (A) right lateral, and (B) ventral view. Linear hatching indicates major breaks.

Along the dorsal surface of the short preserved proximal portion of the rostrum (ca 17 cm long) the premaxillae are only partly preserved (Fig. 4A). Judging from these small fragments and considering the great width of the mesorostral groove, the premaxillae were most likely not thickened on the rostrum, with a partly dorsally open mesorostral groove differing from all members of the *Messapicetus* clade and from *Chimuziphius* (see below). One small left premaxillary foramen is located 36 mm anterior to the level of the antorbital notch, at the anterior end of a shallow posteromedial sulcus. The premaxillary sac fossa is anteroposteriorly elongated and weakly transversely convex; the right premaxillary sac fossa is slightly wider than the left (ratio between the widths of left and right fossae = 0.82). The premaxillary foramen being located anterior to the antorbital notch and the related anteroposterior elongation of the premaxillary sac fossa are two features observed in some specimens of the extant *Indopacetus* and in several fossil ziphiids (*Messapicetus* and all other ziphiids characterized by the presence of a prenarial basin, *Ninoziphius*, *Pterocetus*, probably *Notoziphius*, and *Chimuziphius*). The lateral margin of the premaxillary sac fossa is higher than the adjacent dorsal surface of the maxilla, although there is no overhanging of the maxilla by the premaxilla (a difference with *Choneziphius*, *Izikoziphius*, and *Ziphius*) and no clear step, only a smooth transition from the premaxilla to the maxilla. The ascending process of the premaxilla gradually rises toward the vertex and is moderately concave in lateral view, not reaching the vertical in its posterodorsal portion (Fig. 5A). A similar low elevation of the ascending process is observed in *Berardius* and related fossil species, as well as in several stem beaked whales (*Ninoziphius* and *Notoziphius*). At half its height, the ascending process of the premaxilla exhibits a transverse constriction (Fig. 4C); such a feature is absent in *Berardius*, *Ziphius*, and related fossil species. An even more pronounced constriction is instead observed in *Hyperoodon*, *Mesoplodon* and other hyperodoontines, *Tasmacetus*, and several other fossil beaked whales (e.g., *Messapicetus* and *Tusciziphius*). The preserved left premaxillary crest is laterally directed, as in *Berardius* and fossil berardiines, *Tasmacetus*, and the fossil genera *Nazcacetus* and *Nenga*. This crest is inflated, with the anterior margin being convex as in *Berardius* and *Tasmacetus*; it is anteroposteriorly thicker than in *Nazcacetus* and particularly *Archaeoziphius*.

#### Maxilla

The dorsolateral margin of the rostral maxilla exhibits an elevated longitudinal crest, running along the whole preserved portion of the rostrum and extending posteromedial to the antorbital notch in the supraorbital region, as a large dome-like crest (Fig. 4A). A similar rostral maxillary crest is present in *Berardius* (same robustness than in *Chavinziphius*), *Tasmacetus* (somewhat lower), and, although lower and shorter, *Indopacetus*, *Izikoziphius rossi* Bianucci, Lambert & Post (2007), *Xhosacetus*, *Ziphius*, and some species of *Mesoplodon*. A different architecture of the rostral maxillary crests is observed in adults of *Hyperoodon*: huge rostral crests converge posteromedially and are clearly distinct from the relatively lower maxillary crests above the orbits. Distinct rostral maxillary crests and maxillary crests (as defined by Mead & Fordyce (2009)) are also observed in *Imocetus*; however, the latter differs from *Hyperoodon* in having posteriorly shorter rostral maxillary crests (not reaching the base of the rostrum), posteriorly diverging, and with a peculiar spur-like posterior projection. Several other ziphiids differ from *Chavinziphius* in having a dome-like crest limited to the supraorbital area (the greatest crest being observed in *Africanacetus*). In the rostrum base region, the depressed medialmost portion of the dorsal surface of the maxilla is pierced by a cluster of three infraorbital foramina; those foramina open anterodorsally and the largest have a transverse diameter reaching 5–7 mm. In detail, on the right maxilla the largest foramen is located at the anteroposterior level of the antorbital notch, just anteromedial to the smaller foramen, whereas the foramen intermediate in size is ca 20 mm posterior to the antorbital notch. On the left maxilla, three foramina with a roughly similar size are located: 1) ca 25 mm anterior to the antorbital notch; 2) ca 8 mm anterior to the antorbital notch and slightly lateral to the two other foramina; 3) at about the level of the antorbital notch. A cluster of infraorbital foramina in the rostrum base region may constitute the plesiomorphic condition among ziphiids. In most extant ziphiids only a large foramen is present, proposed here to result from the merging of several small foramina. This hypothesis is supported by the observation of a cluster of foramina in immature specimens of *Berardius* and *Messapicetus gregarius*, contra a single foramen in the respective adult specimens (see File S2 and Bianucci, Lambert & Post (2010)). No traces of posterior dorsal infraorbital foramina are observed in any of the ascending processes of the maxillae. Because the preserved palatal surface of the maxilla (Fig. 5B) is damaged, the presence of an upper alveolar groove and its posterior extent cannot be assessed. When the mandible is articulated to the skull, the posteriormost lower alveolus is located ca 100 mm anterior to the level of the antorbital notch.

#### Vomer

A wide portion of the vomer is exposed dorsally between the partly broken premaxillae along the preserved posterior part of the rostrum, forming the ventral surface of the mesorostral groove (Fig. 4A). The vomer does not appear to be thickened in this area, therefore not filling the mesorostral groove and differing from many species of *Mesoplodon*.

#### Nasal

The dorsal outline of large nasals is trapezoidal, less anteroposteriorly elongated than in *Berardius*, *Tasmacetus*, and particularly *Ziphius* (Fig. 4B). The lateralmost margins of the nasals are subparallel, as in *Nenga* and *Xhosacetus*, not posteriorly convergent as in *Berardius*, *Tasmacetus*, and, to a lesser extent, *Nazcacetus*. Such a difference is related to the lesser transverse constriction of the posterior part of the vertex in *Chavinziphius*. The anterior margin of the nasals is not excavated as in the Hyperoodontinae and related species; it forms an anterior point with an obtuse angle (ca 120°), similar to *Aporotus dicyrtus* du Bus, 1868 (Lambert, de Muizon & Bianucci, 2013: Fig. 5), but more obtuse than in *Berardius*, *Chimuziphius*, *Nazcacetus*, *Tasmacetus*, and *Xhosacetus* (all with an angle close to 90°) and even more obtuse than in *Nenga* and *Notoziphius* (< 90°). The nasal contacts the premaxilla laterally for a long distance, but not for the whole length of the former, a condition shared with *Archaeoziphius*, *Berardius*, *Indopacetus*, *Microberardius*, *Nazcacetus*, *Nenga*, and *Tasmacetus*. The nasal does not expand laterally inside the premaxillary crest, differing from *Nenga*, Hyperoodontinae, and related fossil taxa. The posterior nasal-frontal suture is straight and transversely oriented as in *Nenga*, not posteriorly convex as in *Berardius* and *Nazcacetus*, or posteriorly concave as in *Tasmacetus*. The dorsal surface of the nasals is roughly flat, not anteroventrally sloping, with only a shallow longitudinal depression following the straight medial suture between the nasals (Figs. 4B and 4C).

#### Frontal

The anteroposterior length of the orbit (Fig. 5A) makes 30% of the postorbital width of the skull, a value close to most other ziphiids, but proportionally distinctly larger than in the larger-bodied *Berardius* and *Hyperoodon* (ca 20%) and smaller than in the holotype of *Nazcacetus urbinai* (ca 50%). The preorbital process of the frontal is dorsoventrally thickened, but less than in *Berardius* and *Hyperoodon*. The postorbital process is triangular and short, with the ventral tip contacting the dorsolateral surface of the apex of the zygomatic process of the squamosal. On the vertex, although anteroposteriorly short the frontals are conspicuous between the nasals and the supraoccipital (Fig. 4B). They are moderately transversely compressed between the maxillae, more than in the early branching ziphiids *Messapicetus* and *Notoziphius*, but less than in Berardiinae (*Archaeoziphius*, *Berardius*, and *Microberardius*), *Nazcacetus*, and *Tasmacetus*. Differing from Berardiinae, no nodular prominence formed by the interparietal or frontals is observed in the posterior part of the vertex.

#### Supraoccipital

The supraoccipital wedges anteriorly between the two ascending processes of the maxillae; its broad dorsal exposure is trapezoidal in outline, with a straight anteromedial suture with the frontals (Fig. 4A). A similar shape of the anterior margin of the supraoccipital is observed in *Messapicetus*, whereas in most other ziphiids this margin is more triangular, with an anteromedial point. The anteromedial margin of the supraoccipital reaches the level of the top of the vertex, as in all other ziphiids except *Archaeoziphius* and *Berardius*.

#### Basioccipital and exoccipital

The occipital condyles are rather protuberant, delimiting a circular foramen magnum (Figs. 3A, 3B and 3E). The basioccipital basin is wide, laterally defined by basioccipital crests forming together an angle of 66° (Fig. 5B). Broadly diverging basioccipital crests are observed in all ziphiids except *Messapicetus* and a referred specimen (MNHN SAS 1628) of *Ninoziphius*.

#### Palatine and pterygoid

The maxilla-palatine suture is not clearly visible: it probably runs parallel to the lateral margin of the rostrum, with the palatine covering most of the ventral surface of the maxilla lateral to the pterygoid (Fig. 5B). The large fossa for the hamular lobe of the pterygoid sinus extends anteriorly on the palatal surface of the rostrum beyond the level of the antorbital notch, as in all ziphiids having this skull portion preserved.

#### Lacrimal and jugal

The ventral surface of the preserved right lacrimal is partly covered by an articulated fragment of the anterior portion of the jugal (Fig. 5B). The suture between these two bones is visible.

#### Squamosal

In lateral view, the squamosal has a typical ziphiid shape (Fig. 5A). Indeed, as in all ziphiids having this part of the skull preserved the zygomatic process of the squamosal is anteroposteriorly short compared to its dorsoventral height, and the ventral margin of the postglenoid process of the squamosal is distinctly more dorsal than the ventral margin of the paroccipital process of the exoccipital.

### Mandible

**Figure 6:**
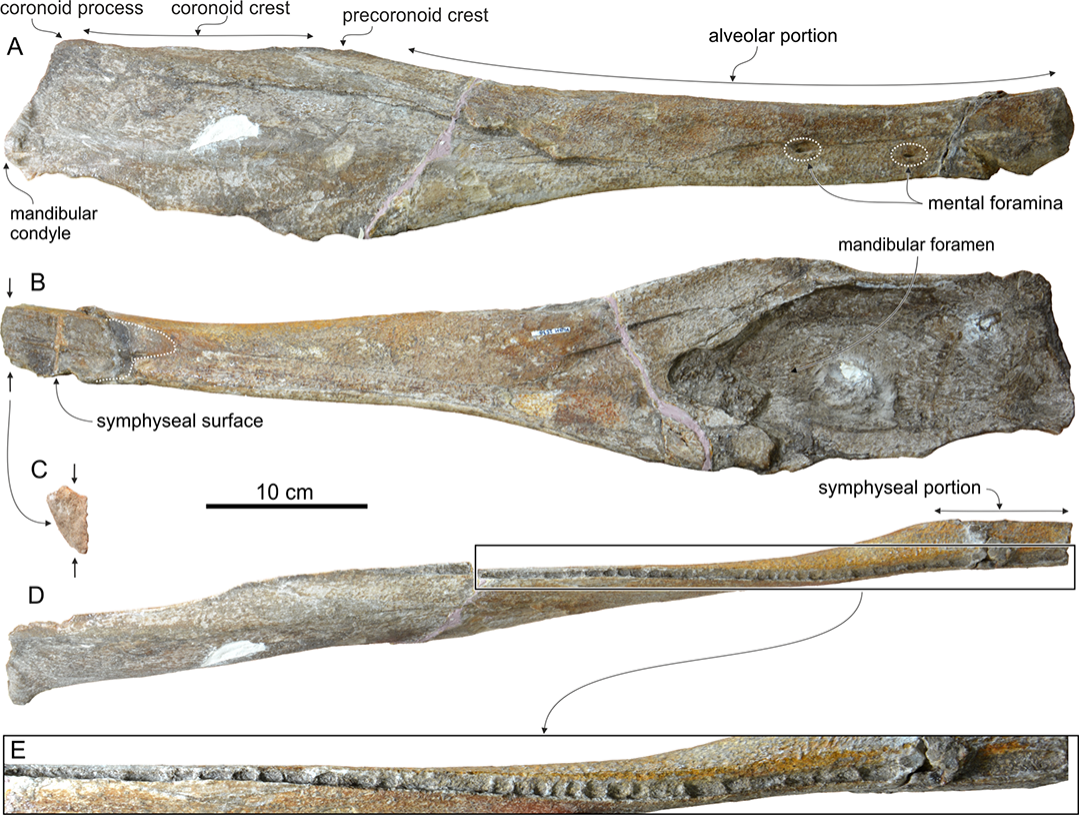
Mandible of *Chavinziphius maxillocristatus*. Right incomplete mandible of the holotype (MUSM 2538) of *C. maxillocristatus*, from the Messinian of Cerro Los Quesos (Pisco Basin, Peru) in (A) lateral, (B) medial, (C) anterior, and (D, E) dorsal view.

Only the incomplete right mandible is preserved (Fig. 6). Since the two mandibles separated along the symphyseal surface, clearly discernible on the anterior portion of the medial surface of the mandible (Fig. 6B), the symphysis was originally unfused. Fused mandibles are instead observed in *Messapicetus*, *Ninoziphius*, and *Tasmacetus*. The symphyseal portion makes more than 17% of the total length of the mandible (underestimated, the anterior part of the mandible being missing). The reconstructed transverse section of the joined mandibles along the symphyseal portion is triangular and much higher than wide (Fig. 6C), differing from the semicircular section of *Berardius*, *Messapicetus*, *Ninoziphius*, and *Tasmacetus*.

The preserved portion of the mandible bears an alveolar groove with ca 50 small alveoli (Fig. 6E). This minimum estimate for the lower tooth count is higher than in all other ziphiids with the mandible preserved and a complete functional dentition: *Messapicetus* (25–26; Bianucci et al., 2016a); *Ninoziphius* (40–42; Lambert, de Muizon & Bianucci, 2013), and *Tasmacetus* (18–28; Mead & Payne, 1975). Moreover, the anteroposterior length of the postsymphyseal portion of the alveolar groove is proportionally longer (54% of the postsymphyseal length) than in the taxa mentioned above (*Messapicetus* 34%, *Ninoziphius* 38%, and *Tasmacetus* 22%). The transverse width of the alveolar groove is 6 mm along most of the symphyseal portion; it narrows (4 mm) at the posterior end of the symphysis and widens slightly (5 mm) along the post-symphyseal portion. The alveoli are circular to weakly anteroposteriorly elongated and they are separated by thin septa (ca 2 mm thick). Considering these small alveoli, it is probable that the mandibular teeth of *Chavinziphius* were close in size to the teeth of *Nazcacetus*, having a diameter ranging from 2.5 to 4 mm (Lambert, Bianucci & Post, 2009). Although the postapical teeth of *Chavinziphius* were distinctly smaller than the teeth of *Messapicetus*, *Ninoziphius*, and *Tasmacetus*, the well defined mandibular alveoli indicate that they were firmly rooted in the mandible and thus most likely functional, contrasting with the hypothesis of vestigial postapical teeth held in the gum for *Nazcacetus*. The anterior portion of the mandible of *Chavinziphius* being missing, the presence of large alveoli for apical or subapical tusks—a derived character shared by all extant ziphiids and the fossil ziphiids for which this region is associated to cranial material (*Messapicetus*, *Nazcacetus*, and *Ninoziphius*)—cannot be assessed.

In lateral view, the mandible is relatively robust (Fig. 6A). At the posterior end of the symphysis, it displays a dorsoventral thickening also observed in *Nazcacetus* and, among extant species, in adult males of *Ziphius* and some species of *Mesoplodon* (e.g., *M. bidens* Sowerby, 1804 and *M. bowdoini*). In the latter taxa, this thickening is related to the anterodorsal curve of the anterior part of the symphyseal portion (not preserved in *Chavinziphius*).

On the lateral surface of the mandible, two small mental foramina are located at the level of the posterior end of the symphysis and 70 mm posterior, respectively.

The minimum height of the mandible is just posterior to the symphysis. From there, the height increases gradually towards the posterior end of the alveolar portion. The dorsal margin of the mandible reaches a maximum elevation 40 mm posterior to the last alveolus, forming a low but distinct precoronoid crest as observed in all other ziphiids (Lambert, Bianucci & Post, 2009, Lambert, de Muizon & Bianucci, 2013; Bianucci, Lambert & Post, 2010), but also in some delphinids (Fordyce, Quilty & Daniels, 2002; Bianucci, 2013). If the dorsal margin of the alveolar portion is positioned parallel to the horizontal plane, the precoronoid crest of *Chavinziphius* reaches approximately the same dorsoventral elevation as the coronoid process, with the dorsal margin of the coronoid crest being weakly concave. A higher precoronoid crest associated to an abrupt dorsal elevation of the whole dorsal margin of the mandible posterior to the alveolar groove is present in *Berardius* (Fig. 3) and, even more pronounced, in *Nazcacetus* and *Tasmacetus*. The coronoid crest of *Chavinziphius* is long compared to most other ziphiids; with the mandible articulated to the skull, the top of the precoronoid crest is located 40 mm anterior to the antorbital notch. Similarly, the mandibular foramen is anteroposteriorly elongated, with its anterior margin 60 mm posterior to the end of the coronoid crest. A more anterior extension of the coronoid crest and mandibular foramen is seen in *Tasmacetus* and *Nazcacetus*, whereas in *Ninoziphius* both the coronoid crest and the mandibular foramen are anteriorly shorter than in *Chavinziphius*.

The mandibular condyle is not protuberant, a condition shared with most other ziphiids, except *Messapicetus gregarius*, *Ninoziphius*, and, at least for some specimens, *Tasmacetus*. All the latter taxa display a conspicuous notch between the angular process and the mandibular condyle (Lambert, de Muizon & Bianucci, 2013). Such a notch is absent in *Chavinziphius*.

*Chimuziphius*, gen. nov.

**Type and only known species:** *Chimuziphius coloradensis*, sp. nov.

**Diagnosis:** As for the type species.

**Etymology:** From ‘Chimú,’ ancient culture of the northern coastal area of Peru (1100–1470 AD), and from ‘*Ziphius*,’ the type genus of the Ziphiidae. Gender masculine.

**Figure 7:**
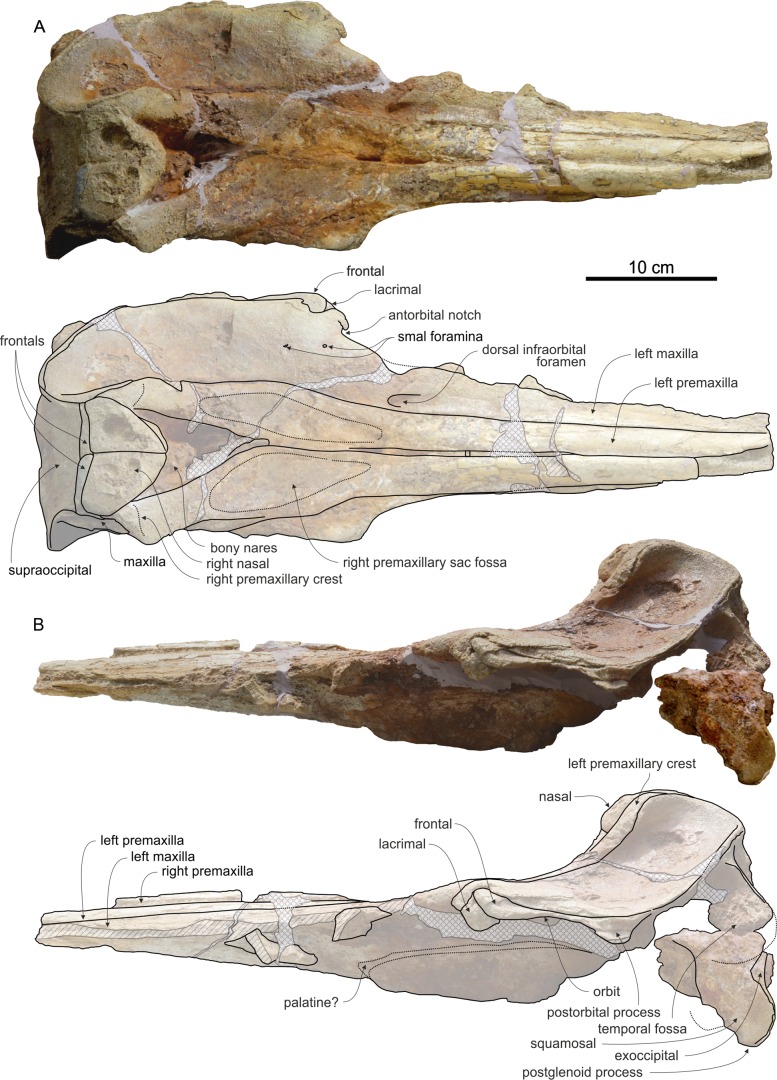
Cranium of *Chimuziphius coloradensis* in dorsal and lateral view. Cranium of the holotype (MUSM 2548) of *C. coloradensis*, from the Tortonian of Cerro Colorado (Pisco Basin, Peru) in (A) dorsal, (B) left lateral view. Linear hatching indicates major breaks, cross hatching indicates reconstructed missing parts.

**Figure 8:**
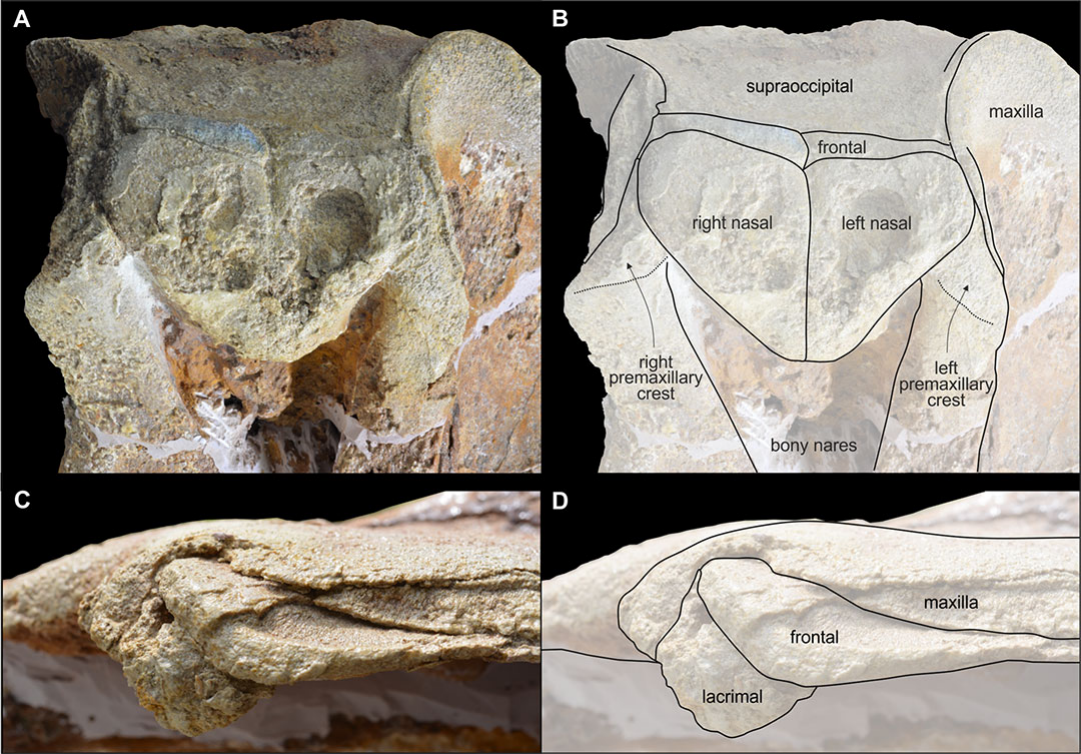
Vertex and antorbital process of *Chimuziphius coloradensis*. Details of the cranium of the holotype (MUSM 2548) of *C. coloradensis*, from the Tortonian of Cerro Colorado (Pisco Basin, Peru): (A) vertex in dorsal view, (B) left antorbital process in lateral view.

**Figure 9:**
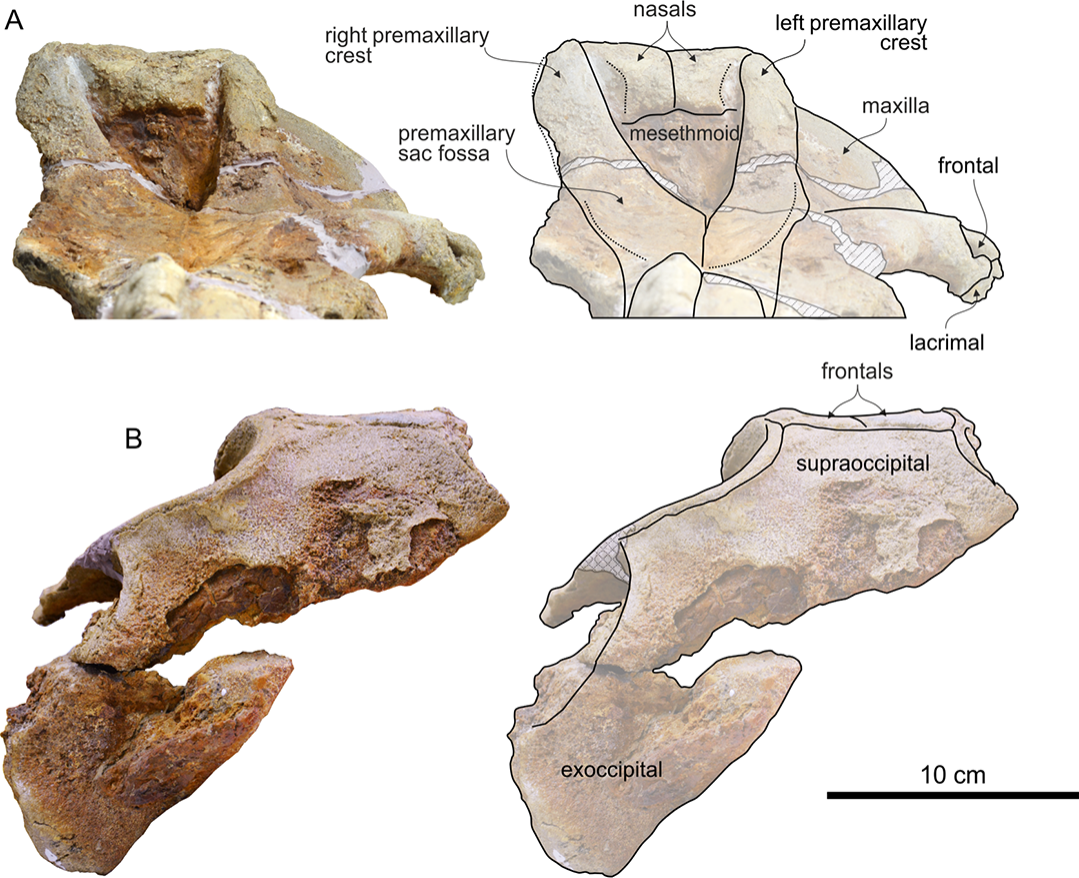
Cranium of *Chimuziphius coloradensis* in anterior and posterior view. Cranium of the holotype (MUSM 2548) of *C. coloradensis*, from the Tortonian of Cerro Colorado (Pisco Basin, Peru) in (A) anterior and (B) posterior view. Linear hatching indicates major breaks, crosshatching indicates reconstructed missing parts.

*Chimuziphius coloradensis*, sp. nov. (Figs. 7–9; Table 2)

**Table 2:** Measurements of the holotype cranium (MUSM 2548) of *Chimuziphius coloradensis*.

	Measurement (mm)
Condylobasal length	580^+^
Length of rostrum	330^+^
Length of neurocranium	230^+^
Width of rostrum base at level antorbital notch	178*
Width of premaxillae at level antorbital notch	82
Preorbital width of skull	250*
Postorbital width of skull	240*
Length of antorbital process of lacrimal	29
Length of orbit	84
Total width of premaxillary sac fossae	111
Maximum width of right premaxillary sac fossa	60
Maximum width of left premaxillary sac fossa	46
Width of bony nares	60
Transverse width across premaxillary crests	125
Width right premaxillary crest	33
Width left premaxillary crest	25
Minimum distance between premaxillary crests	68
Maximum width of nasals	87
Width of right nasal	45
Width of lef nasal	42
Length of medial suture between nasals	53
Minimum posterior distance between maxillae on the vertex	77
Length of medial suture between frontals on the vertex	7

**Notes:**

* indicates doubling of measurement from one side.

+ indicates preserved distance.

**Holotype and only referred specimen:** MUSM 2548, incomplete cranium lacking the anterior portion of the rostrum, most of the right side of the neurocranium, and most of the basicranium.

**Type locality:** Cerro Colorado, Pisco-Ica desert, 35 km southwest of the city of Ica, southern coast of Peru (Fig. 1). Geographic coordinates: 14°21′42.1″S–75°53′07.9″; 580 m above sea level. This specimen was reported in the Cerro Colorado fossil map (Bianucci et al., in press) with the field number O49 and it was provisionally referred to cf. *Nenga* sp.

**Type horizon:** The holotype was found in the lower allomember of the Pisco Formation exposed at Cerro Colorado (Di Celma et al., 2016), 61.5 m above the basal unconformity with the Chilcatay Formation and about four meters below the stratigraphic horizon of the holotype of the giant raptorial sperm whale *Livyatan melvillei* Lambert et al., 2010. First dated to late middle Miocene (Serravallian, 12–13 Ma; Bianucci, Lambert & Post, 2010; Lambert et al., 2010), this basal portion of the Pisco Formation has been recently assigned to late Miocene (Tortonian) based on the occurrence of *Lithodesmium reynoldsii* (a diatom species ranging from 9.9 to 8.9 Ma; Barron, 2003) just a few meters below the holotype of *Chimuziphius coloradensis* (Di Celma et al., 2016). This age is further supported by a radiometric ^40^Ar/^39^Ar dating of biotite from an ash layer ca 30 m below the holotype of *C. coloradensis*, giving an age of 9.10 ± 0.04 Ma (K. Gariboldi et al., 2015, unpublished data). Besides the holotypes of *C. coloradensis* and *Livyatan melvillei*, other fossil marine vertebrates were found in the same lower allomember of the Pisco Formation in Cerro Colorado: other ziphiids (the holotype and most of the referred specimens of *Messapicetus gregarius)*, other physeteroids (e.g., Physeteroidea aff. *Acrophyseter*), pontoporiids (e.g., *Brachydelphis mazeasi* Muizon, 1988), kentriodontid-like delphinidans, mysticetes (cetotheriids and balaenopteroids), crocodiles, sea turtles (the holotype and several referred specimens of *Pacifichelys urbinai* Parham & Pyenson, 2010), seabirds (e.g., the holotypes of *Sula brandi* Stucchi, Varas-Malca & Urbina-Schmitt, 2016 and *S. figueroae* Stucchi, Varas-Malca & Urbina-Schmitt, 2016), sharks (e.g., *Charcharocles* and *Cosmopolitodus*), and bony fish (Bianucci, Lambert & Post, 2010; Bianucci et al., 2016b; Lambert, Bianucci & Post, 2010; Lambert et al., 2010; Parham & Pyenson, 2010; Collareta et al., 2015; Stucchi, Varas-Malca & Urbina-Schmitt, 2016). Many fossil remains of Cerro Colorado (but not the holotype of *C. coloradensis*) are included in dolomite concretions (Gariboldi et al., 2015) leading in some cases to exceptional preservations (Lambert et al., 2015; Gioncada et al., in press).

**Diagnosis:** *Chimuziphius* differs from all other ziphiids in having the following combination of characters: mesorostral groove closed or very narrow due to the medial contact of the premaxillae; moderate thickening of the premaxillae above the mesorostral groove on the rostrum; absence of maxillary crest and rostral maxillary crest; anteroposteriorly elongated premaxillary sac fossa with premaxillary foramen distinctly anterior to the antorbital notch; ascending process of premaxilla gradually rising toward the vertex without reaching the vertical in its posterodorsal portion; absence of a transverse constriction on the ascending process of premaxilla; premaxillary crest anterolaterally directed and with reduced contact with nasal; very large triangular nasals with pointed joined anterior margin, forming an angle of ca 90°; dorsal surface of the nasals with a weak medial depression; weak transverse constriction of the frontals on the vertex; moderate elevation of the vertex of the skull (ratio between the vertical distance from the dorsal margin of the rostrum to the top of the vertex and the width of the premaxillary sac fossae = 0.77); moderate length of the temporal fossa; presence of one large infraorbital foramina on the left maxilla anterior to the base of the rostrum; and thin supraorbital process of frontal. Shares with the *Messapicetus* clade, *Ninoziphius*, and Ziphiinae: premaxillary crest anterolaterally directed and reduced contact of the premaxillary crest with the nasal. Further shares with the *Messapicetus* clade medial contact and moderate thickening of the premaxillae above the mesorostral groove on the rostrum. Differs from *Aporotus*, *Beneziphius*, *Messapicetus*, and *Ziphirostrum* in lacking a conspicuous prenarial basin; from *Beneziphius* and *Choneziphius* in lacking excrescences on the dorsal surface of the maxilla along the posterior half of the rostrum; from *Notoziphius* in having premaxillary crest more laterally directed; nasals with anterior point less ventrally bent and forming a more obtuse angle (90 vs. 75° in the latter); more anteroposteriorly elongated dorsal exposure of the supraoccipital between the maxillae; thinner supraorbital process of the frontal; and in lacking an elliptical fossa in the ascending process of premaxilla and a developed maxillary crest; from *Ninoziphius* in having less elevated vertex and smaller dorsal exposure of the nasals; from Ziphiinae in having shorter nasals; less elevated vertex; less concave dorsal margin of the ascending process of premaxilla in lateral view; from Berardiinae in having the premaxillary foramen distinctly anterior to the antorbital notch; supraoccipital reaching the top of the vertex; less transverse constriction of the frontal on the vertex; and in lacking a nodular protuberance formed by the interparietal or the frontals on the vertex; from *Nenga*, *Pterocetus*, *Xhosacetus*, and the Hyperoodontinae in lacking inclusion of the nasal in the premaxillary crest and from the Hyperoodontinae in lacking a deep anteromedial excavation of the nasals; from *Nazcacetus* and *Tasmacetus* in having less concave dorsal margin of the ascending process of premaxilla in lateral view; premaxillary foramen distinctly anterior to the antorbital notch; and weaker transverse constriction of the frontals on the vertex; from *Chimuziphius* in having nasals more elongated, with a more pronounced medial depression, and more pointed anterior margin; the supraoccipital being more constricted between the maxillae; and in lacking a conspicuous maxillary crest.

**Etymology:** From Cerro Colorado, the vertebrate-rich type locality.

## Description and Comparisons

### Cranium

The cranium (Fig. 7) is smaller than in *Chavinziphius*, having an estimated postorbital width close to *Mesoplodon peruvianus* Reyes, Mead & Waerebeek, 1991 the smallest extant beaked whale (see Bianucci, Post & Lambert, 2008: Table S2). Since the rostrum is anteriorly broken, its original length cannot be measured. Nevertheless, it was probably rather elongated, considering that the preserved portion only gradually tapers towards the preserved distal end. At half the length of the preserved portion, the rostrum is dorsoventrally compressed, wider than high. The vertex is moderately elevated, but less than in *Chavinziphius* and all extant ziphiids (ratio of vertical distance from dorsal margin of the rostrum to top of the vertex to width of the premaxillary sac fossae = 0.77). The anteroposterior extent of the incomplete temporal fossa seems similar to *Chavinziphius*.

#### Premaxilla

On the rostrum the premaxilla is transversely narrow with a weak, gradual widening towards the antorbital notch (Fig. 7A). The medial margins of the right and left premaxillae either contact each other or are very close. Consequently, the dorsal opening of the mesorostral groove is nearly or totally closed. The observed condition could partly result from the diagenetic dorsoventral compression of the cranium, which could also have contributed to some extent to the flattening of the rostrum. In any case, the medial margins of the premaxillae do not seem firmly sutured, contrary to all ziphiids of the *Messapicetus* clade with the exception of *Aporotus*. The rostral portion of the premaxilla anterior to the premaxillary sac fossa exhibits some degree of thickening, particularly in its medial region, where the bone is ca 10 mm thick. A similar but more pronounced thickening is present in all members of the *Messapicetus* clade; the pachyosteosclerotic condition of the premaxilla is particularly developed in *Globicetus* and *Tusciziphius*, both showing a huge premaxillary prominence on the rostrum (Bianucci et al., 2013; Dumont et al., 2016). In part of the members of the *Messapicetus* clade, this premaxillary thickening is followed posteriorly by an excavation of the premaxillae generating a dorsal fossa named prenarial basin; such a feature is absent in *Chimuziphius*. As in *Chavinziphius*, the weakly transversely convex premaxillary sac fossa is anteroposteriorly elongated, extending for 30 mm on the rostrum. A similar elongation of the premaxillary sac fossa is also seen in *Notoziphius*, a ziphiid sharing several features with *Chimuziphius* (see below). Instead, premaxillary sac fossae are significantly shorter in *Nenga*, another fossil ziphiid sharing similarities with *Chavinziphius* at the level of the nasals. Related to this character, *Nenga* exhibits a lateral margin of the premaxillary sac fossa significantly more laterally convex than in *Chimuziphius* and *Notoziphius*. Moreover, the anterior margin of the bony nares formed by the premaxillae, is U-shaped in *Nenga* whereas it is V-shaped in *Chimuziphius* and *Notoziphius*. The degree of asymmetry at the level of the premaxillary sac fossae of *Chimuziphius* is similar to *Notoziphius* (ratio between the widths of the left and right premaxillary sac fossae = 0.85), only slightly smaller than in *Chavinziphius* (0.82) and significantly lower than in *Choneziphius*, *Globicetus*, *Hyperoodon*, *Tusciziphius*, and *Ziphius* (all with a ratio ≤ 0.65). The premaxillary foramen (not clearly visible on both sides, due to the poor preservation) was most likely located near the anterior end of the premaxillary sac fossa, anterior to the level of the antorbital notches.

The rise of the ascending process of the premaxilla is similar to *Berardius*, *Chavinziphius, Ninoziphius*, and *Notoziphius*, generating a moderate concavity of the anterodorsal margin of the neurocranium in lateral view (Fig. 7B). In *Nenga*, a similar concavity is associated to a more abrupt elevation of the premaxilla toward the vertex. In anterior view, the ascending process of the premaxilla does not display an oval-shaped fossa, a feature only described in *Izikoziphius* and *Notoziphius*. The ascending process lacks the transverse constriction (Fig. 9A) as observed in all other ziphiids except *Archaeoziphius*, *Berardius*, *Izikoziphius*, *Microberardius*, *Nenga*, *Notoziphius*, and *Ziphius*. The premaxillary crests are short, thin, and anterolaterally directed, with the right premaxillary crest forming an angle with the sagittal plane (ca 55°) greater than in *Notoziphius* (30–40°)—the latter being characterized by premaxillary crests more anteriorly directed (Fig. 8A). Anterolaterally directed premaxillary crests are also observed in *Messapicetus*, *Ninoziphius*, *Ziphius*, and several related fossil ziphiids. The premaxillary crests are weakly asymmetrical, with the right crest transversely wider and more laterally directed than the left crest (angle with the sagittal plane of ca 40° for the left crest). Differing from *Nenga*, *Notoziphius*, and several other ziphiids, the right premaxillary crest is not higher than the left in anterior view. Related to the shift of the vertex towards the left side in these other taxa, such an asymmetry may have been partly obliterated by the diagenetic deformation of the cranium of the holotype of *Chimuziphius coloradensis*.

#### Maxilla

As in *Ninoziphius*, the maxilla of *Chimuziphius* does neither present rostral maxillary crests nor maxillary crests at the base of the rostrum and in the anterior part of the neurocranium; consequently, the dorsal surface of the posterior rostral portion of the maxilla and the supraorbital portion of the maxilla is flat (Fig. 7A). Most other ziphiids have well distinct maxillary crests, although these crests are relatively low in *Nazcacetus* and *Notoziphius*. The dorsal surface of the left maxilla at the posterior end of the rostrum is pierced by a single large infraorbital foramen, opening anterodorsally. This foramen is located 45 mm anterior to the antorbital notch and its transverse width is 9 mm. Two other, smaller infraorbital foramina pierce the left antorbital process, 12 and 42 mm posterior to the level of the antorbital notch. The palatal surface of the maxilla is poorly preserved; the presence or absence of maxillary alveoli thus cannot be assessed.

#### Vomer

Due to the very narrow dorsal opening of the mesorostral groove, the vomer is not exposed dorsally (Fig. 7A). Nevertheless, there is no evidence for the presence of a filling of the groove by a pachyosteosclerotic vomer; this condition contrasts with adult males of *Mesoplodon*, *Ziphius* and related fossil taxa.

#### Nasal

The joined very large nasals have a triangular dorsal outline, more anteroposteriorly elongated than in *Chavinziphius* (Fig. 8A). Similarly sized nasals are observed in *Nenga* and *Notoziphius*. The unexcavated anterior margin exhibits an anterior point making an angle of ca 90°, slightly more obtuse than in the holotype of *Notoziphius bruneti* (75°). The lateral margin of the nasal only contacts the premaxilla for a short distance posteriorly, as in *Notoziphius*, but differing from *Chavinziphius* and *Nenga* (both displaying an extensive contact between nasal and premaxilla). The dorsal surface of the nasals is almost flat, with only a shallow medial concavity (Fig. 9B); this surface slopes slightly anteroventrally, but less than in *Notoziphius*. The sutures between nasals and frontals are slightly anteriorly pointed, not to the extent of *Notoziphius*.

#### Frontal

Although the orbit displays an anteroposterior length proportionally similar to *Chavinziphius*, its roof is significantly thinner (Fig. 8B). The frontal is actually dorsoventrally thin not only on the orbit, but also in the antorbital region, a condition also observed in *Nazcacetus*. On the vertex, the exposure of the frontals between the nasals and the supraoccipital is much reduced, as in most other ziphiids (Fig. 8A). The transverse compression of the frontals between the maxillae on the vertex is less developed than in all other ziphiids except *Messapicetus* and *Notoziphius*.

#### Supraoccipital and exoccipital

As in *Chavinziphius*, the ascending process of the maxilla extends for a long distance posterior to the straight, transversely directed anteromedial suture between the frontals and the supraoccipital (Fig. 7A). Consequently the supraoccipital displays an anteroposteriorly elongated dorsal exposure, which is nevertheless transversely narrower than in *Chavinziphius*. For this character *Chimuziphius* differs from *Notoziphius*, which has a supraoccipital shield that drops abruptly posteroventrally, with a consequently shorter dorsal exposure. As in all ziphiids except *Archaeoziphius* and *Berardius*, the anteromedial margin of the supraoccipital reaches the same level as the top of the vertex.

#### Exoccipital and squamosal

Only the incomplete left exoccipital is preserved. Its paroccipital process is sutured with an eroded and uninformative fragment of squamosal (Figs. 7B and 9B).

#### Palatine and pterygoid

On the worn ventral surface of the skull, the area tentatively interpreted as the pterygoid-palatine suture is marked by an arched bulge extending ca 90 mm anterior to the antorbital notch (Fig. 7B). This bulge limits anterolaterally a large depression, corresponding to the vast pterygoid sinus fossa typical for all ziphiids.

#### Lacrimal

The well-preserved left lacrimal is clearly visible in lateral view, displaying a peculiar drop shape (Fig. 7B). This bone is dorsally wedged between the maxilla and the frontal.

**Figure 10:**
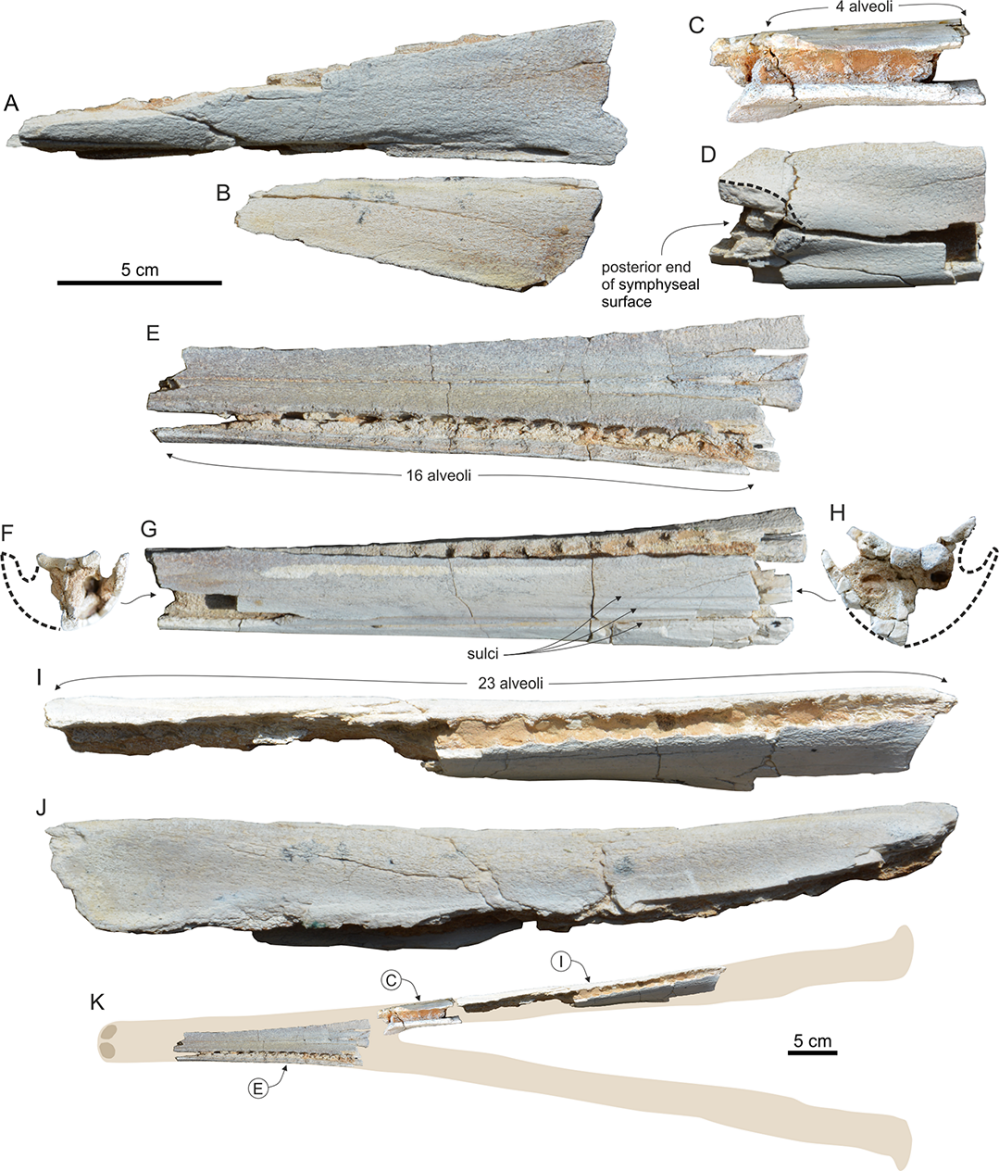
Fragmentary cranium and mandibles of Genus and sp. indet. 1. Fragmentary skull and mandibles of Genus and sp. indet. 1 (MUSM 3237), from the Tortonian of Cerro Los Quesos (Pisco Basin, Peru): (A) rostral portion of left maxilla in ventral view; (B) rostral portion of right maxilla in ventral view; small fragment of right mandible in (C) dorsal and (D) medial view; incomplete symphyseal portion of fused mandibles in (E) dorsal, (F) anterior, (G) left lateral, and (H) posterior view; fragment of the postsymphyseal portion of the right mandible in (I) dorsal and (J) lateral view; (K) assembled fragments of the mandibles in dorsal view over reconstructed silhouettes of the complete mandibles.

**Figure 11:**
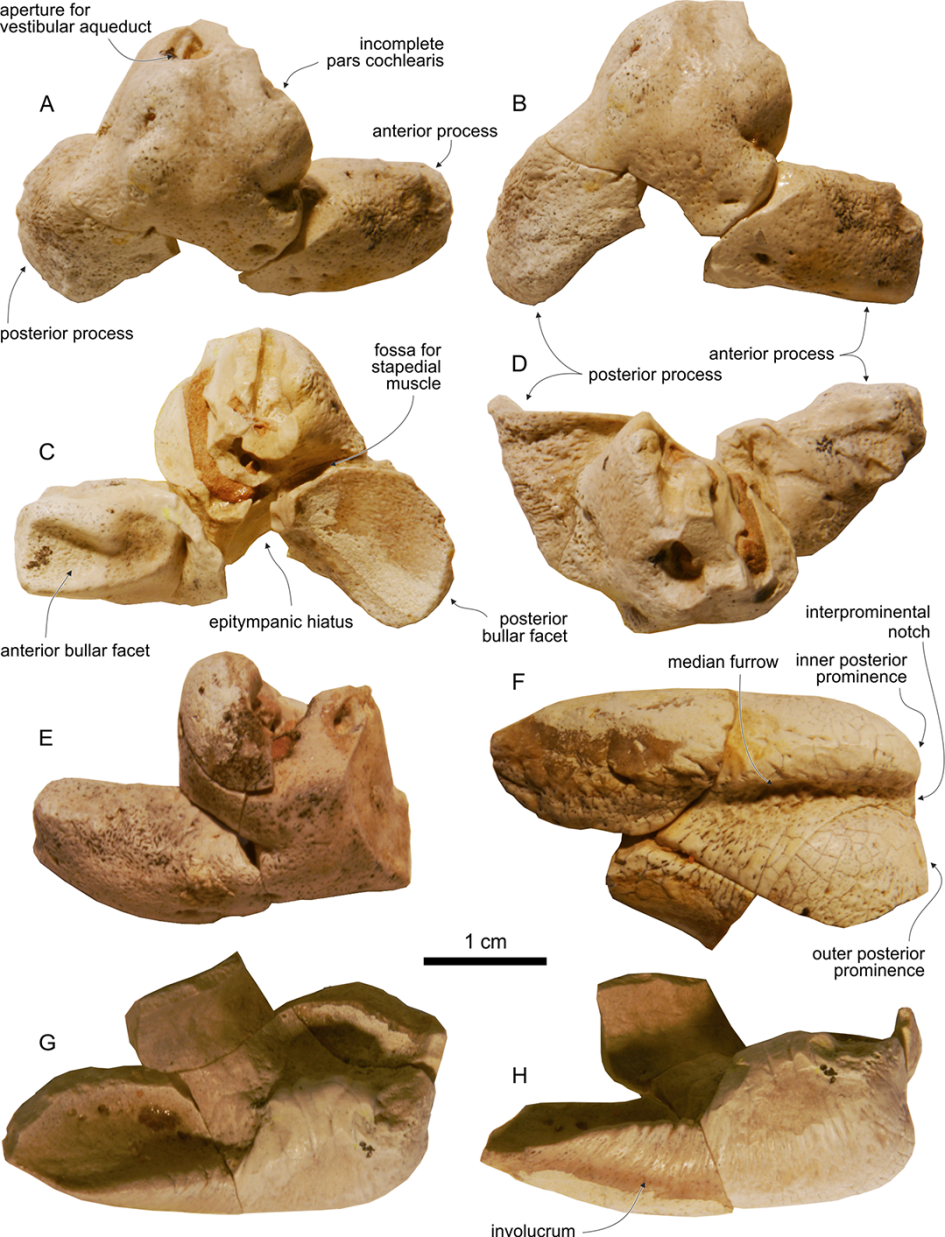
Periotics and tympanic bulla of Genus and sp. indet. 1. Periotics and tympanic bulla of Genus and sp. indet. 1 (MUSM 3237), from the Tortonian of Cerro Los Quesos (Pisco Basin, Peru): right periotic in (A) dorsal, (B) dorsomedial, (C) ventral, and (D) medial views; (E) fragmentary left periotic in dorsal view; right tympanic bulla in (F) ventral, (G) dorsal, and (H) medial view.

Genus and sp. indet. 1 (Figs. 10 and 11)

**Referred specimen:** MUSM 3237, a fragmentary skull consisting of two rostral portions, of right and left maxilla, respectively; three mandibular fragments (incomplete symphyseal portion including fused fragments of right and left mandibles and two fragments of the postsymphyseal portion of the right mandible); incomplete right and left periotics and tympanic bullae, partly reconstructed by reassembling small detached fragments found on the ground. Other more damaged skeletal remains of the same individual, both from the cranium and the mandibles, are still in the field.

**Locality:** Cerro Los Quesos, 2.4 km southeast from the top of the main hill (Fig. 1). Geographic coordinates: 14°31′36.75″S–75°44′12.95″W; 620 m above sea level. This specimen was not reported in the map showing the distribution of vertebrate fossils at Cerro Los Quesos (Bianucci et al., in press), since it has been discovered in February 2016, after its publication.

**Horizon:** MUSM 3237 was found near the base of the Member A of the Pisco Formation as defined by Di Celma et al. (in press), ca 90 m below a volcanic ash layer that was dated to 7.55 ± 0.05 Ma based on ^40^Ar/^39^Ar radiometric analyses, and ca 40 m below the first occurrence of the diatom species *Thalassisora antiqua*, dated to 8.5 Ma based on the low-latitude diatom zonation of Barron (1985) (Di Celma et al., in press). Since the horizon where MUSM 3237 was found is just above the unconformity between the lower and upper allomembers recorded at Cerro Colorado, MUSM 3237 cannot have an age older than 9 Ma, which is the age of the lower allomember of Cerro Colorado based on both diatoms and ^40^Ar/^39^Ar radiometric dating. As a consequence, the age of MUSM 3237 is constrained to an interval between ca 9 and 8.5 Ma (Tortonian, late Miocene).

## Description and Comparison

### Cranium

The cranium was almost completely destroyed by recent erosion and only two small fragments of maxillae have been collected. These right and left palatal processes of maxillae are 110 and 190 mm long, respectively. Their lateral margins are broken and consequently the alveoli are nearly completely lost, apart from traces of their medial margin, still visible along the broken lateral margin of the fragment of right maxilla: 11 alveoli are counted on a length of 110 mm, confirming that the size of the alveoli in the rostrum was similar to that in the mandible (see below).

### Tympanic bulla

The tympanic bulla is close in general shape to *Messapicetus* and *Ninoziphius*, having an inner posterior prominence that is much transversely narrower and posteriorly shorter than the outer posterior prominence in ventral view, and having a deep medial furrow separating both prominences (Figs. 11F–11H). In medial and dorsal views the dorsal margin of the involucrum exhibits a marked indentation, as in *Ninoziphius* and most other ziphiids, except *Messapicetus*—the latter being characterized by a weaker indentation.

### Periotic

As for the tympanic bulla, the periotic is substantially similar to the periotic of *Messapicetus* and *Ninoziphius*, showing a primitively slender anterior process, less mediolaterally thickened than in *Nazcacetus* and all extant ziphiids (Figs. 11A–11E). The maximum length of the tympanic is 37 mm; it is slightly smaller than in *Messapicetus gregarius* (41.5 mm in the referred specimen MUSM 950) and *Ninoziphius platyrostris* (42.0 mm in the holotype). The anterior bullar facet is deep and the fovea epitubaria is ventrally exposed in both periotics, since the accessory ossicle is missing. The pars cochlearis is spherical and anteriorly projected, as in all other ziphiids. Due to the very fragmentary preservation of the pars cochlearis in both periotics, the presence of a large cochlear spine (perhaps the only distinctive feature separating the periotic of the holotype of *Ninoziphius platyrostris* from periotics of *Messapicetus*) cannot be evaluated. In ventral view, the posterior bullar facet is fan-shaped and longitudinally concave, with the posterior margin being more rounded than in *Messapicetus* and *Ninoziphius*.

### Mandible

The ankylosed fragment of the symphyseal portion of the mandibles is 203 mm long and its transverse section is semicircular (Figs. 10E–10H), as in *Berardius*, *Messapicetus*, *Ninoziphius*, and *Tasmacetus*. The dorsal surface of this portion is transversely concave (a feature more pronounced posteriorly) and its lateral surface is cut by three parallel longitudinal sulci that were probably followed posteriorly by small mental foramina on the missing portion of the mandible. On the best-preserved left side 16 well-defined circular alveoli are observed. Their diameter increases gradually posteriorly (from 8 to 10 mm). The smallest fragment of the postsymphyseal portion of the right mandible (anteroposterior length 85 mm) preserves five large circular alveoli (diameter 11 mm); the posterior end of the symphysis is visible along the medial surface of this fragment (Figs. 10C and 10D). At the level of the posterior end of the symphysis, the height and width of the mandible are 45 and 25 mm, respectively. The other fragment of the right mandible (anteroposterior length 280 mm) preserves 13 complete alveoli (including the posteriormost alveolus of the alveolar groove) with diameters varying from 9 to 7 mm, and traces of 10 more alveoli (Figs. 10I and 10J). Based on these fragments, a lower tooth count greater than 44 can be proposed with the postsymphyseal portion bearing at least 28 alveoli. The lower alveoli of *Ninoziphius* are similar in shape and size to MUSM 3237, but the tooth count of the holotype of the latter is lower (40–42; Muizon, 1984; Lambert, de Muizon & Bianucci, 2013), with the postsymphyseal portion only bearing 20 alveoli.

On the whole, the three fragments of MUSM 3237 show the highest degree of similarity with mandibles characterized by an elongated symphyseal portion (Fig. 10K) and associated to a narrow and elongated rostrum, such as in *Messapicetus* and *Ninoziphius*.

## Remarks

This fragmentary specimen could be unequivocally referred to a ziphiid, possessing two synapomorphies only observed in this family: 1) transverse thickening of the anterior process of the periotic (char. 21 of the phylogeny) and 2) fan-shaped posterior bullar facet of the periotic (char. 20). Moreover, the dorsal margin of the involucrum of the tympanic bulla of MUSM 3237 is cut by a deep indentation clearly visible in dorsal view (char. 24), a feature observed in most ziphiids. However, such an indentation is also present in the Eurhinodelphinidae and some other archaic odontocetes, although being more distinct in medial view (Lambert, de Muizon & Bianucci, 2013). The periotic and the tympanic bulla suggest close affinities with *Messapicetus* and *Ninoziphius*, although these affinities are predominantly based on plesiomorphic features. The complete, functional dentition and the fact that the rostrum was probably elongated and narrow are additional primitive characters shared with *Messapicetus* and *Ninoziphius*, but also with the extant *Tasmacetus*. The circular shape of the alveoli and the greater mandibular tooth count exclude an attribution of MUSM 3237 to *Messapicetus* or *Notoziphius*—the latter being another tooth-bearing ziphiid with large and elongated alveoli, but probably with a lower tooth count (Buono & Cozzuol, 2013). Although the alveoli of MUSM 3237 are similar in size and shape to *Ninoziphius*, the higher tooth count (particularly for the postsymphyseal portion) prevents from a referral to *Ninoziphius*. The latter being found in considerably younger strata of the Pisco Fm., dated to 3.9–5.93 Ma (Lambert, de Muizon & Bianucci, 2013), the affinities between MUSM 3237 and *Ninoziphius* may simply be due to a basal position among ziphiids. Compared with the other fossil beaked whales from the Pisco Fm, MUSM 3237 clearly differs from *Chavinziphius* and *Nazcacetus* in the symphyseal portions of the mandibles being ankylosed and with a semicircular transverse section; it further differs from the smaller *Nazcacetus* in having distinct alveoli, more primitive ear bones and, probably, a more elongated rostrum; it differs from *Chavinziphius* and MUSM 1609 (see below) in having deeper and larger alveoli. Finally, a comparison with *Chimuziphius* (only known on the basis of an incomplete skull lacking ear bones and mandibles) is not possible, since the preserved portions of the rostrum of MUSM 3237 are too fragmentary. In conclusion, pending the discovery of a more complete specimen, we prefer not to provide a more precise systematic attribution for this fragmentary specimen.

**Figure 12:**
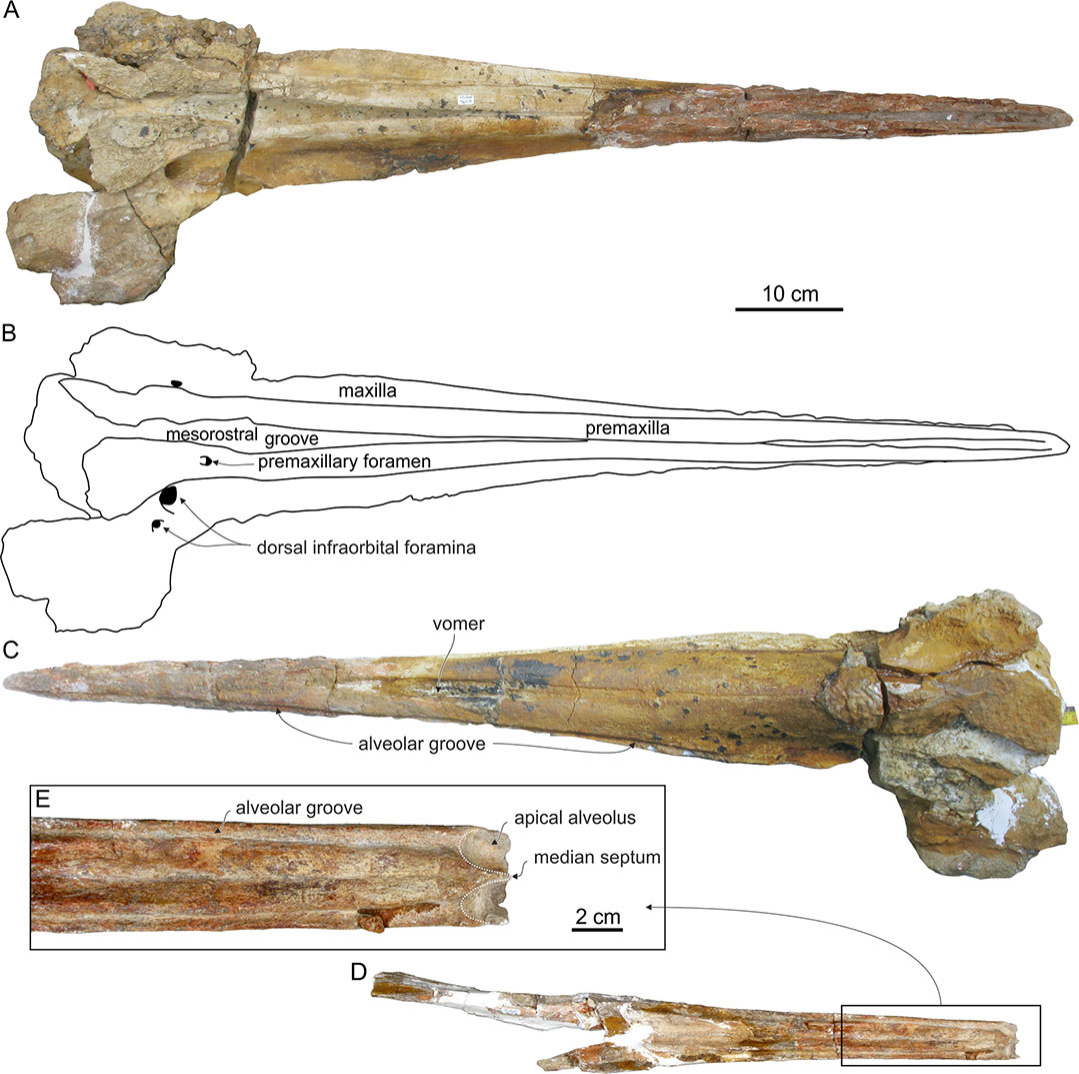
Rostrum and mandibles of Genus and sp. indet. 2. Rostrum and fragmentary mandibles of Genus and sp. indet. 2. (MUSM 1609), from the Messinian of Cerro Los Quesos (Pisco Basin, Peru): rostrum in (A) dorsal and (B) ventral view; mandibles in dorsal view (C) and detail of the apical portion (D).

Genus and sp. indet. 2 (Fig. 12; Table 3)

**Table 3:** Comparison between the measurements and related ratios of Ziphiidae Genus and sp. indet. 2 (MUSM 1609) with the holotype (MUSM 1037) and referred specimens (MUSM 1038) of *Messapicetus gregarius*, and with the holotype (MNHN SAS941) of *Ninoziphius platyrostris*.

	MUSM 1609	MUSM 1037	MUSM 1038	MNHN SAS941
Length of rostrum (A)	798	844	795	685*
Width of rostrum base at level antorbital notch (B)	214	217	208	–
Width of rostrum at midlength (C)	57	69	64	–
B/A	0.27	0.26	0.26	–
C/A	0.07	0.08	0.08	–
Length of symphyseal portion of mandible (D)	330^+^	385	390	310
D/A	0.41	0.46	0.49	0.45

**Notes:**

* indicates estimated measurement.

+ indicates preserved distance.

**Referred specimen:** MUSM 1609, a rostrum with the anterior part of the facial area of the cranium and associated incomplete mandibles.

**Locality:** Cerro Los Quesos, 2.4 km southeast from the top of the hill (Fig. 1). Geographic coordinates: 14°31′06.95″S–75°43′01.75″W; 695 m above sea level. This specimen was reported in the Cerro Los Quesos fossil map (Bianucci et al., in press) with the field number O16; it was provisionally referred to “Ziphiidae n.gen.1 n.sp.”

**Horizon:** MUSM 1609 was found in the Member F of the Pisco Formation as defined by Di Celma et al. (in press), about 9 m above the level of the holotype of *Chavinziphius maxillocristatus*. As for the latter specimen, the horizon of MUSM 1609 is dated between 6.93 ± 0.09 and 6.71 ± 0.02 Ma (Messinian, latest Miocene).

## Description and Comparison

### Cranium

The complete rostrum is morphologically close to the longirostrine ziphiid *Messapicetus gregarius* from Cerro Colorado, for both its size and outline in dorsal and ventral view (Figs. 12A and 12B). As in *M. gregarius*, the rostrum of MUSM 1609 is extremely elongated and narrow, showing similar ratios (1) between the width of the rostrum at its base and the rostrum length, and (2) between the width of the rostrum at mid-length and the rostrum length (Table 3).

#### Premaxilla

The anterior half of the dorsal portion of the rostrum was damaged by Recent erosion; consequently the anterior portion of the premaxillae is poorly preserved (Fig. 12A). Nevertheless, the anterior premaxillary portion of the rostrum is about 30 mm long and, although narrow, a dorsal opening of the mesorostral groove extends for about 300 mm posteriorly from the apex of the rostrum; posteriorly, the medial margin of the right and left premaxillae contact or nearly do so for about 250 mm, before a slight divergence of the premaxillae, with a maximum separation of 45 mm at a level 70 mm anterior to the base of the rostrum. An extended dorsomedial contact of the premaxillae along the central portion of the rostrum is similarly observed in *Messapicetus* and related ziphiids. Nevertheless, unlike in *Messapicetus* the premaxillae of MUSM 1609 are not dorsomedially sutured and are not as thickened as in *Messapicetus* and related taxa (all included below in the *Messapicetus* clade), actually even less thickened than in *Chimuziphius coloradensis*. Another clear difference between MUSM 1609 and *Messapicetus* is the absence of a prenarial basin in the former; indeed, in the posterior portion of the rostrum of the former, the premaxillae are not abruptly excavated, differing from *Messapicetus* and some related taxa. In MUSM 1609, the maximum dorsomedial separation of the two premaxillae occurs in the same area as the prenarial basin of *Messapicetus*; this separation may have had an analogue, echolocation-related function. The premaxillary foramen is 20 mm anterior to the level of the antorbital notch, a condition shared with, among others, *Messapicetus* and the other fossil ziphiids possessing a prenarial basin. The preserved portions of the premaxillae on the neurocranium are partly worn and the apparent marked asymmetry of the premaxillary sac fossae could be an artifact due to the less complete preservation state of the left fossa.

#### Maxilla

On the rostrum, the maxilla is dorsally exposed lateral to the premaxilla for about 500 mm, with the lateral margin making an acute crest for a great extent (about half rostrum length) (Fig. 12A); this crest is more pronounced than in *Messapicetus gregarius*. The dorsal surface of the maxilla is wide and slightly transversely concave at the base of the rostrum. At the level of the antorbital notch, a large dorsal infraorbital foramen is present on the right side (transverse diameter = 21 mm). A second, smaller foramen is lateral to the main foramen. The palatal surface of the maxilla shows a distinct alveolar groove, but lacking well-defined alveoli—a difference with *Messapicetus*, *Ninoziphius*, and *Tasmacetus*. Ventromedially, the vomer is exposed for more than 300 mm. The posterior portion of the ventral surface of the maxillae, including the suture with the palatines, is obscured by a dolomite incrustation.

### Mandible

Only the anterior portion of the ankylosed mandibles, including most of their symphyseal portion (only lacking a few millimeters at the apex), is preserved (Fig. 12C). The dorsomedial surface is distinctly transversely concave. The transverse section is semicircular as in *Berardius*, *Messapicetus*, *Ninoziphius*, and *Tasmacetus*. Although it was probably slightly shorter than in *Messapicetus gregarius* and *Ninoziphius platyrostris*, the symphyseal portion is relatively elongated compared to the rostrum length (Table 3). As for the upper alveolar groove, the lower alveolar groove is narrow and lacks distinct alveoli, except for one large apical pair. Although lacking the anterior margins, the apical alveoli are clearly transversely compressed as in *Messapicetus*, whereas *Ninoziphius* is characterized by subcircular aveoli (Fig. 12D). The transverse diameter of the left apical alveolus is 14 mm. The medial septum separating right and left apical alveoli is thin and barely anteriorly prominent, less than in some mandibles of *Messapicetus gregarius* interpreted as belonging to adult males (Lambert, Bianucci & Post, 2010; Bianucci, Lambert & Post, 2010).

## Remarks

As a result of the preservation of the anterior portion of the mandibles bearing a pair of enlarged apical alveoli for anterior tusks (char. 28, state 2), this fragmentary specimen could be confidently assigned to a ziphiid. As mentioned above, size and outline in dorsal and ventral view of the rostrum are similar to *Messapicetus gregarius*. Nevertheless, MUSM 1609 clearly differs from *Messapicetus* in lacking: a strong thickening of the premaxillae on the rostrum, a medial suture between the premaxillae on the rostrum, the prenarial basin, and large and distinct postapical alveoli. MUSM 1609 differs from *Ninoziphius* (the other fossil ziphiid with a similar elongation of the rostrum) in: having a narrower rostrum; having smaller and indistinct alveoli; and lacking slightly larger, well defined subapical alveoli on the mandible. MUSM 1609 differs from *Chavinziphius* and *Nazcacetus* in having the symphyseal portions of the mandibles ankylosed and displaying a semicircular transverse section; it further differs from *Chavinziphius* in lacking a robust and elevated longitudinal rostral maxillary crest; it further differs from *Nazcacetus* in having a more elongated rostrum and a larger size; it differs from *Chimuziphius* in: having a transverse widening of the mesorostral groove near the base of the rostrum, the largest dorsal infraorbital foramen on the maxilla being more posteriorly located, and having a larger size. Although the preserved rostrum and mandibles could be sufficient for assigning MUSM 1609 to a new genus (probably a stem ziphiid closely related to *Chimuziphius* and the *Messapicetus* clade; see the phylogeny paragraph below), we prefer to maintain an open classification for this specimen since other diagnostic parts of the skull (e.g., the vertex) are not preserved. This approach is consistent with the one followed in recent papers dealing with the description of fragmentary ziphiid remains (Bianucci, Lambert & Post, 2007; Bianucci et al., 2013).

## Phylogenetic Analysis

The phylogenetic relationships of *Chavinziphius* and *Chimuziphius* with the other Ziphiidae are investigated here using the same methods and the same matrix as Lambert, de Muizon & Bianucci (2013), with only a few additions and minor changes as reported below.

Besides the newly diagnosed *Chavinziphius* and *Chimuziphius* from the late Miocene of Peru, the following taxa are added:

– *Aporotus dicyrtus* and *A. recurvirostris* du Bus, 1868, both from the Neogene of the North Sea Basin (Lambert, 2005), allowing a better definition of the “*Messapicetus* clade”(MC hereafter); the two species of this genus are included separately in the analysis, since their skulls differ significantly;
– *Nenga*, from the Neogene of South Africa (Bianucci, Lambert & Post, 2007), sharing some cranial similarities with *Chimuziphius* (especially the large nasals);
– *Notoziphius*, from the late Miocene of Argentina (Buono & Cozzuol, 2013), close to *Chimuziphius* for several features of the skull; not included in the phylogeny of Lambert, de Muizon & Bianucci (2013) since its description was published later; note that characters 7 and 16 are coded here differently from the matrix in Buono & Cozzuol (2013).

Five new characters (chars. 47–51) are added and they concern:

– (char. 47) the anteroposterior length of the temporal fossa (character modified from Lambert, de Muizon & Bianucci (2015)); the shortening of the fossa is thought to be linked to the suction feeding specialization of more derived species;
– (char. 48) the number and size of the dorsal infraorbital foramina on the maxilla near the base of the rostrum; most adults of extant ziphiid species only have a large foramen, sometimes associated to a few, significantly smaller foramina; a cluster of smaller foramina could represent the primitive state, observed in immature specimens of extant ziphiids (see File S2) and in several fossil species;
– (char. 49) the presence of excrescences on the dorsal surface of the maxilla along the posterior half of the rostrum; this is a derived character only observed in *Beneziphius* and *Choneziphius*;
– (char. 50) the posterior narrowing of the nasals and frontals on the vertex; it is present in *Archaeoziphius, Berardius, Tasmacetus*, and, less marked, in *Nazcacetus;*
– (char. 51) the degree of fusion of the cervical vertebrae in adult specimens, a feature discussed in Lambert et al. (2015).

The following characters were modified:

– (char. 3) an additional state was added to distinguish the taxa having premaxillae unfused but in tight dorsomedial contact for a long distance along the rostrum; this intermediate condition was observed in the outgroup eurhinodelphinids, *Aporotus* and possibly *Chimuziphius*;
– (char. 27) the observation of the presence/absence of functional teeth is here not restricted to the maxilla, but also applied to the mandibles, in order to be able to code *Chavinziphius*, in which the palatal surface of the maxilla is obscured (alveoli unrecognizable), whereas mandibular alveoli are well preserved;
– (char. 30) this character was reformulated, considering the premaxillary prominence/bulge observed in *Globicetus* and *Tusciziphius* as an overgrowth of the pachyosteosclerotic condition of the premaxillae observed in all taxa of the MC as redefined here.

After these changes, the matrix includes 34 taxa, of which 28 belong to the family Ziphiidae, coded for 51 morphological characters. Twenty-eight characters are binary, 19 are multistate and ordered, and four are multistate and unordered. Multistate characters were treated as ordered when character states could be arranged so that each state was most similar to the states adjacent to it (e.g., state 1 is more similar to states 0 and 2 than states 0 and 2 are similar to each other) (Geisler & Sanders, 2003; Bianucci, Lambert & Post, 2010).

The analysis was executed with the software PAUP (v. 4.0b10, Swofford, 2001), using the branch-and-bound algorithm. The characters were analyzed under both equal and implied weight.

**Figure 13:**
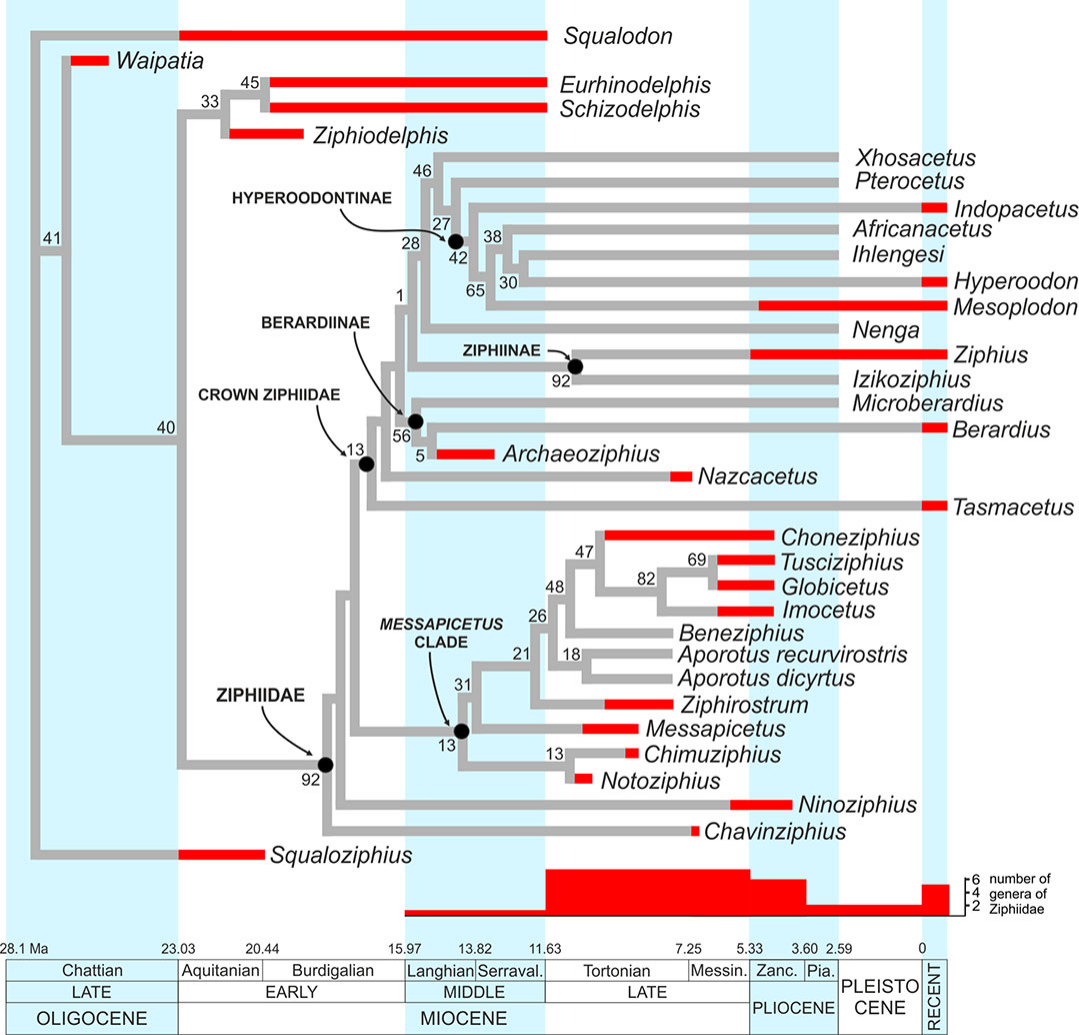
Stratigraphically calibrated phylogenetic tree of Ziphiidae. Single most parsimonious, stratigraphically calibrated tree resulting from the cladistic analysis of 51 morphological characters for 28 ziphiids and six outgroups. Homoplastic characters downweighted using the method of Goloboff (1993).Tree length = 167, Goloboff fit = −37.87, ensemble consistency index = 0.50, and ensemble retention index = 0.77. Numbers associated with the branches are GC values with 100,000 replicates (only values > 0 are shown). See text for discussion and Supplemental Information (File S1) for description of characters and data matrix. Calibration for major nodes and stratigraphic ranges are according to the data reported, respectively in Tables 4 and 5. Chronostratigraphic scale follows Cohen et al. (2013).

The equally weighted analysis generated nineteen equally most parsimonious cladograms (MPCs hereafter). The consensus tree of these MPCs (see File S1) evidences several unresolved relationships within the crown Ziphiidae (CZ hereafter). The results of the implied weighting analysis are: three equally MPCs for K = 1; nine equally MPCs for K = 2; one and the same MPC for K = 3–15; and seven equally MPCs for K = 16–1,405 (see File S1). To select the best parsimonious cladogram, we compared for each MPC the summed Group present/Contradict (GC hereafter) values of all nodes using the software TNT (v. 1.1, Goloboff, Farris & Nixon, 2008), following the same method as in Harbach & Kitching (2016). We preferred GC values to compare the MPCs due to problems arising when applying other more traditional support methods (e.g., bootstrap) to weighted data (Goloboff et al., 2003; Harbach & Kitching, 2016). The comparison of the MPCs with different K values does not reveal significant changes of the GC values for K = 1–15, and only a weak decrease (and lower support) of GC value for K > 15. Although all the obtained MPCs display a substantially similar topology, we choose the better resolved MPC with K = 3–15 (a single cladogram). More specifically, we restrict the K value to 3, which is the one providing the highest Goloboff fit value; K = 3 is also the value used in previous ziphiid analyses (Bianucci, Lambert & Post, 2007; Bianucci, Lambert & Post, 2010; Bianucci et al., 2013; Buono & Cozzuol, 2013; Lambert, de Muizon & Bianucci, 2013). The chosen single MPC has tree length = 167, Goloboff fit = −37.87, ensemble consistency index = 0.50, and ensemble retention index = 0.77. The cladogram and the GC support values are presented in Fig. 13 and are discussed below.

The MPC obtained in our analysis is substantially similar to the 45 equally MPCs obtained by Lambert, de Muizon & Bianucci (2013). The only major difference consists in the shift of the clade formed by *Choneziphius*, *Globicetus*, *Imocetus*, and *Tusciziphius* inside the MC (now also including *Chimuziphius* and *Notoziphius*). In fact, members of the former clade were considered by Bianucci et al. (2013), Lambert, de Muizon & Bianucci (2013) and Buono & Cozzuol (2013) inside the subfamily Ziphiinae, together with *Izikoziphius* and *Ziphius*. It is important to outline that this major difference with respect to previously published analyses is also found in the MPCs obtained with different K values and in an equally-weighted analysis (see File S1). The new placement of *Choneziphius*, *Globicetus*, *Imocetus*, and *Tusciziphius* is supported by the peculiar morphology of the premaxillae on the rostrum, shared by these four genera with *Aporotus*, *Beneziphius*, *Messapicetus*, and *Ziphirostrum*: only these genera exhibit a marked pachyosteosclerotic development of the premaxillae (char. 30, states 2–3), with the dorsomedial margins fused for an extended length (except in *Aporotus*, where a tight, unfused contact is observed), dorsally closing the mesorostral groove (char. 3, states 2–4). Further supporting this new combination of ziphiid taxa, the presence of excrescences on the dorsal surface of the maxilla on the posterior half of the rostrum (char. 49) is present in *Choneziphius*, *Beneziphius* (see Lambert, 2005; Bianucci et al., 2013), and a rostrum from the seafloor off Galicia referred to aff. *Ziphirostrum* sp. by Bianucci et al. (2013). Most likely related to the attachment of facial muscles, these peculiar excrescences are nevertheless subject to intraspecific variation (Lambert, 2005; Bianucci et al., 2013) and were for now not yet observed in other members of the clade. Finally, the paleobiogeographical distribution of fossil ziphiids (see below) further supports the newly proposed placement of *Choneziphius* inside the MC. Our new analysis also confirms: (1) the monophyly of *Aporotus*, since *A. dicyrtus* and *A. recurvirostris* form a clade (although with a low GC support value) and (2) the inclusion of *Aporotus* in the MC, as already proposed by Buono & Cozzuol (2013).

The referral of *Chavinziphius* and *Chimuziphius* to the family Ziphiidae is supported by (1) the presence of premaxillary crests (char. 32, state 1; also present in *Squaloziphius* and, to a lesser extent, some eurhinodelphinids and delphinidans); (2) the moderately elevated vertex (char. 9, state 1); and (3) the wide hamular fossa of the pterygoid sinus extending anteriorly on the palatal surface of the rostrum (char. 35, state 1). Moreover, in *Chavinziphius* the basicranium and the mandible (unknown in *Chimuziphius*) exhibit three additional ziphiid characters: (1) anteroposterior shortening of the zygomatic process of the squamosal (char. 38, state 1); (2) ventral margin of the postglenoid process of the squamosal clearly more dorsal than the ventral margin of the paroccipital process of the exoccipital in lateral view (char. 39, state 2); and (3) presence of a precoronoid crest on the dorsal margin of the mandible (char. 44, state 1).

According to our phylogenetic analysis, *Chavinziphius* is the earliest diverging ziphiid. Its basal position is not due to the lack of some of the main ziphiid synapomorphies (see above), but rather to the absence of features distinguishing other more derived beaked whale clades: e.g., the fused pachyosteosclerotic premaxillae on the rostrum and the presence of a prenarial basin in the MC, the nodular protuberance formed by the interparietal or the frontals on the vertex in the Berardiinae, and the deep anteromedial excavation of the nasals in the Hyperoodontinae.

Although being also considered as a stem ziphiid, *Chimuziphius* branches after *Chavinziphius*: with *Notoziphius* it forms a clade (GC = 13) that is in a basal position within the MC, sharing with the other members of the MC two clearly derived features: closed mesorostral groove due to the dorsomedial contact of the premaxillae (char. 3, state 1); and weak pachyosteosclerotic development of the premaxillae on the rostrum (char. 30, state 1). Among all ziphiids, these characters are only observed in *Chimuziphius* and all taxa of the MC, supporting the hypothesis that they are exclusive features of a large clade, of which *Chimuziphius* and *Notoziphius* are the earliest diverging members. Unfortunately these two characters cannot be coded in *Notoziphius*, since the rostral portion of the premaxillae of the only specimen referred to this genus is poorly preserved. Since *Chimuziphius* and *Notoziphius* are sister taxa, *Chimuziphius coloradensis* could have been referred to the same genus as *Notoziphius bruneti*. However, we prefer to assign the Peruvian species to a new genus, considering that: 1) no unambiguous synapomorphy defines the node generating the *Notoziphius* + *Chimuziphius* clade; 2) there are marked differences between the holotype skulls of the two species, as outlined in the diagnosis and in the comparative description of *C. coloradensis*. As for *Nenga*, the other ziphiid for which rough similarities were noted with *C. coloradensis*, this South African taxon falls in a more derived position than *Chimuziphius*, the same as already proposed in Buono & Cozzuol (2013); *Nenga* is placed within the CZ, sister-group to the large clade formed by Hyperoodontinae + *Pterocetus* + *Xhosacetus*, and sharing with these taxa one synapomorphy: the inclusion of the nasal in the premaxillary crest (char. 15). Although the too fragmentarily known specimen MUSM 1609 referred to “Ziphiidae Gen. et sp. indet. 2” was not inserted in the phylogenetic analysis, it is reasonable to propose a basal position within the MC, as for *Chimuziphius;* in fact MUSM 1609 shares with *Chimuziphius* a closed mesorostral groove and the weak pachyosteosclerotic development of the premaxillae on the rostrum. The other fragmentary specimen MUSM 3237, referred to “Ziphiidae Gen. et sp. indet. 1,” could be placed in a similar basal position, although not necessarily related to the MC, having archaic ear bones and large and distinct alveoli for functional teeth on the lower and upper jaws.

## Discussion

### Origin and temporal distribution of ziphiids

The oldest putative fossil ziphiid is a fragmentary skull from freshwater deposits of Kenya (Mead, 1975), recently dated to ca 17 Ma (Wichura et al., 2015). Although this specimen was not coded in our phylogenetic analysis due to its incompleteness (the vertex, the most diagnostic part of the skull, is missing), taking into account the advanced mesorostral ossification of the vomer (Mead, 1975) we consider its placement by Wichura et al. (2015) near the hyperodoontines and related taxa as a plausible hypothesis. Used here to constrain the origin of the CZ (Table 4), the geological age of this specimen (17 Ma) is considerably younger than the mean divergence date for the CZ node as estimated by McGowen, Spaulding & Gatesy (2009: 21.98 Ma), but it is close to the CZ divergence date estimated by Hassanin et al. (2012: 16.6 Ma). Within the CZ, the origin of the Berardiinae can be constrained at 15–13.2 Ma, considering the stratigraphic range for the deposits from where *Archaeoziphius microglenoideus* Lambert & Louwye, 2006 originates (Lambert & Louwye, 2006). Concerning other taxa of the CZ, first tentatively assigned to the middle Miocene (14–12 Ma) (Lambert, Bianucci & Post, 2009) *Nazcacetus*, is now considered significantly younger, its age having been reassessed to 7.55–7.3 Ma (Fig. 1) based on the new stratigraphic setting of Cerro Los Quesos (Bianucci et al., in press; Di Celma et al., in press). All the other fossil taxa of the CZ are based on fossil skulls from deep sea phosphorite deposits off South Africa (Bianucci, Lambert & Post, 2007; Bianucci, Post & Lambert, 2008); unfortunately their precise stratigraphic origin is unknown (Bianucci, Lambert & Post, 2007; Bianucci, Post & Lambert, 2008). The phosphatization phase(s) related to the deposition and fossilization of these specimens probably occurred between the middle Miocene and the Pliocene (Siesser, 1978). This broad interval embraces the stratigraphic distribution of most fossil ziphiids, and remains thus poorly informative. Among the modern CZ genera, *Mesoplodon* has a slightly better documented fossil record, including *M. posti* Lambert & Louwye, 2016, from the early Pliocene of Belgium (4.86–3.9 Ma); the latter provides an early Pliocene upper calibration point for the origin of the most species-rich extant ziphiid genus (Lambert & Louwye, 2016).

**Table 4:** Ages used to calibrate the divergence dates of the main ziphiid nodes.

Node	Ma	Determination	State	Source
Ziphiidae	17.5–11.9	Ziphiidae indet.	Ecuador	Bianucci et al. (2005)
*Messapicetus* clade	ca 17.5–13.8	Ziphiidae indet.	Maryland (U.S.A.)	Lambert, Godfrey & Fuller (2010)
Crown Ziphiidae	ca 17	Ziphiidae indet.	Kenya	Wichura et al. (2015)
Berardiinae	15–13.2	*Archaeoziphius microglenoideus*	Belgium	Lambert & Louwye (2006)
*Mesoplodon*	4.86–3.9	*Mesoplodon posti*	Belgium	Lambert & Louwye (2016)

Despite the earlier divergence of their lineages, all the stem ziphiids considered in the phylogenetic analysis are apparently younger than the oldest fossil taxa referred to the CZ. In particular the earliest diverging ziphiid lineages are represented by *Chavinziphius*, here dated to 6.93–6.71 Ma, and *Ninoziphius*, dated to 5.9 or 3.9 Ma (Lambert, de Muizon & Bianucci, 2013). As for the members of MC collected in inland localities of marine sediments, five (*Chimuziphius*, *Choneziphius, Messapicetus*, *Notoziphius*, and *Ziphirostrum*) have a stratigraphic distribution between 10.5 and 7.5 Ma (Tortonian), two (*Aporotus* and *Beneziphius*) have an uncertain to unknown stratigraphic origin, and only one (the holotype of *Tusciziphius crispus* Bianucci, 1997) is dated from the early Pliocene (Bianucci et al., 2001; Bianucci et al., 2016a; Lambert, 2005; Buono & Cozzuol, 2013) (Table 5). The age of all the MC ziphiids from deep sea phosphorite deposits of the North Atlantic seafloor off the Iberian Peninsula has previously been considered highly uncertain (Bianucci et al., 2013), but as a result of a recent micropaleontological study of sediment associated to ziphiid remains (*Globicetus hiberus* Bianucci et al., 2013), at least the fossil assemblage collected off the Portugal coast is now tentatively assigned to the latest Miocene-early Pliocene (Antunes, Legoinha & Balbino, 2015). This assemblage includes cranial remains referred to *Choneziphius*, *Globicetus*, *Imucetus*, and *Tusciziphius*. Although all the above presented data suggest a late Miocene origin and diversification of the MC, a fragmentarily known, unnamed species from the late early-middle Miocene Calvert Formation (Maryland, USA) exhibits thick premaxillae dorsally closing the mesorostral groove (Lambert, Bianucci & Post, 2010); this record may indicate an older origin for the MC. Consequently, we used the upper limit of the stratigraphic range of the Calvert Formation specimen (13.8 Ma) to constrain the age of the MC.

**Table 5:** Geographical origin and age of the fossil species of ziphiids considered for the phylogeny. These data are used to calibrate stratigraphically the tree in Fig. 13 and to elaborate the paleogeographical distribution in Fig. 16.

Species	Region	Age	Source
*Africanacetus ceratopsis*	South Africa	Probably middle Miocene-Pliocene	Bianucci, Lambert & Post (2007)
*Africanacetus* sp.	sub−Antarctic Indian Ocean	Unknown	Gol’din & Vishnyakova (2013)
*Aporotus dicyrtus*	Belgium	Neogene	Lambert (2005)
*Aporotus recurvirostris*	Belgium	Neogene	Lambert (2005)
*Archaeoziphius microglenoideus*	Belgium	Middle Miocene (15–13.2 Ma)	Lambert & Louwye (2006)
*Beneziphius brevirostris*	Belgium	Neogene	Lambert (2005)
*Chavinziphius maxillocristatus*	Peru	Messinian (6.93–6.71 Ma)	this study
*Chimuziphius coloradensis*	Peru	Tortonian (8.9–8.5 Ma)	this study
*Choneziphius leidyi*	Portugal, Spain	Probably Messinian-Zanclean (6.1–4.4 Ma)	Bianucci et al. (2013), Antunes, Legoinha & Balbino (2015)
*Choneziphius planirostris*	Belgium	Tortonian (9.5–7.5 Ma)	Lambert (2005)
*Globicetus hiberus*	Portugal, Spain	Probably Messinian-Zanclean (6.1–4.4 Ma)	Bianucci et al. (2013), Antunes, Legoinha & Balbino (2015)
*Ihlengesi saldanhae*	South Africa	Probably middle Miocene-Pliocene	Bianucci, Lambert & Post (2007)
*Imuoetus piscatus*	Portugal	Probably Messinian-Zanclean (6.1–4.4 Ma)	Bianucci et al. (2013), Antunes, Legoinha & Balbino (2015)
*Izikoziphius angustus*	South Africa	Probably middle Miocene-Pliocene	Bianucci, Lambert & Post (2007)
*Izikoziphius rossi*	South Africa	Probably middle Miocene-Pliocene	Bianucci, Lambert & Post (2007)
*Khoikhoicetus agulhasis*	South Africa	Probably middle Miocene-Pliocene	Bianucci, Lambert & Post (2007)
*Mesoplodon posti*	Belgium	Zanclean (4.86–3.9 Ma)	Lambert & Louwye (2016)
*Mesoplodon slangkopi*	South Africa	Probably middle Miocene-Pliocene	Bianucci, Lambert & Post (2007)
*Messapicetus gregarius*	Peru	Tortonian (9.1–8.5 Ma)	Bianucci, Lambert & Post (2010)
*Messapicetus longirostris*	Italy	Tortonian (10.5–8.14 Ma)	Bianucci et al. (2016a)
cf. *Messapicetus* sp.	Maryland (USA)	Tortonian (10–9 Ma)	Fuller & Godfrey (2007)
*Microberardius africanus*	South Africa	Probably middle Miocene-Pliocene	Bianucci, Lambert & Post (2007)
*Nazcaceus urbinai*	Peru	Late Tortonian (7.55–7.3 Ma)	Lambert, Bianucci & Post (2009)
*Nenga meganasalis*	South Africa	Probably middle Miocene-Pliocene	Bianucci, Lambert & Post (2007)
*Ninoziphius platyrostris*	Peru	Messinian (5.93 Ma) or Zanclean (3.9 Ma)	Lambert, de Muizon & Bianucci (2013)
*Notoziphius bruneti*	Argentina	Tortonian (10 Ma)	Buono & Cozzuol (2013)
*Pterocetus benguelae*	South Africa	Probably middle Miocene-Pliocene	Bianucci, Lambert & Post (2007)
*Tusciziphius atlanticus*	Portugal, South Carolina (USA), Spain	Probably Messinian-Zanclean (6.1–4.4 Ma)	Post, Lambert & Bianucci (2008), Bianucci et al. (2013), Antunes, Legoinha & Balbino (2015)
*Tusciziphius crispus*	Italy	Zanclean (4.12–3.84)	Bianucci (1997), Bianucci et al. (2001)
*Xhosacetus hendeysi*	South Africa	Probably middle Miocene-Pliocene	Bianucci, Lambert & Post (2007)
*Ziphirostrum marginatum*	Belgium	Tortonian (9.5–7.5 Ma)	Lambert (2005)

The new ziphiids described here further support the high past diversity of this family, as already pointed out in previous papers (Bianucci, Lambert & Post, 2007; Bianucci, Post & Lambert, 2008; Buono & Cozzuol, 2013). In fact, excluding taxa based on fragmentary material, the fossil record of ziphiids consists now of 24 genera (of which 23 are extinct) and 32 species, representing the cetacean family with the greatest past diversity (Fig. 13). Although the above mentioned Kenya specimen documents a first appearance of ziphiids during the early Miocene, the stratigraphic distribution of fossil genera based on significant material suggests that these odontocetes became diverse only during the late Miocene.

### Convergent evolution in stem and crown ziphiids

**Figure 14:**
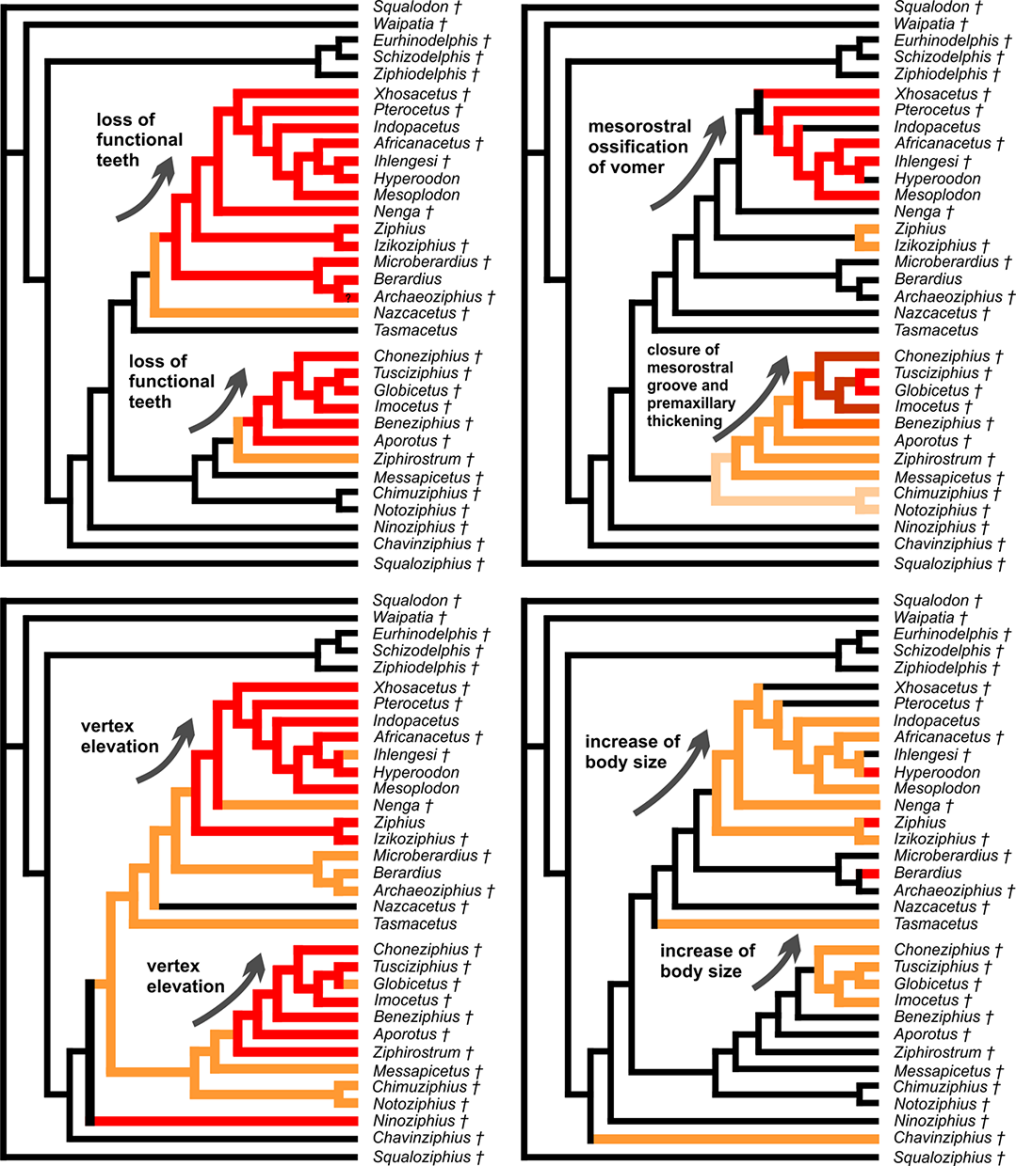
Convergent evolutionary patterns within the Ziphiidae. Morphological changes on the skull, as illustrated by characters taken from the phylogeny, and changes in the body size. The tree is the single most parsimonious as presented in Fig. 13 with the exception of *Aporotus* (here reported as a genus, since the two species form a monophyletic clade). The red color of the bars indicates the most derived status of the character, whereas the white color is for the absence of the character (plesiomorphic condition). † indicates strictly fossil taxa. The trees evidence a similar evolution within the *Messapicetus* clade and the crown Ziphiidae, tentatively correlated to a convergent, progressive adaptation to suction feeding and deep diving.

**Figure 15:**
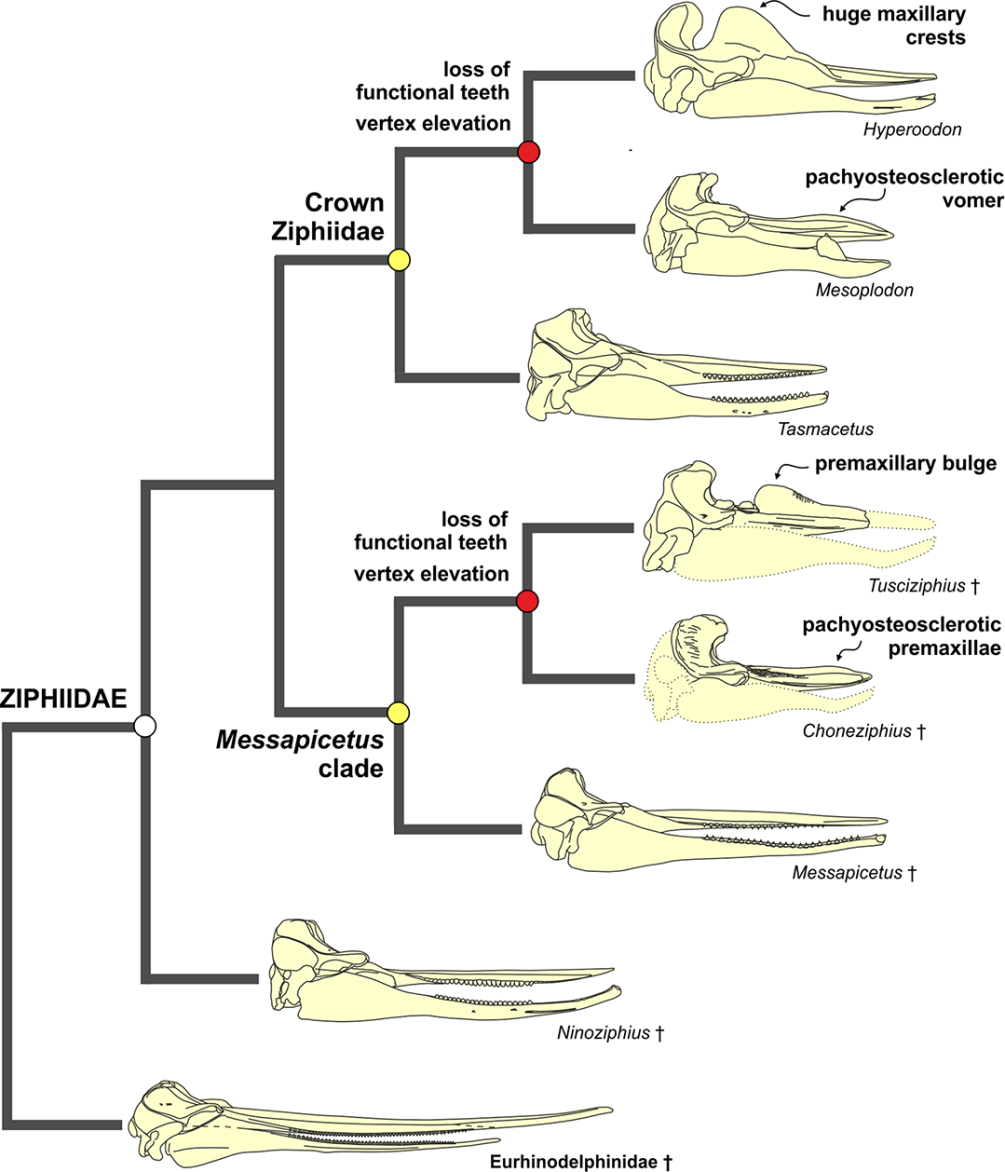
Convergent changes for the skull and teeth within the Ziphiidae. Simplified ziphiid phylogeny showing the main skull and teeth features convergently changing within the *Messapicetus* clade and the crown Ziphiidae. Note that the loss of functional teeth (char. 27) and the vertex elevation (char. 9) are both homologies and homoplasies, whereas the pachyosteosclerotic vomer (char. 2) and pachyosteosclerotic premaxillae (char. 30) are analogies. The skull of the outgroup eurhinodelphinid belongs to *Xiphiacetus*. † indicates strictly fossil taxa.

The new phylogenetic tree proposed here evidences two large ziphiid clades (MC and CZ). In the next paragraphs we discuss several evolutionary trends that are proposed to occur convergently within the two clades and we tentatively correlate these similar trends to (1) a convergent, progressive adaptation to suction feeding and deep diving (Heyning & Mead, 1996; Hooker & Baird, 1999; Johnson et al., 2004), and (2) sexual selection (Dalebout, Steel & Baker, 2008; Gol’din, 2014). Besides the geographical distribution examined below, the following morphological evidence supports this hypothesis (Figs. 14 and 15):

#### Extreme reduction of dentition

Strictly related to suction feeding (Heyning & Mead, 1996; Werth, 2006; Johnston & Berta, 2011), the loss of functional teeth is observed in all extant ziphiids except *Tasmacetus shepherdi* Oliver, 1937, the only species having teeth other than the tusks rooted in the maxilla and in the mandible. In the cladogram proposed here, *Tasmacetus* is the earliest crown ziphiid lineage to branch, supporting the hypothesis that the complete dentition of the latter is a plesiomorphic character. Being intermediate between *Tasmacetus* and all other crown ziphiids, *Nazcacetus* has teeth similar in size and shape to the small erupted teeth of the extant *Mesoplodon grayi* Von Haast, 1876b; interpreted as functional (Von Haast, 1876a; Von Haast, 1876b; Boschma, 1950; Boschma, 1951), the teeth of the latter are not rooted in the maxilla, but instead in the gum (Mead, 1989). By analogy, we suspect that *Nazcacetus* may have used its teeth for grasping its prey. Non-functional, rudimentary, and unerupted smaller teeth have been found embedded in the gum for several other extant ziphiid species (Boschma, 1950; Boschma, 1951; Loch & van Vuuren, 2016): e.g., *Hyperoodon ampullatus* (Forster, 1770), with a diameter of the teeth between 1 and 2 mm, *Mesoplodon bidens*, and *Ziphius cavirostris* Cuvier, 1823.

A progressive reduction of the dentition is similarly observed in the MC. Large maxillary alveoli are observed in *Notoziphius* (Buono & Cozzuol, 2013), the basalmost genus of this clade, together with its sister taxon *Chimuziphius*. Unfortunately, due to the incompleteness of the only specimen preserved, the status of this character is unknown for the latter. The next branching taxon *Messapicetus* similarly bears a complete dentition, with large functional teeth in both the lower and upper jaws (Bianucci, Lambert & Post, 2010; Bianucci et al., 2016a; Lambert, Bianucci & Post, 2010). As a result of the discovery of an exceptional fossil assemblage made of a partial skeleton of *Messapicetus* associated to numerous clupeiform fish remains, it has been proposed that this beaked whale fed on epipelagic fish (Lambert et al., 2015). Morphologically close to *Messapicetus*, but making the next branch in the cladogram, *Ziphirostrum* exhibits a significant reduction of the dentition: its teeth are smaller than in *Messapicetus* and at least the upper teeth are not hold in distinct alveoli (Lambert, 2005). A vestigial alveolar groove with indistinct alveoli is observed in all other more derived ziphiids of the MC, evidencing a trend toward the loss of teeth, as proposed for CZ.

Back to the earliest diverging ziphiids, a functional dentition is observed in *Ninoziphius* (Lambert, de Muizon & Bianucci, 2013) and, judging by the distinct alveoli in the mandible, also in *Chavinziphius*.

#### Pachyostosis and osteosclerosis of the rostral bones

In adult males of several species of extant ziphiids (particularly in most species of *Mesoplodon* and, to a lesser degree, in *Ziphius cavirostris*), a strong ossification of the rostrum is due to the filling of the mesorostral canal by the pachyosteoscleorotic (greatly thickened and compact) vomer (Fraser, 1942; Heyning, 1989; Lambert, de Buffrénil & de Muizon, 2011). The mesorostral ossification of the vomer is absent in stem ziphiids and, among the CZ, in *Berardius*, *Hyperoodon*, *Indopacetus, Microberardius*, *Nazcacetus*, and *Nenga*. However, the absence of ossification may be at least partly due to the small size of the sample, particularly in *Indopacetus* and *Nazcacetus*. Interestingly, in *Berardius*, *Microberardius*, and *Nenga* the mesorostral groove is partly filled by the ossified mesethmoid (Bianucci, Lambert & Post, 2007; Lambert, de Buffrénil & de Muizon, 2011). The mesorostral ossification of the vomer is instead well developed in most fossil ziphiids from the seafloor phosphorite deposits off South Africa, either belonging to the Hyperoodontinae or closely related (Bianucci, Lambert & Post, 2007).

Although the vomer does not fill the mesorostral groove in all stem ziphiids of the MC (with the exception of the aberrant fragmentary rostrum of the holotype of *Ziphirostrum recurvus* (du Bus, 1868), (Lambert, 2005; Lambert, de Buffrénil & de Muizon, 2011)), a trend towards an increased volume of the compact rostrum bones is observed, due to the combination of two characters already pointed out in the phylogeny discussion: I) the closure of the mesorostral groove, first with a dorsomedial contact, then with dorsomedial fusion of the premaxillae (char. 3); and II) the progressive thickening of the compact premaxillae culminating with the voluminous spherical prominence of *Globicetus* and the high premaxillary bulge of *Tusciziphius* (char. 30). Observed to evolve independently in the CZ and the MC, ziphiid pachyosteosclerosis is a clear case of convergent evolution, involving different bones (vomer vs. premaxillae). Interestingly, histological studies revealed strikingly different degrees of remodeling in the different bones of different taxa (de Buffrénil & Lambert, 2011; Lambert, de Buffrénil & de Muizon, 2011; Dumont et al., 2016); for example the inner organization of the pachyosteosclerotic premaxillae of *Aporotus recurvirostris* (a species of the MC) is entirely different from all the rostral bones of *Mesoplodon densirostris* (Blainville, 1817) (a species of the CZ), suggesting that a roughly similar process may have evolved independently in several lineages in response to common selective pressures, possibly linked to the shift to deep waters. Several functional explanations have been provided for the thick and dense rostral bones of ziphiids: as an help for deep diving (ballast) (de Buffrénil et al., 2000), as a structure facilitating sound transmission (Zioupos et al., 1997; Cranford, Krysl & Hildebrand, 2008), or as a structure strengthening the rostrum during intraspecific fights between adult males (Heyning, 1984; McLeod, 2002; Lambert, Bianucci & Post, 2010, Lambert, de Buffrénil & de Muizon, 2011; de Buffrénil & Lambert, 2011). Finally, Gol’din (2014) proposed that ultradense and voluminous rostral structures in extant and fossil ziphiids—not only including the vomer or the premaxillae but also the large maxillary crests—could be used for intraspecific sexual display, being detectable by congeners under the surrounding soft tissues via echolocation. Interestingly, according to our phylogenetic hypothesis the bizarre structures named “antlers inside” by Gol’din (2014) evolved independently in CZ and MC. In particular, within the CZ the enormous (although much more spongy, and possibly related to a head-butting behavior) rostral maxillary crests of the male *Hyperoodon ampullatus*, the protuberant maxillary crests of *Africanacetus*, and the dorsoventrally high, compact rostrum of adult males of several hyperoodontine species are particularly conspicuous. Instead, within the MC we observed voluminous premaxillary prominences and bulges in *Globicetus* and *Tusciziphius*, and spur-like rostral maxillary crests in *Imocetus*. Surprisingly, a medial bulge somewhat similar in outline to the one of *Tusciziphius* is present in isolated fossil rostra of “*Mesoplodon” tumidirostris* Miyazaki & Hasegawa, 1992 collected on the North Pacific seafloor (Miyazaki & Hasegawa, 1992; Kohno, 2002). Although the generic attribution of this species is questionable due to the incompleteness of the holotype and referred specimens, the fact that the high bulge is made of the vomer completely filling the mesorostral groove suggests a close relationship with hyperoodontines. Pending the discovery of more complete skulls of “*M.” tumidirostris*, we propose that a peculiar bulge on the rostrum evolved independently in the CZ and in the MC, but involving different bones: the vomer in “*M.” tumidirostris* and the premaxillae in *Tusciziphius*.

Like *Ninoziphius*, and the other basalmost beaked whales, *Chavinziphius* lacks any thickening of the premaxillary and vomer. However, *Chavinziphius* exhibits unusually robust and elevated rostral maxillary crests that are reminiscent of the rostral maxillary crests of *Berardius*. These crests could represent insertion areas for strong facial muscles, as observed in extant ziphiids (Heyning, 1989) and as already proposed for several fossil taxa (Bianucci et al., 2013). Alternatively, according to the “antlers inside” hypothesis (Gol’din, 2014) *Chavinziphius* could also have independently evolved internal structures for sexual display.

#### Changes in the morphology of the facial area of the skull

All ziphiids are characterized by an elevated vertex and transverse premaxillary crests, two bony features linked to the forehead soft anatomy and particularly to the production of echolocation sounds (Moore, 1968; Heyning, 1989; Cranford, Krysl & Hildebrand, 2008). Both in the CZ and in the MC, a general trend towards further elevation of the vertex is observed (char. 9, state 2). Similarly, in both clades we observe a trend towards the widening of the premaxillary crests and their anterior projection, leading to some degree of overhanging above the bony nares especially conspicuous in e.g., *Imocetus*, *Globicetus*, *Hyperoodon*, and *Ziphius* (char. 7 state 3). Moreover, also considered as related to the echolocation system (Heyning, 1989; Cranford, Amundin & Norris, 1996), the asymmetry of the premaxillary sac fossae (char. 5) is the greatest in *Hyperoodon* and *Ziphius* within the CZ and in *Globicetus*, *Tusciziphius*, and *Choneziphius* within the MC.

#### Increase of body size

Using estimates of body length calculated based on the postorbital width of the skull according to Bianucci, Post & Lambert (2008) (see also Pyenson & Sponberg, 2011), changes in the body size of ziphiids have been investigated in Mesquite 2.74 (Maddison & Maddison, 2010) with the phylogenetic tree obtained in the cladistic analysis as a backbone. The results obtained here are partly similar to the ones already discussed in Lambert, de Muizon & Bianucci (2013). In particular, most species of the fossil genera have a smaller size than species of the extant genera, and the largest species of the sample are in the extant genera *Berardius*, *Hyperoodon*, and *Ziphius*. However, differing from the analysis by Lambert, de Muizon & Bianucci (2013), an increase in body size is observed in several stem ziphiids: in *Choneziphius*, *Globicetus*, *Imocetus*, and *Tusciziphius*, here considered as the most derived genera of the MC for several morphological characters discussed above, and, surprisingly, in *Chavinziphius*, the earliest diverging beaked whale. In summary, this new analysis further supports the hypothesis that an increase in body size occurred independently in several ziphiid lineages. Possible functional explanations for this trend have been discussed elsewhere (see Lambert, de Muizon & Bianucci, 2013). We still wish to highlight the hypotheses that a larger body size may represent a way (1) to metabolically improve the diving capacity (Schreer & Kovacs, 1997; Noren & Williams, 2000), and/or (2) to minimize the drag during diving (Watanabe et al., 2011); these two hypotheses are in agreement with the deep diving behavior observed in all extant CZ and proposed for the most derived genera of the MC.

### Paleobiogeography of ziphiids

**Figure 16:**
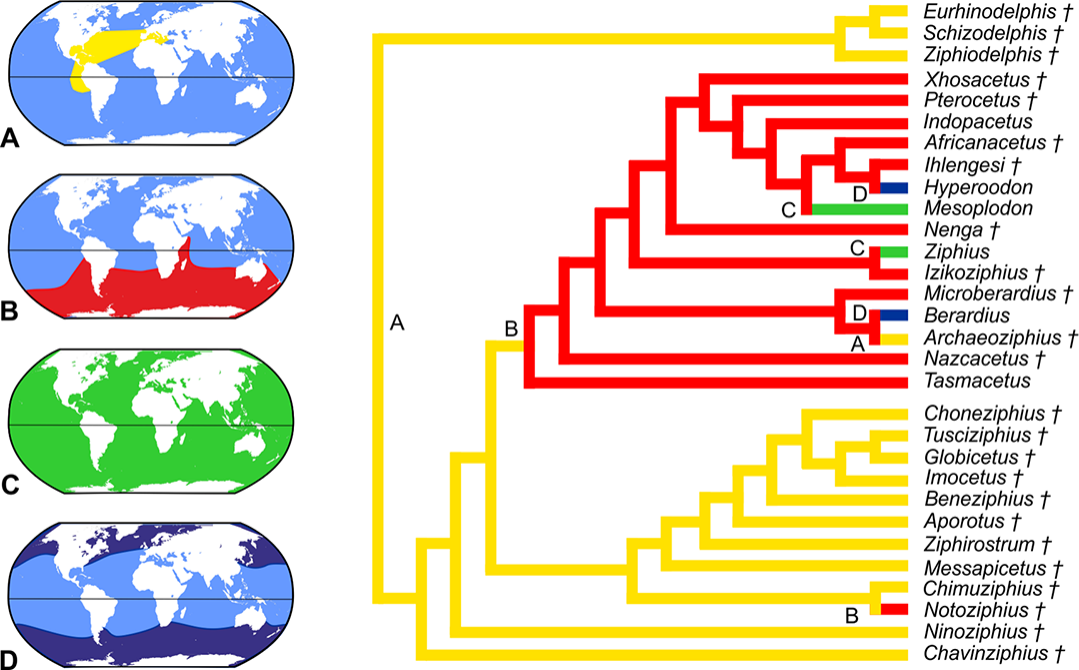
Paleobiogeography of Ziphiidae. Changes in the geographical distribution of the Ziphiidae based on the morphological cladistic analysis (see Table 5). Four main distributional patterns are recognized: (A, yellow) southeastern Pacific and North Atlantic oceans and the Mediterranean Sea; (B, red) southern oceans; (C, green) cosmopolitan; (D, white) antitropical distribution in cold to temperate waters. † indicates strictly fossil taxa.

Starting from data about known localities of extinct ziphiids and the distribution of extant genera plotted in the phylogenetic tree using Mesquite 2.74, past major changes in the geographical distribution of ziphiids were investigated. Four large distribution areas for fossil and extant ziphiids are defined, allowing the preliminary discussion of the following hypothetical dispersal events (Fig. 16):

1. With the exception of *Notoziphius*, all stem ziphiids originate from an area including the southeastern Pacific and North Atlantic oceans and the Mediterranean Sea. Direct biotic interchanges between the Pacific and Atlantic oceans were possible during the Miocene via the Central America Seaway (Jacobs, Haney & Louie, 2004). In this context, the Peruvian *Messapicetus gregarius* and the Italian *M. longirostris* are considered as sister species with an antitropical distribution (Bianucci et al., 2016a). Considering that the earliest diverging stem ziphiids (*Chavinziphius* and *Ninoziphius*) and two early branches of the MC (*Chimuziphius* and *Messapicetus*) are recorded from Peru and that, at least for *Messapicetus*, relatively robust evidence indicates a coastal habitat (Lambert et al., 2015), we hypothesize that the nutrient-rich coastal waters of the southeastern Pacific represented an important area of radiation for stem ziphiids.
  The most derived ziphiids of the MC are all from the North Atlantic, suggesting a possible diversification of this presumably deep diving lineage in this more restricted area. Such a North Atlantic concentration of derived members of the MC supports the hypothesis that these ziphiids evolved separately (geographical segregation) from the early members of the CZ.
2. Most crown ziphiids, both fossil and extant, are from southern oceans, particularly from temperate and cold waters, indicating that these highly productive waters characterized nowadays by an elevated marine mammal species richness (Kaschner et al., 2011) may have played an important role in the diversification of modern, deep diving ziphiids. Several extant species of crown ziphiids have a circum Antarctic distribution and a similar distribution is proposed for the extinct *Africanacetus*, recorded from both the seafloor off South Africa and the sub-Antarctic Indian Ocean (Gol’din & Vishnyakova, 2013).
3. Although *Mesoplodon* and *Ziphius* are cosmopolitan genera, their possible origin and/or first diversification in southern oceans is supported by fossil records from the South African seafloor (*Mesoplodon slangkopi* Bianucci, Lambert & Post, 2007 and *Ziphius* sp.). Moreover, many of the extant *Mesoplodon* species have a circum Antarctic distribution (Mead, 1989).
4. Prevalently occupying cold to temperate waters of northern and southern oceans, sister species of the extant genera *Berardius* and *Hyperoodon* display an antitropical distribution. Their modern distribution and diversity could result from a large-scale dispersal event (possibly from the southern hemisphere) during a temporarily cooler Pleistocene phase, followed by vicariant speciation when global warmer conditions and warm water equatorial barriers reestablished (Davies, 1963; Hare, Cipriano & Palumbi, 2002). The large body size of *Berardius* and *Hyperoodon* (Fig. 14) may be interpreted at least in part as an adaptation to the cold, high latitude waters (Lambert, de Muizon & Bianucci, 2013).

## Conclusions

*Chavinziphius maxillocristatus* and *Chimuziphius coloradensis* are two new late Miocene stem ziphiid species based on skull remains respectively from the Messinian of Cerro Los Quesos and from the Tortonian of Cerro Colorado, two marine vertebrate-rich localities of southern Peru.

The new phylogenetic analysis here proposed identifies *Chavinziphius* as the earliest diverging ziphiid, and places *Chimuziphius* as sister taxon of the Patagonian *Notoziphius*, in a basal position within the MC.

The fossils here described further support the high past diversity of Ziphiidae, the richest among cetaceans for the number of genera found in the fossil record (24, two extant and 22 extinct) and species (32, all extinct) based on significant fossil material. Appearing in the fossil record during the early Miocene, ziphiids only became diverse and well represented during the late Miocene.

Our phylogenetic analysis evidences two main clades within the beaked whales: the CZ and the MC. Both lineages are proposed to follow similar evolutionary trends, and this convergent evolution is hypothesized to have occurred in response to common selective pressures, possibly linked to the ecological shift to deep diving and suction feeding (see Lindberg & Pyenson (2007) for an elaborated scenario relating echolocation abilities and progressive migration to deeper feeding areas for cephalopod-feeding odontocetes). Our hypothesis is supported by the following morphological evidence:

– In both the MC and the CZ, a trend towards the progressive reduction of the functional dentition is observed, and most likely correlated to an adaptation to suction feeding.
– Progressive increase of compactness and thickening (pachyosteosclerosis) of the rostrum bones occurs during the evolution of the MC, through dorsal closure of the mesorostral groove by the premaxillae and thickening of the latter, and of the CZ, through the filling of the mesorostral groove with the pachyosteosclerotic vomer and increased compactness of surrounding bones.
– Strictly linked to the production and transmission of high-frequency, echolocation sounds in extant odontocetes, the morphology of the facial area of the cranium follows similar evolutionary trends in the MC and the CZ, with a particular emphasis on the elevation of the vertex, the widening of the transverse premaxillary crests partly overhanging of the bony nares, and the increased asymmetry of the premaxillary sac fossae.
– Our dataset indicates not only a general trend towards larger body size both in the MC and in the CZ, but also an independent increase of body size in several more exclusive ziphiid clades.

The paleobiogeographical analysis nicely matches the phylogenetic relationships: all the stem ziphiids, including the MC, are evidenced to have first radiated in a large area including the southeastern Pacific and the North Atlantic oceans; the earliest diverging stem ziphiids probably lived in shallow waters, like the nutrient-rich coastal waters of the Peruvian coast, whereas more derived members of the MC, displaying morphological clues for deep diving and suction feeding adaptations, all originate from the North Atlantic Ocean and the Mediterranean Sea; finally, a majority of the CZ are instead found in deep water regions of the southern oceans, with a possible subsequent dispersal to all other oceans for *Mesoplodon* and *Ziphius* and to the cooler waters of the northern oceans for *Berardius* and *Hyperoodon*.

## Supplemental Information

10.7717/peerj.2479/supp-1 Supplemental Information 1 Supplemental File S1. Characters and matrix used for the phylogenetic analysis.

10.7717/peerj.2479/supp-2 Supplemental Information 2 Supplemental File S2. Ontogenetic change of size and number of dorsal infarorbital foramina on the maxilla near the base of the rostrum in *Berardius* spp.

## Institutional abbreviations

MNHN: Muséum National d’Histoire Naturelle, Paris, France
MUSM: Museo de Historia Natural, Universidad Nacional Mayor de San Marcos, Lima, Peru

## Additional Information and Declarations

### Field Study Permissions

The following information was supplied relating to field study approvals (i.e., approving body and any reference numbers): The fieldwork activity and the fossil collection and study was regulated by the conditions reported in the following agreement: “Convenio de Cooperación Científica entre el Museo de Historia Natural de la Universidad Nacional Mayor de San Marcos y el Departamento de Ciencias de la Tierra de la Universidad de Pisa.” All fossils examined in this study are curated in the Museo de Historia Natural de la Universidad Nacional Mayor de San Marcos, a Peruvian public institution.

### Data Deposition

The following information was supplied regarding data availability: The raw data has been supplied as Supplemental Dataset Files.

### New Species Registration

The following information was supplied regarding the registration of a newly described species: Publication LSID: urn:lsid:zoobank.org:pub:4CB30374-BA31-41CB-A2AB-D61BB5F084FB; *Chavinziphius* LSID: urn:lsid:zoobank.org:act:C861C203-41A5-41DB-80CF-7A57AE8EF9A6; *Chavinziphius maxillocristatus* n. gen. n. sp. LSID: urn:lsid:zoobank.org:act:1DDA1BB3-6665-45E8-9BCB-15DC48665E71; *Chimuziphius* LSID: urn:lsid:zoobank.org:act:3F4BFE17-0023-434F-9969-AD8C77DBC149; *Chimuziphius coloradensis* n. gen. n. sp. LSID: urn:lsid:zoobank.org:act:0AA663DE-3AEC-4849-B8C0-A1FB9D678E35.
