# Supplementary material for: New beaked whales from the late Miocene of Peru and evidence for convergent evolution in stem and crown Ziphiidae (Cetacea, Odontoceti)
Source: PeerJ. 2016 Sep 20;4:e2479. doi: 10.7717/peerj.2479 (PMC5036081; doi:10.7717/peerj.2479)
Supplement: Supplemental Information 1 — Characters and matrix used for the phylogenetic analysis. [file peerj-04-2479-s001.pdf]

Supplemental File S1

to accompany the article:

New beaked whales from the late Miocene of Peru and evidence for convergent evolution in stem and crown Ziphiidae (Cetacea, Odontoceti)

Giovanni Bianucci [1], Claudio Di Celma [2], Mario Urbina[3], Olivier Lambert[4]

[1] Dipartimento di Scienze della Terra, Università di Pisa, Via Santa Maria, 53, Pisa 56126, Italy
[2] Scuola di Scienze e Tecnologie, Università di Camerino, Camerino 62032, Italy
[3]Departamento de Paleontologia de Vertebrados, Museo de Historia Natural-UNMSM, 1256, Avenida Arenales, Lima 14, Peru
[4]Institut royal des sciences naturelles de Belgique, D.O. Terre et Histoire de la Vie, 29, rue Vautier, Brussels 1000, Belgium

published in PeerJ

Characters and matrix used for the phylogenetic analyses and maximum parsimonious cladograms obtained with equally weighted and implied weighted analyses

Tables of content:

1. List of characters used in the cladistic analysis p. S2

2. Matrix p. S7

3. Maximum parsimonious cladograms pp. S9-S13

4. References p. S14

List of characters used in the cladistic analysis

1. *Length of the rostrum* (Bianucci *et al.*, 2010) (ordered): ratio between rostrum length and condylobasal length > 0.70 (0); ratio between 0.63 and 0.70 (1); ratio < 0.63 (2).
2. *Mesorostral groove* (Bianucci *et al.*, 2007, modified in Bianucci *et al.*, 2010): empty (0); filled by the mesorostral ossification of the vomer, without median suture between the lateral walls of the vomer in the rostrum base area (1); filled by the mesorostral ossification of the vomer, with median suture between the lateral walls of the vomer in the rostrum base area (2).
3. *Mesorostral groove* (Bianucci *et al.*, 2007, modified) (ordered): open for the proximal third of the rostrum (0);anteroposteriorly elongated contact between unfused premaxillae (1);medial fusion or contact of the premaxillaeextending posteriorly in front of the premaxillary foramina (2); medial fusion extending posteriorly behind and near the premaxillary foramina (3); medial fusion extending posteriorly until the bony nares (4).
4. *Prenarial basin* (Lambert, 2005c): absent (0); laterally margined by the premaxilla (1); laterally margined by a thick strip of maxilla (2).
5. *Asymmetry of the premaxillary sac fossae* (Lambert 2005c, modified in Bianucci *et al.*, 2010): absent or weak, ratio between the widths of the left and right premaxillary fossae > 0.65 (0); moderate to high, ratio ≤ 0.65 (1).
6. *Premaxillary sac fossa laterally overhanging the maxilla* (Lambert 2005c): no (0); yes (1).
7. *Ascending process of the premaxilla in lateral view* (Bianucci *et al.*, 2007) (ordered): rectilinear (0); slightly concave (1); concave with posterodorsal portion vertical (2); concave with posterodorsal portion partly overhanging the bony nares (3).
8. *Constriction on the ascending process of the right premaxilla (between premaxillary sac fossa and premaxillary crest)* (Bianucci *et al.*, 2007) (ordered): roughly absent, ratio between the minimal width of ascending process of premaxilla and the width of right premaxillary crest > 0.80 (0); moderate constriction, ratio between 0.80 and 0.61(1); strong constriction, ratio < 0.61 (2).
9. *Vertex elevation* (Bianucci *et al.*, 2007, modified in Lambert et al., 2013) (ordered): absent to weak, ratio between the vertical distance between the dorsal margin of the maxilla at the

rostrum base and the top of the vertex and the width of the premaxillary sac fossae < 0.70 (0); moderate, ratio between 0.70 and 1.0 (1); strong, ratio > 1.0 (2).

10. *Premaxillary crest direction* (taken on the anterior edge in dorsal view) (Bianucci *et al.*, 2007 , modified in Lambert et al., 2013): crest transversely directed (0); crest anterolaterally directed (1); crest posterolaterally directed (2); left crest anterolaterally directed and right crest posterolaterally directed (3). Cannot be scored for taxa lacking the premaxillary crests.

11. *Width of the premaxillary crests* (Bianucci *et al.*, 2010) (ordered): small, ratio between the width of premaxillary crests (from the lateralmost point of the right crest to the lateralmost point of the left crest) and the width of premaxillary sac fossae< 1.0(0); moderate, ratio from 1.0 to 1.25 (1); large, ratio > 1.25 (2). Cannot be scored for taxa lacking the premaxillary crests.

12. *Distance between premaxillary crests* (Bianucci *et al.*, 2007): large, ratio between the minimum distance between the right and left premaxillary crests and the width of the premaxillary sac fossae> 0.25 (0); reduced, ratio ≤ 0.25 (1). Cannot be scored for taxa lacking the premaxillary crests.

13. *Nasal elongation* (Bianucci *et al.*, 2010) (ordered): short nasal, ratio between the length of medial suture of nasals on vertex and the maximum width of nasals< 0.4(0); elongated, ratio between 0.4 and 1.1 (1); very elongated, ratio > 1.1, with the anterior tip of nasal anterior to the premaxillary crest (2).

14. *Anteromedial excavation of the dorsal surface of the nasal* (Bianucci *et al.*, 2007) (ordered): no (0); slight anteromedial concavity (1); well-defined anteromedial depression (2); deep excavation (3).

15. *Inclusion of the nasal in the premaxillary crest* (Bianucci *et al.*, 2007) (ordered): no (0); for a short distance along the posteromedial angle of the premaxillary crest (1); until about halfway along the medial margin of the crest (2); reaching the anteromedial margin of the crest (3). Cannot be scored for taxa lacking the premaxillary crests.

16. *Contact between nasal and premaxillary crest* (Bianucci *et al.*, 2007, modified in Lambert et al., 2013) (ordered): reduced, on the posterior half of the nasal (0); on more than half the length of the nasal but not the whole length (1); along the whole length of the nasal (2). Cannot be scored for taxa lacking the premaxillary crests.

17. *Interparietal or frontals as an isolated rounded protuberance on the posterior part of the vertex* (Bianucci *et al.*, 2007): no (0); yes (1).

18. *Anteromedial margin of the supraoccipital* (Bianucci *et al.*, 2007, modified in Lambert et al., 2013): roughly reaching dorsally the level of the vertex (0); distinctly lower than the dorsal margin of the vertex (1).

19. *Angle formed by the basioccipital crests in ventral view* (Geisler & Sanders, 2003, modified in Bianucci *et al.*, 2010): < 50° (0); ≥ 50° (1).

20. *Fan-shaped posterior bullar facet of the periotic* (Bianucci *et al.*, 2010): no (0); yes (1).

21. *Transverse thickening of the anterior process of the periotic* (Fordyce, 1994, modified in Bianucci *et al.*, 2010): absent or slight thickening (0); marked thickening (1).

22. *Anterior spine of the tympanic* (Lambert, 2005b, modified in Bianucci *et al.*, 2010) (ordered): individualized strong anterior spine (0); anterior margin pointed but without a marked thickening (1); with a more or less rectilinear anterior margin (2).

23. *Sigmoid process of the tympanic in lateral view* (Lambert 2005b, modified in Lambert et al., 2013) (ordered): high, without distinct posteroventral corner (0); posteroventral corner present and posterior margin perpendicular to long axis of the tympanic (1); posteroventral corner posteriorly projected (2).

24. *Dorsal margin of the involucrum of the tympanic cut by an indentation* (Lambert 2005b, modified in Lambert et al., 2013): absent (0); present visible in medial and/or dorsal view (1).

25. *Shape of the facets for the incus on the malleus, in posteromedial view* (Bianucci *et al.*, 2010): elongated, ratio between the main horizontal axis length and the main vertical axis length = ha/va< 1.0 (0); approximately circular, ha/va≥ 1.0 (1).

26. *Tuberculum of the malleus* (Lambert 2005b, modified in Lambert et al., 2013): elongated tuberculum, ratio between the tuberculum and malleus lengths = lt/lm > 0.50 (0); short tuberculum, lt/lm ≤ 0.50 (1).

27. *Functional teeth in well-defined alveoli* (excluding tusks)(Bianucci *et al.*, 2010, modified): yes (0); no (1).

28. *Tusks on the mandibles* (Bianucci *et al.*, 2007, modified in Bianucci *et al.*, 2010): absent (0); two enlarged pairs, apical to sub-apical (1); one enlarged pair, apical (2); one enlarged pair, not apical (3).

29. *Length of the symphyseal portion of the mandibles* (Bianucci *et al.*, 2010, modified in Lambert et al., 2013): elongated, ratio between length of the symphyseal portion and total length: ≥ 0.35 (0); short, ratio < 0.35 (1).

30. *Thickening of the compact premaxillae (pachyosteosclerosis) on the rostrum* (ordered): absent (0); weak thickening (1); marked thickening (2); high and voluminous prominence (3).

31. *Deep excavation of the premaxillary sac fossae* (Lambert et al., 2013): absent (0); present (1).

32. *Premaxillary crest on the vertex* (Muizon, 1991, modified in Lambert et al., 2013): absent (0); present (1).

33. *Extremely ossified trapezoidal vertex* (Lambert et al., 2013): absent (0); present (1).

34. *Level of the premaxillary foramen* (Lambert et al., 2013) (ordered): distinctly anterior to the antorbital notch (0); roughly at the level of the antorbital notch (1); distinctly posterior to the antorbital notch (2).

35. *Hamular fossa of the pterygoid sinus* (Lambert et al., 2013): small, not reaching anteriorly the antorbital notch (0); wide, extending anteriorly on the palatal surface of the rostrum (1).

36. *Apices of the right and left hamular processes of the pterygoids* (Lambert et al., 2013)(ordered): contact medially, forming together a medial point posteriorly directed (0); diverge posterolaterally, forming together a concave V-shaped posterior margin (1); as for the state 1 but with less excavated and U-shaped posterior margin (2).

37. *Excavation of the apex of the hamular process by the fossa for the hamular lobe of the pterygoid sinus* (Lambert et al., 2013): no (0); yes (1).

38. *Anteroposterior shortening of the zygomatic process of the squamosal* (Lambert et al., 2013): absent, elongated zygomatic process, ratio between the height of the process (from the anterior tip of the process to the external auditory meatus) and the length of the process (from the dorsal margin of the zygomatic process to the ventral margin of the postglenoid process) < 1.10 (0); short, ratio ≥ 1.10 (1).

39. 39.*Ventral margin of the postglenoid process of the squamosal in lateral view* (Lambert et al., 2013, modified)(ordered): clearly more ventral than the ventral margin of the paroccipital process of the exoccipital (0); approximately at the same level as the ventral margin of the paroccipital process (1); clearly more dorsal than the ventral margin of the paroccipital process (2).

40. *Extent of the lateral tuberosity of the periotic in ventral view* (Lambert et al., 2013): transversely short (0); laterally elongated (1).

41. *Dorsal keel on the posterior process of the periotic* (Lambert et al., 2013): present on the whole length of the process (0); absent or poorly individualized (1).

42. *Degree of fusion of the symphysis on the mandibles* (Lambert et al., 2013): reduced to absent (0); strong, dentaries nearly completely fused (1).

43. *Shape of the section of the symphyseal portion of the mandibles* (Muizon, 1991, modified in Lambert et al., 2013): triangular, pinched in posteroventral region (0); half-circled (1).

44. *Precoronoid crest* (Fordyce *et al.*, 2002): absent, dorsal margin of the mandible rectilinear or slightly concave from the alveolar groove to the coronoid process (0); present, dorsal margin distinctly convex (1).

45. *Stomach anatomy* (Mead, 2007) (unordered): one main stomach and one pyloric stomach (0); two main stomachs and one pyloric stomach (1); two main stomachs and two pyloric stomachs (2). This character could only be coded for the extant ziphiids.

46. *Pairs of double-headed ribs* (Lambert et al., 2013): 8 or more (0); less than 8 (1).

47. *Anteroposterior length of the temporal fossa* (Lambert et al. 2015, modified) (ordered): ratio between horizontal length of the temporal fossa and the length of the neurocranium> 0.44 (0); ratio from 0.44 to 0.35 (1); ratio < 0.35 (2).

48. *Dorsal infarorbital foramina on the maxilla near the base of the rostrum*: cluster of three or more relatively small foramina (0); one large foramen sometimes associated to other,significantly smaller foramina (1)

49. *Excrescences on the dorsal surface of the maxilla on the posterior half of the rostrum*: no (0); yes (1).

50. *Posterior transverse narrowing of nasals and frontal at the vertex narrower than the nasals*: absent (0); present (1)

51. *Fusion of cervical vertebrae in adult specimen* (ordered): all cervical vertebrae free (0); 2-3 cervical vertebrae fused (1); 4-6 cervical vertebrae fused (2); 7 cervical vertebrae fused (3)

Character/taxon matrix

Data matrix of 51 characters for six outgroups (*Eurhinodelphis*, *Schizodelphis*, *Squalodon*, *Squaloziphius*, *Waipatia*, and *Ziphiodelphis*) and 22 extinct and extant ziphiid taxa. 14 characters with multiple states are treated as ordered (1, 3, 7, 8, 9, 11, 13, 14, 15, 16, 22, 23, 36, 39, 47, 51), and 6 (2, 4, 10, 28, 34, 39, 45) as unordered; 0, primitive state; 1, 2, 3, derived states; a, variable between 0 and 1; b, variable between 1 and 2; c, variable between 2 and 3; d, variable between 1 and 3; n, character inapplicable; ?, missing character.

	1	2	3	4	5	6	7	8	9	10	11	12	13	14	15	16	17	18	19	20	21	22	23	24	25
Squalodon	0	0	0	0	0	0	0	0	0	n	n	n	0	0	n	n	0	0	0	0	0	0	0	0	0
Waipatia	2	0	0	0	0	0	1	0	0	n	n	n	0	0	n	n	0	0	1	0	0	1	0	0	?
Eurhinodelphis	0	0	1	0	0	0	1	0	0	n	n	n	0	0	n	n	0	0	0	0	0	1	?	1	0
Schizodelphis	0	0	1	0	0	0	1	0	0	n	n	n	0	0	n	n	0	0	0	0	0	1	0	1	0
Ziphiodelphis	0	0	1	0	0	0	1	0	0	n	n	n	0	0	n	n	0	0	0	0	0	1	0	1	0
Squaloziphius	?	0	?	0	0	0	0	0	0	0	1	n	1	0	n	n	0	0	1	?	?	?	?	?	?
Xhosacetus	?	2	0	0	0	0	2	1	2	0	1	0	1	0	1	2	0	0	?	?	?	?	?	?	?
Pterocetus	1	2	0	0	0	0	2	2	2	0	1	0	1	1	1	2	0	0	?	?	?	?	?	?	?
Indopacetus	1	?	0	0	0	0	2	2	2	2	2	0	1	2	1	1	0	0	1	1	1	2	?	1	?
Africanacetus	1	2	0	0	0	0	3	2	2	2	1	0	1	3	2	2	0	0	?	?	?	?	?	?	?
Ihlengesi	?	2	0	0	0	0	3	2	1	2	0	0	1	3	2	2	0	?	?	?	?	?	?	?	?
Mesoplodon	1	2	0	0	0	0	3	2	2	2	1	1	1	3	1	2	0	0	1	1	1	2	2	a	1
Hyperoodon	1	2	0	0	1	0	3	2	2	2	2	1	1	3	3	2	0	0	1	1	1	2	2	1	?
Ziphius	2	1	0	1	1	1	3	0	2	1	0	0	2	0	0	a	0	0	1	1	1	2	2	1	1
Izikoziphius	1	1	0	0	0	1	3	0	2	1	0	0	2	0	0	0	0	0	?	?	?	?	?	?	?
Microberardius	?	?	0	0	0	0	1	0	1	0	0	0	1	?	0	1	1	1	?	?	?	?	?	1	?
Berardius	1	0	0	0	0	0	1	0	1	0	1	0	1	0	0	1	1	1	1	1	1	2	2	1	?
Archaeoziphius	?	0	?	0	0	0	1	0	1	0	1	0	1	0	a	1	?	1	1	?	?	?	?	?	?
Nazcacetus	1	0	0	0	0	0	2	1	0	0	0	0	1	1	0	1	0	0	?	1	1	?	?	?	?
Tasmacetus	1	0	0	0	0	0	2	2	1	0	0	0	1	1	0	1	0	0	1	1	1	2	2	0	1
Choneziphius	b	0	4	0	1	1	c	a	2	1	1	0	1	?	0	0	0	0	?	?	?	?	?	?	?
Tusciziphius	1	0	4	0	1	0	3	2	b	1	1	0	2	1	0	2	0	0	1	?	?	?	?	?	?
Globicetus	1	0	4	0	1	0	3	2	1	3	1	1	2	1	0	2	0	0	?	?	?	?	?	?	?
Imocetus	2	0	4	1	0	0	3	2	2	3	1	0	1	1	?	2	0	0	?	?	?	?	?	?	?
Beneziphius	2	0	3	2	0	0	2	1	2	1	1	0	1	1	0	0	0	0	?	?	?	?	?	?	?
Ziphirostrum	1	0	2	2	0	0	2	1	2	1	1	0	1	1	0	0	0	0	?	?	?	?	?	?	?
Aporotusrec	1	0	2	2	?	0	3	?	2	1	?	0	1	0	0	0	?	?	?	?	?	?	?	?	?
Aporotusdicyrt	1	0	2	2	0	0	1	0	2	1	1	0	1	0	0	0	0	0	?	?	?	?	?	?	?
Messapicetus	0	0	2	2	0	0	2	2	1	1	1	0	0	1	0	0	0	0	0	0	0	0	2	a	0
Ninoziphius	0	0	0	0	0	0	1	?	2	1	?	0	1	0	0	0	0	0	a	0	0	2	1	1	?
Chimuziphius	?	0	1	0	0	0	1	0	1	1	1	0	1	1	0	0	0	0	?	?	?	?	?	?	?
Nenga	1	0	0	0	0	0	1	0	1	0	0	0	1	0	1	1	0	0	?	?	?	?	?	?	?
Notoziphius	?	0	?	0	0	0	1	0	1	1	1	0	1	1	0	0	0	0	0	?	?	?	?	?	?
Caesuziphius	a	0	?	0	0	0	1	b	1	0	b	0	1	0	0	1	0	0	1	?	?	?	?	?	?

	26	27	28	29	30	31	32	33	34	35	36	37	38	39	40	41	42	43	44	45	46	47	48	49	50	51
Squalodon	0	0	0	0	0	0	0	0	0	0	0	0	0	0	0	0	0	0	0	?	?	0	0	0	0	0
Waipatia	?	0	0	1	0	0	0	0	0	0	?	?	0	1	0	0	0	0	0	?	?	0	0	0	0	0
Eurhinodelphis	1	0	0	?	0	0	0	0	1	0	?	?	1	1	0	0	?	?	?	?	?	1	0	0	0	0
Schizodelphis	1	0	0	0	0	0	0	0	1	0	0	0	1	1	0	0	0	0	0	?	?	1	0	0	0	0
Ziphiodelphis	1	0	?	0	0	0	0	0	1	0	?	?	1	1	0	0	1	0	0	?	?	0	0	0	0	0
Squaloziphius	?	?	?	?	0	0	1	0	2	0	0	0	0	0	?	?	?	?	?	?	?	0	0	0	0	?
Xhosacetus	?	1	?	?	0	0	1	0	1	1	?	?	?	?	?	?	?	?	?	?	?	?	1	0	0	?
Pterocetus	?	1	?	?	0	0	1	0	0	?	?	?	?	?	?	?	?	?	?	?	?	?	1	0	0	2
Indopacetus	?	1	2	1	0	0	1	0	a	1	2	1	1	2	1	1	0	0	1	?	1	1	1	0	0	?
Africanacetus	?	1	?	?	0	0	1	0	1	1	?	?	?	?	?	?	?	?	?	?	?	?	1	0	0	?
Ihlengesi	?	1	?	?	0	0	1	0	2	?	?	?	?	?	?	?	?	?	?	?	?	?	1	0	0	1
Mesoplodon	1	1	3	1	0	0	1	0	1	1	2	1	1	2	1	1	0	0	1	2	1	2	1	0	0	3
Hyperoodon	?	1	2	1	0	0	1	0	2	1	2	1	1	2	1	1	0	0	1	0	1	2	1	0	0	2
Ziphius	1	1	2	1	0	0	1	0	1	1	2	1	1	2	1	1	0	0	1	0	1	2	1	0	0	?
Izikoziphius	?	1	?	?	0	0	1	0	1	?	?	?	?	?	?	?	?	?	?	?	?	?	1	0	0	?
Microberardius	?	?	?	?	0	0	1	0	1	?	?	?	?	?	?	?	?	?	?	?	?	?	1	0	1	1
Berardius	?	1	1	1	0	0	1	0	1	1	2	1	1	2	1	1	0	1	1	1	0	2	1	0	1	0
Archaeoziphius	?	?	?	?	0	0	1	0	?	1	?	1	1	2	?	?	?	?	?	?	?	?	?	?	1	1
Nazcacetus	?	1	2	1	0	0	1	0	1	1	?	?	1	2	1	1	0	0	1	?	?	1	0	0	1	2
Tasmacetus	1	0	1	1	0	0	1	0	1	1	1	1	1	2	1	1	1	0	1	0	1	0	1	0	1	?
Choneziphius	?	1	?	?	2	1	1	0	a	1	?	?	?	?	?	?	?	?	?	?	?	?	0	1	0	?
Tusciziphius	?	1	?	?	3	1	1	1	1	1	?	?	1	2	?	?	?	?	?	?	?	?	0	0	0	?
Globicetus	?	1	?	?	3	1	1	1	1	1	?	?	?	?	?	?	?	?	?	?	?	?	0	0	0	?
Imocetus	?	1	?	?	2	1	1	1	2	1	?	?	?	?	?	?	?	?	?	?	?	?	0	0	0	?
Beneziphius	?	a	?	?	2	0	1	0	0	?	?	?	?	?	?	?	?	?	?	?	?	?	0	1	0	?
Ziphirostrum	?	a	?	?	1	0	1	0	0	1	?	?	?	?	?	?	?	?	?	?	?	?	0	0	0	?
Aporotusrec	?	1	?	?	2	0	1	0	0	1	?	?	?	?	?	?	?	?	?	?	?	?	0	0	0	?
Aporotusdicyrt	?	?	?	?	2	0	1	0	0	1	?	?	?	?	?	?	?	?	?	?	?	?	1	0	0	0
Messapicetus	1	0	2	0	1	0	1	0	0	1	1	1	1	2	0	1	1	1	1	?	?	0	a	0	0	1
Ninoziphius	?	0	2	0	0	0	1	0	0	1	1	0	1	2	0	1	1	1	1	?	0	0	0	0	0	?
Chimuziphius	?	?	?	?	1	0	1	0	0	1	?	?	?	?	?	?	?	?	?	?	?	1	0	0	0	?
Nenga	?	1	?	?	0	0	1	0	1	1	?	?	?	?	?	?	?	?	?	?	?	?	1	0	0	?
Notoziphius	?	0	?	?	0	0	1	0	0	1	?	?	1	2	?	?	?	?	?	?	?	1	0	0	0	?
Caesuziphius	?	0	?	?	0	0	1	0	0	1	?	?	1	2	?	?	0	0	1	?	?	1	0	0	0	?

Equally weighting
Number of equally parsiomonious trees = 19
CI = 0.497
RI = 0.767

Strict consensus of 19 trees:

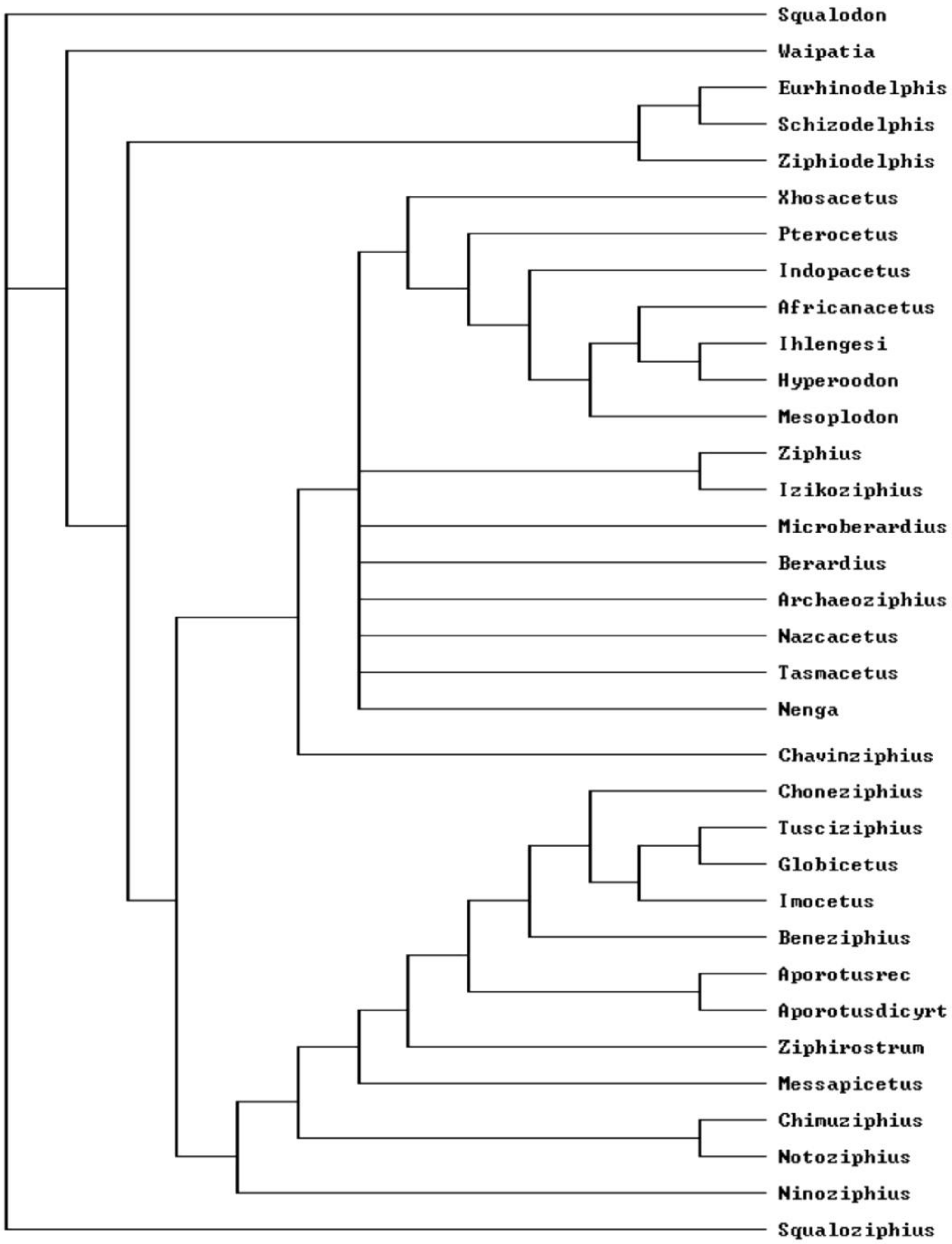

K = 1
Number of equally parsiomonious trees = 3
CI = 0.485
RI = 0.756
Goloboff fit = - 33.927
GC = 1138

Strict consensus of 3 trees:

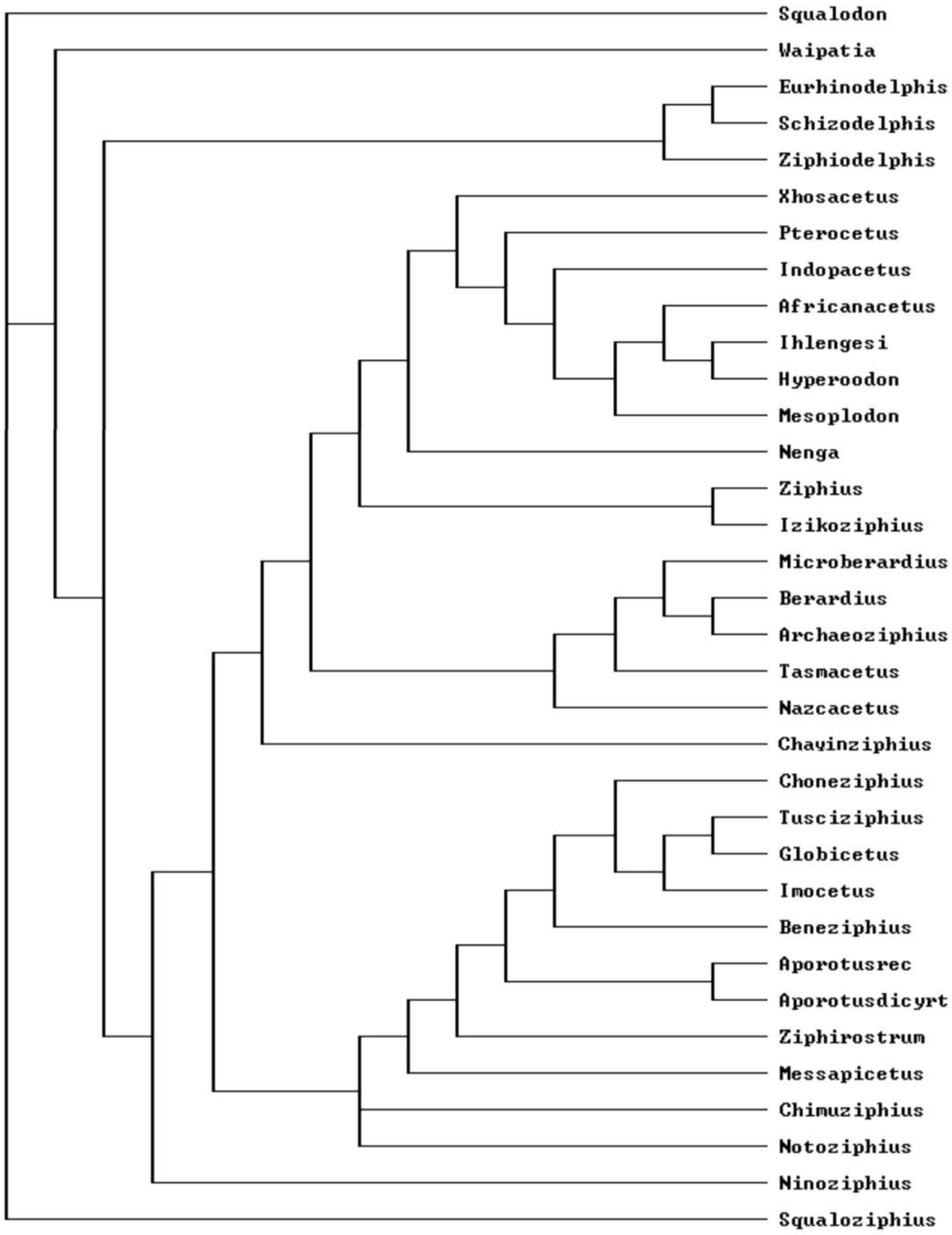

K = 2
Number of equally parsiomonious trees = 9
CI = 0.483
RI = 0.753
Goloboff fit = - 36.568
GC = 1142

Strict consensus of 9 trees:

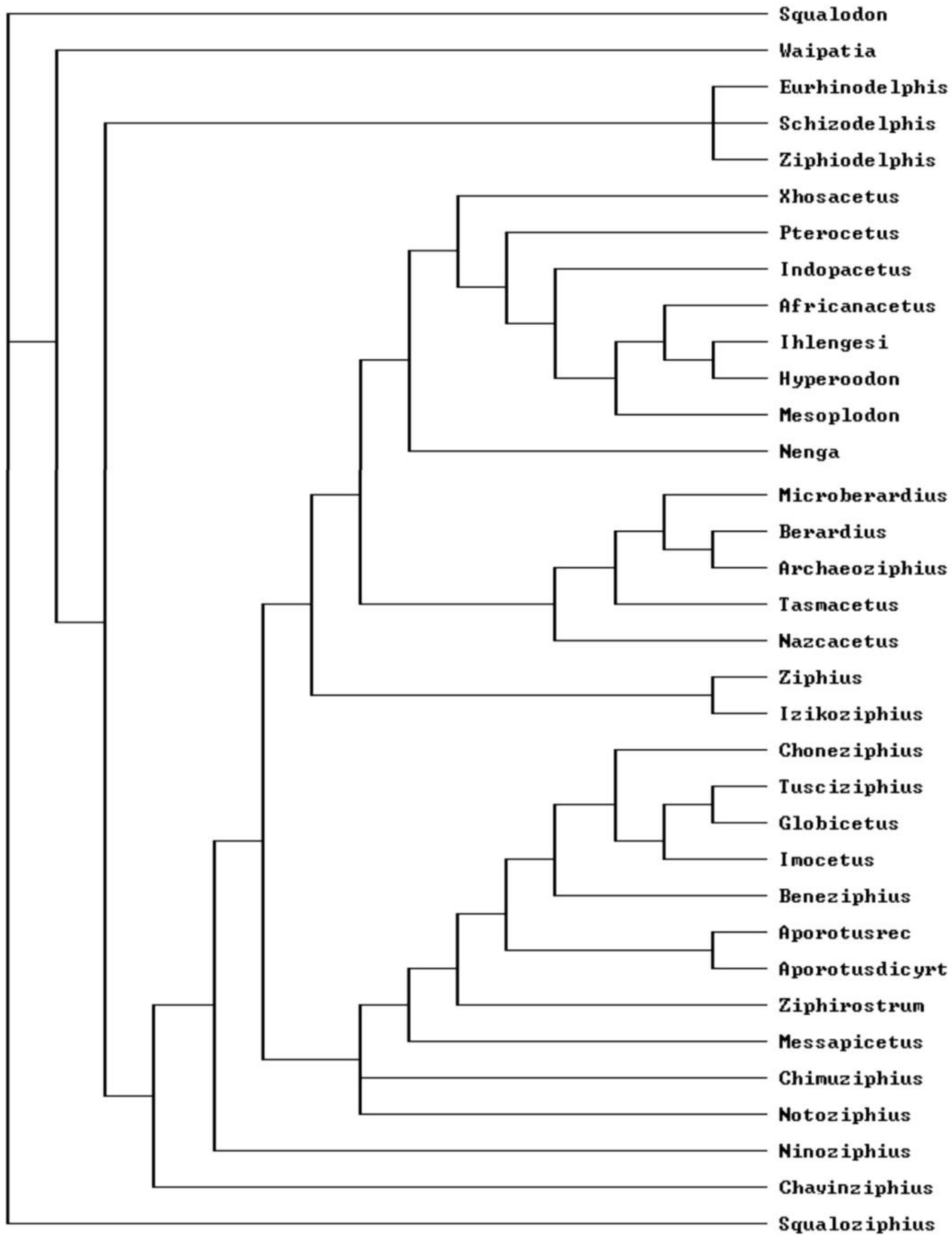

K = 3-15
Number of equally parsiomonious trees = 1
CI = 0.494
RI = 0.764
Goloboff fit = - 38.668 / - 45.713
GC = 1127

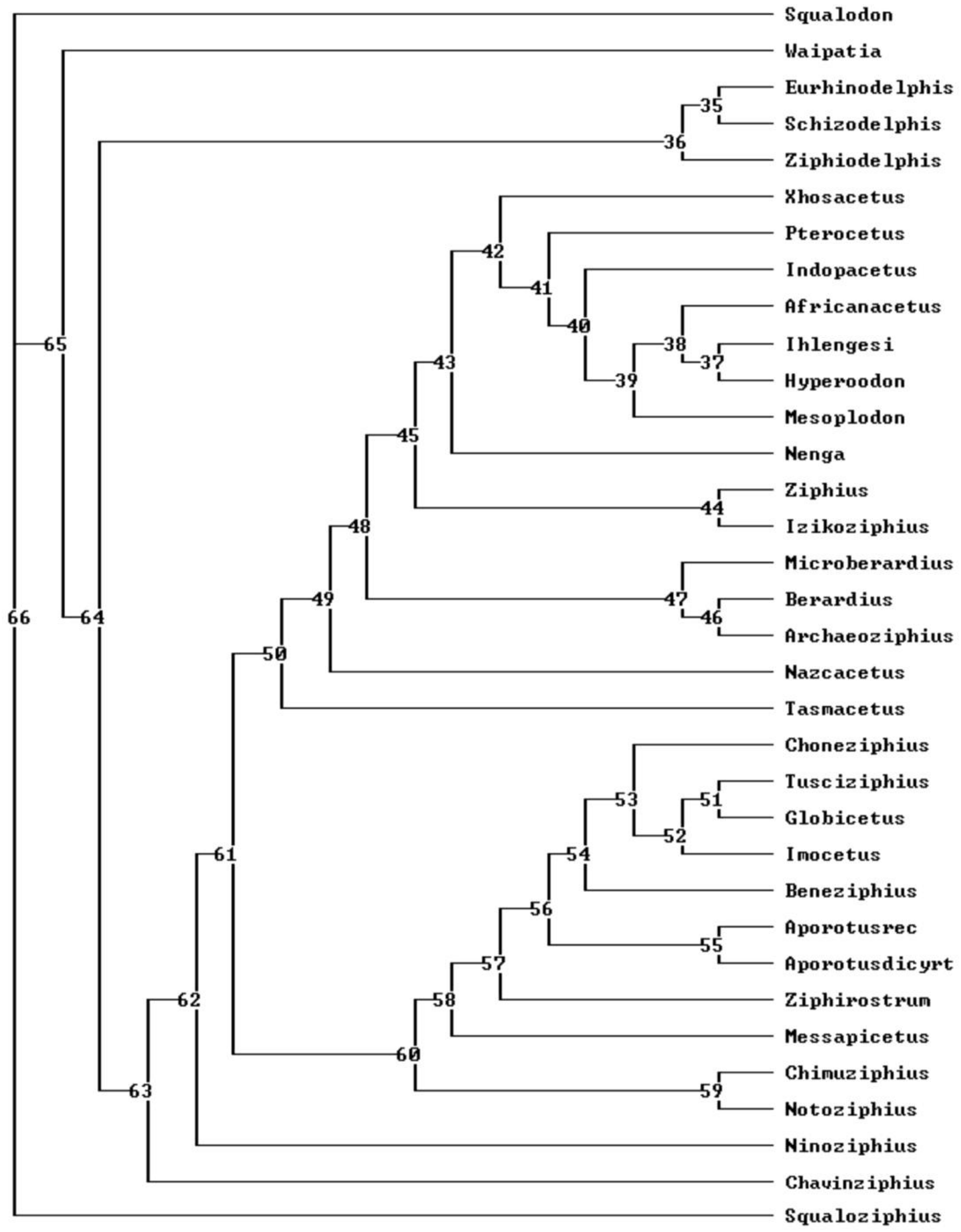

K = 16-1405
Number of equally parsiomonious trees = 7
CI = 0.497
RI = 0.767
Goloboff fit = - 45.900 / - 49.940
GC = 1053

Strict consensus of 7 trees:

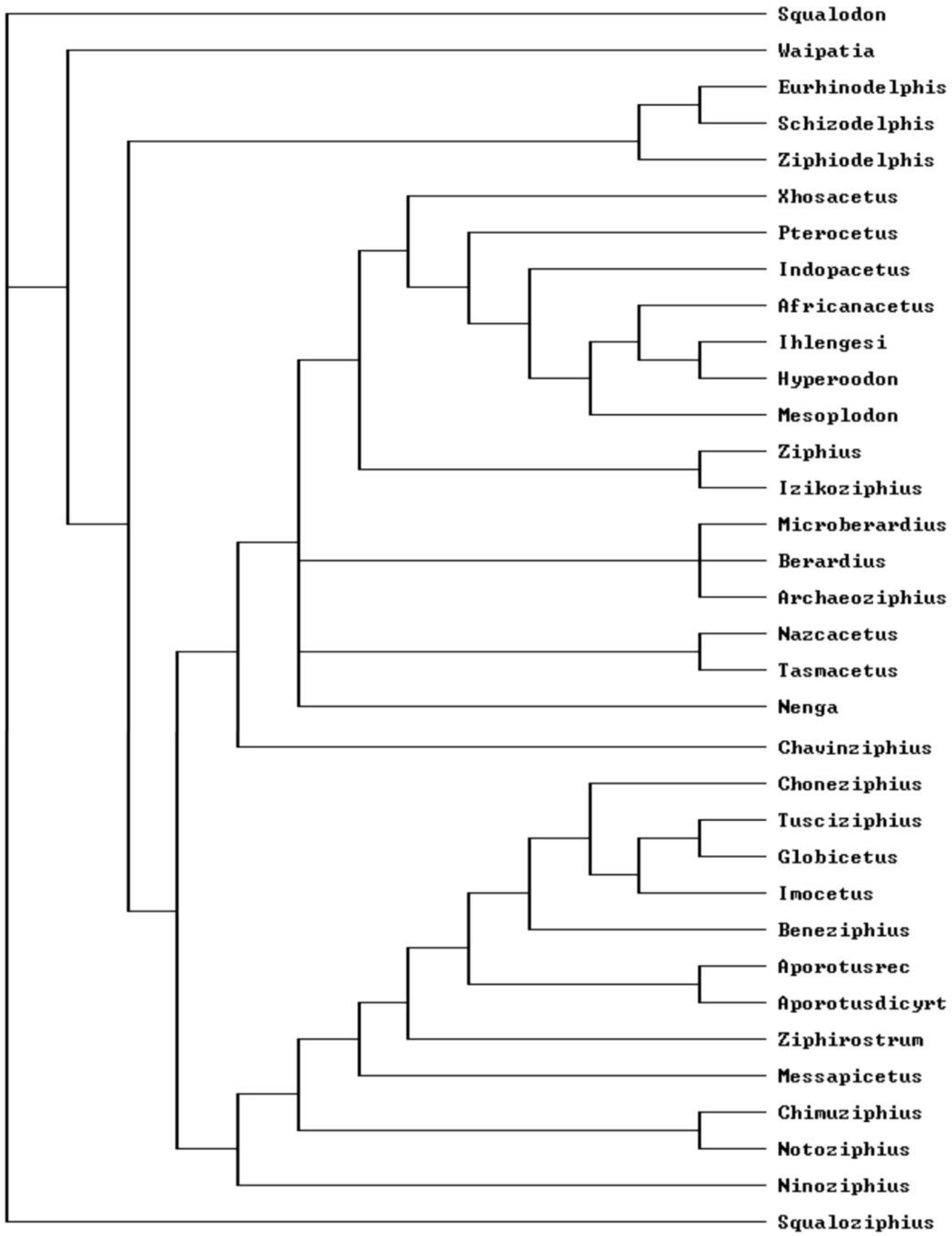

References

Bianucci G, Lambert O, Post K. 2007. A high diversity in fossil beaked whales (Odontoceti, Ziphiidae) recovered by trawling from the sea floor off South Africa. *Geodiversitas* **29**:5–62.

Bianucci G, Lambert O, Post K. 2010. High concentration of long-snouted beaked whales (genus *Messapicetus*) from the Miocene of Peru. *Palaeontology* **53**:1077–1098.

Fordyce RE. 1994. *Waipatia maerewhenua*, new genus and new species (Waipatiidae, new family), an archaic late Oli gocene dolphin from New Zealand. In: Berta A, Deméré TA, eds. *Contributions in marine mammal paleontology honoring Frank C. Whitmore, Jr. Proceedings of the San Diego Society of Natural History*. Vol. 29. San Diego: San Diego Natural History Museum, 147–178.

Fordyce RE, Quilty PG, Daniels J. 2002. *Australodelphis mirus*, a bizarre new toothless ziphiid-like fossil dolphin (Cetacea: Delphinidae) from the Pliocene of Vestfold Hills, East Antarctica. *Antarctic Science* **14**:37–54.

Geisler JH, Sanders AE. 2003. Morphological evidence for the phylogeny of Cetacea. *Journal of Mammalian Evolution* **10:** 23–129.

Lambert O. 2005a. Systematics and phylogeny of the fossil beaked whales *Ziphirostrum* du Bus, 1868 and *Choneziphius* Duvernoy, 1851 (Cetacea, Odontoceti), from the Neogene of Antwerp (North of Belgium). *Geodiversitas* **27**:443–497.

Lambert O. 2005b. Phylogenetic affinities of the longsnouted dolphin *Eurhinodelphis* (Cetacea, Odontoceti) from the Miocene of Antwerp. *Palaeontology* **48**:653–679.

Lambert O, de Muizon C, Bianucci, G. 2013. The most basal beaked whale *Ninoziphius platyrostrisMuizon*, 1983: Clues on the evolutionary history of the family Ziphiidae (Cetacea: Odontoceti). *Zoological Journal of the Linnean Society* **167(4)**:569-598 DOI 10.1111/zoj.12018

Lambert O, de Muizon C, Bianucci, G. 2015. A new archaic homodont toothed whale (Mammalia, Cetacea, Odontoceti) from the early Miocene of Peru. *Geodiversitas* **37**:79–108 DOI 10.5252/ g2015n1a4

Mead JG. 2007. Stomach anatomy and use in defining systematic relationships of the cetacean family Ziphiidae(beaked whales). *The Anatomical Record* **290**:581–595.

Muizon C de. 1991. A new Ziphiidae (Cetacea) from the Early Miocene of Washington State (USA) and phylogenetic analysis of the major groups of odontocetes. *Bulletin du Muséum National d'Histoire Naturelle, Paris* **12:**279–326.
