# Supplementary material for: New beaked whales from the late Miocene of Peru and evidence for convergent evolution in stem and crown Ziphiidae (Cetacea, Odontoceti)
Source: PeerJ. 2016 Sep 20;4:e2479. doi: 10.7717/peerj.2479 (PMC5036081; doi:10.7717/peerj.2479)
Supplement: Supplemental Information 2 — Ontogenetic change of size and number of dorsal infarorbital foramina on the maxilla near the base of the rostrum in Berardius spp. [file peerj-04-2479-s002.pdf]

Supplemental File S2

to accompany the article:

New beaked whales from the late Miocene of Peru and evidence for convergent evolution in stem and crown Ziphiidae (Cetacea, Odontoceti)

Giovanni Bianucci [1], Claudio Di Celma [2], Mario Urbina[3], Olivier Lambert[4]

[1] Dipartimento di Scienze della Terra, Università di Pisa, Via Santa Maria, 53, Pisa 56126, Italy
[2] Scuola di Scienze e Tecnologie, Università di Camerino, Camerino 62032, Italy
[3]Departamento de Paleontologia de Vertebrados, Museo de Historia Natural-UNMSM, 1256, Avenida Arenales, Lima 14, Peru
[4]Institut royal des sciences naturelles de Belgique, D.O. Terre et Histoire de la Vie, 29, rue Vautier, Brussels 1000, Belgium

published in PeerJ

Ontogenetic change of size and number of dorsal infarorbital foramina on the maxilla near the base of the rostrum in *Berardius* spp.

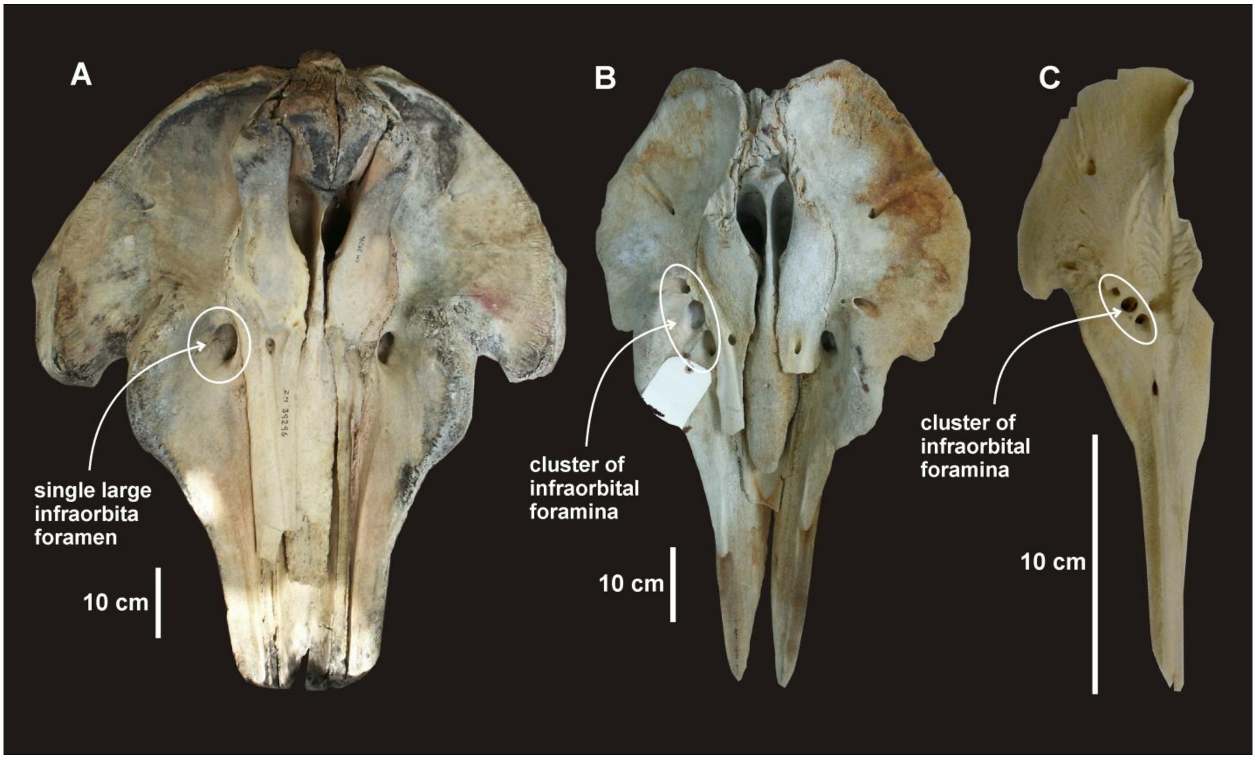

Comparison of incomplete skulls in dorsal view of *Berardius arnouxii* and *B.bairdii* at different ontogenetic stages, evidencing variations in size and number ofdorsal infraorbital foramina near the base of the rostrum.

A, *B. arnouxii* SAM 39296, adult; B, *B. arnouxii* SAM 37404, immature; C, fetus (body length 183 cm) associated to female *B. bairdii* USNM 55089.

SAM = Iziko South African Museum, Cape Town, South Africa; USM = National Museum of Natural History, Smithsonian Institution, Washington D.C., U.S.A.
